# Data on the evolutionary history of the V(D)J recombination-activating protein 1 – RAG1 coupled with sequence and variant analyses

**DOI:** 10.1016/j.dib.2016.05.021

**Published:** 2016-05-20

**Authors:** Abhishek Kumar, Anita Bhandari, Sandeep J. Sarde, Sekhar Muppavarapu, Ravi Tandon

**Affiliations:** a Department of Genetics & Molecular Biology in Botany, Institute of Botany, Christian-Albrechts-University at Kiel, Kiel, Germany; b Division of Molecular Genetic Epidemiology German Cancer Research Center, Heidelberg, Germany; c Molecular Physiology, Institute of Zoology, Christian-Albrechts-University at Kiel, Kiel, Germany; d Agrigenomics, Christian-Albrechts-University at Kiel, Kiel, Germany; e School of Biotechnology, Jawaharlal Nehru University, New Delhi, India

## Abstract

RAG1 protein is one of the key component of RAG complex regulating the V(D)J recombination. There are only few studies for RAG1 concerning evolutionary history, detailed sequence and mutational hotspots. Herein, we present out datasets used for the recent comprehensive study of RAG1 based on sequence, phylogenetic and genetic variant analyses (Kumar et al., 2015) [1]. Protein sequence alignment helped in characterizing the conserved domains and regions of RAG1. It also aided in unraveling ancestral RAG1 in the sea urchin. Human genetic variant analyses revealed 751 mutational hotspots, located both in the coding and the non-coding regions. For further analysis and discussion, see (Kumar et al., 2015) [1].

**Specifications Table**

**Table:** 

Subject area	*Biology*
More specific subject area	*Molecular evolution and bioinformatics*
Type of data	*Tables, figures*
How data was acquired	*Retrieved from public databases*
Data format	*Analyzed data*
Experimental factors	*RAG1 sequences were retrieved from ENSEMBL and/or NCBI database.*
Experimental features	*RAG1 protein alignment using Muscle tool and edited in the GeneDoc*
	*RAG1 Variants were analyzed with SIFT, Polyphen &* rSNPbase
Data source location	*Germany*
Data accessibility	Data is with this article

**Value of the data**

• Protein sequence analysis data reveal that SpRAG1L possesses only 19–20% identities with vertebrate RAG1, which helped us in deriving an ancestral RAG1 protein in sea urchin. This approach can be used the detection of origins for different proteins.

• Protein sequence alignment locates two major domains and several regions of RAG1, which suggested that these fragments were conserved from sea urchin to human. This hints evolutionary conservation of protein domains in the protein of interest and their ancestors.
• Data on the genetic variant analysis suggests that human RAG1 gene has 751 variants.
• Furthermore, there are 267 missense variants of human RAG1 causes change in amino acids including 140 deleterious mutations. These variant data serve as the mutational hotspots within the coding region of human RAG1. Assessment of mutational hotspot for any protein is critically important for understanding its function and roles in diseases.
• Additionally, 284 non-coding variants were identified with 94% regulatory in nature, which are often called as regulatory SNP (rSNP). These data are source of regulatory implications flanking any given gene.

## 1 Data

Table 1 lists all RAG1 sequences used in Kumar et al. [1] and these sequences are used for constructing protein sequence alignment of RAG1 (Fig. S1). This protein alignment is the basis for the Figs. 2 and 3 and Table 1–5 of Kumar et al. [1]. Details of human RAG1 variants are summarized in the Table S1 and regulatory SNPs in the Table S2. These two supplementary tables are primary data for variant analyses described in Fig. 4 and Tables 2–5 of Kumar et al. [1].

## 2 Experimental design, materials and methods

Using the BLAST homology detection tool [2], we extracted RAG1 gene from vertebrate genomes listed either in Ensembl release 77 [3] or NCBI. To ensure accuracy of gene structures, we combined the gene predictions of the Ensembl [3] and AUGUSTUS tool [4]. We used human RAG1 as the standard sequence for intron position mapping and numbering of intron positions, followed by suffixes a–c for their location as reported previously [5]. We aligned selected RAG1 protein sequences using MUSCLE tool [6] with and we manually adjusted alignment with GENEDOC tool [7]. We reconstructed a phylogenetic tree with maximum likelihood method, based on the JTT matrix-based model [8] with 1000 bootstrap replicates. We imported all consensus trees to MEGA 6 software [9], where we edited and visualized these trees as per requirement. To detect the orthologs of RAG1 gene, we analyzed micro-synteny across different genomes using two genome browsers namely, NCBI map viewer [10] and ENSEMBL genome browser [11], [12]. Furthermore, we generated human RAG1 variants from 1092 human genomes from 14 different populations available in 1000 genomes project [13]. We analyzed the impact assessments of missense variants on the human RAG1 protein using SIFT [14] and PolyPhen V2 [15] tools, as described previously [16], [17], [18], [19]. We detected regulatory nature of non-coding variants using the rSNPbase (this database provides reliable, and comprehensive regulatory annotations [20] and such variants are called regulatory SNP or rSNP).

## Figures and Tables

**Table 1:** Summary of RAG1 from selected animal genomes**.** This data is collected from Ensembl database release 77 . At times data is gathered from additional databases as indicated.

**Name**	**Organism**	**Species**	**Accession id**	**Chromosomal localization**
HsapRAG1	Human	*Homo sapiens*	ENSG00000166349	Chromosome 11: 36,532,259-36,614,706
MmusRAG1	Mouse	*Mus musculus*	ENSMUSG00000061311	Chromosome 2: 101,638,282-101,649,501
RnorRAG1	Rat	*Rattus norvegicus*	ENSRNOG00000004630	Chromosome 3: 97,866,048-97,877,145
TgutRAG1	Zebrafinch	*Taeniopygia guttata*	ENSTGUG00000010147	Chromosome 5: 17,596,747-17,599,869
MgalRAG1	Turkey	*Meleagris gallopavo*	ENSMGAG00000015794	Chromosome 5: 19,778,620-19,781,748
PsinRAG1	Turtle	Pelodiscus sinensis	ENSPSIG00000001811	Scaffold JH209124.1: 1,890,899-1,894,018
DrerRAG1	Zebrafish	*Danio rerio*	ENSDARG00000052122	Chromosome 25: 9,231,637-9,238,142
TrubRAG1	Fugu	*Takifugu rubripes*	ENSTRUG00000001340	scaffold_302: 189,544-193,510
TnigRAG1	Tetraodon	*Tetraodon nigroviridis*	ENSTNIG00000012168	Chromosome 13: 5,598,243-5,602,176
OnilRAG1	Tilapia	*Oreochromis niloticus*	ENSONIG00000014593	Scaffold GL831142.1: 1,924,501-1,931,477
GmorRAG1a	Cod	*Gadus morhua*	ENSGMOG00000003395	GeneScaffold_2196: 249,630-253,939
XmacRAG1	Platyfish	*Xiphophorus maculatus*	ENSXMAG00000000820	Scaffold JH556735.1: 897,221-901,222
GacuRAG1	Stickleback	*Gasterosteus aculeatus*	ENSGACG00000011465	groupXIX: 14,493,756-14,497,787
OlatRAG1	Medaka	*Oryzias latipes*	ENSORLG00000011969	Chromosome 6: 17,343,305-17,347,405
LchaRAG1	Coelacanth	*Latimeria chalumnae*	ENSLACG00000004406	Scaffold JH126568.1: 121,275-124,451
VpaRAG1	Alpaca	*Vicugna pacos*	ENSVPAG00000008826	GeneScaffold_2429: 269,595-273,365
AcarRAG1	Anole lizard	Anolis carolinensis	ENSACAG00000005106	Chromosome 1: 53,518,235-53,521,375
DnoRAG1	Armadillo	Dasypus novemcinctus	ENSDNOG00000006294	Scaffold JH582431.1: 4,276,543-4,279,674
OgarRAG1	Bushbaby	Otolemur garnettii	ENSOGAG00000027339	Scaffold GL873520.1: 63,167,240-63,168,052
FcatRAG1	Cat	Felis catus	ENSFCAG00000002908	Chromosome D1: 92,125,946-92,129,077
AmeRAG1	Cave fish	Astyanax mexicanus	ENSAMXG00000017587	Scaffold KB871579.1: 5,211,103-5,217,994
PtroRAG1	Chimpanzee	Pan troglodytes	ENSPTRG00000003512	Chromosome 11: 36,559,562-36,571,320
BtauRAG1	Cow	Bos taurus	ENSBTAG00000040293	Chromosome 15: 67,827,233-67,830,364
CfamRAG1	Dog	Canis lupus familiaris	ENSCAFG00000006808	Chromosome 18: 31,631,533-31,634,664
TtruRAG1	Dolphin	Tursiops truncatus	ENSTTRG00000014075	scaffold_110171: 196,070-199,540
AplaRAG1	Duck	Anas platyrhynchos	ENSAPLG00000011756	Scaffold KB742537.1: 887,774-890,899
LafrRAG1	Elephant	Loxodonta africana	ENSLAFG00000023175	SuperContig scaffold_21: 12,902,400-12,905,531
MfurRAG1	Ferret	Mustela putorius furo	ENSMPUG00000019963	Scaffold GL896949.1: 10,184,818-10,187,949
FalbRAG1	Flycatcher	Ficedula albicollis	ENSFALG00000014372	Scaffold JH603235.1: 3,494,497-3,497,619
NleuRAG1	Gibbon	Nomascus leucogenys	ENSNLEG00000017951	SuperContig GL397264.1: 51,275,048-51,286,754
GgorRAG1	Gorilla	Gorilla gorilla gorilla	ENSGGOG00000013132	Chromosome 11: 37,187,229-37,198,984
CporRAG1	Guinea Pig	Cavia porcellus	ENSCPOG00000004516	scaffold_92: 2,485,274-2,488,405
EcabRAG1	Horse	Equus caballus	ENSECAG00000021936	Chromosome 12: 3,025,356-3,033,251
PcapRAG1	Hyrax	Procavia capensis	ENSPCAG00000001732	GeneScaffold_5497: 13,553-16,990
MmulRAG1	Macaque	Macaca mulatta	ENSMMUG00000018267	Scaffold 1099214286323: 4,563-7,694
CjacRAG1	Marmoset	Callithrix jacchus	ENSCJAG00000011082	Chromosome 11: 99,857,897-99,869,593
MlucRAG1	Microbat	Myotis lucifugus	ENSMLUG00000000544	Scaffold GL430055: 356,167-359,298
MmurRAG1	Mouse Lemur	Microcebus murinus	ENSMICG00000008611	GeneScaffold_3288: 841,983-845,201
MdomRAG1	Oppossum	*Monodelphis domestica*	ENSMODG00000024470	Chromosome 5: 272,756,599-272,759,742
AmelRAG1	Panda	Ailuropoda melanoleuca	ENSAMEG00000019378	Scaffold GL193442.1: 461,741-464,872
SscrRAG1	Pig	Sus scrofa	ENSSSCG00000026145	Chromosome 2: 26,730,010-26,738,216
OanaRAG1	Platypus	Ornithorhynchus anatinus	ENSOANG00000011770	Chromosome 3: 11,364,602-11,365,783
OcunRAG1	Rabbit	Oryctolagus cuniculus	ENSOCUG00000006989	Chromosome 1: 175,828,096-175,831,224
OarRAG1	Sheep	Ovis aries	ENSOARG00000010441	Chromosome 15: 65,210,839-65,213,970
SaraRAG1	Shrew	Sorex araneus	ENSSARG00000010950	GeneScaffold_5915: 66,723-69,956
LocRAG1	Spotted gar	*Lepisosteus oculatus*	ENSLOCG00000001283	Chromosome LG27: 1,403,519-1,420,074
ItriRAG1	Squirrel	Ictidomys tridecemlineatus	ENSSTOG00000025584	Scaffold JH393343.1: 1,817,576-1,820,707
TsyrRAG1	Tarsier	Tarsius syrichta	ENSTSYG00000007158	scaffold_7240: 21,771-24,902
SharRAG1	Tasmanian devil	Sarcophilus harrisii	ENSSHAG00000014085	Scaffold GL864890.1: 1,391,366-1,394,509
TbeRAG1	Tree Shrew	Tupaia belangeri	ENSTBEG00000003010	GeneScaffold_4067: 865-4,810
MeuRAG1	Wallaby	Macropus eugenii	ENSMEUG00000003165	Scaffold77145: 3,005-5,812
XtroRAG1	Xenopus	Xenopus tropicalis	ENSXETP00000016443/XP_002937338^a^	Scaffold GL172917.1: 903,952-910,208
SpuRAG1L	Sea urchin	Strongylocentrotus purpuratus	AAZ23546.1^a^	NA

a From NCBI.

## Appendix A Supplementary material

Supplementary material **Fig. S1. Protein sequence alignment of RAG1 from selected animal genome.** Different domains and regions are marked above the alignment. Sequence identity scores of ≥85%, ≥65% and ≥45% are marked by red, blue and green shades respectively.

Supplementary material **Table S1. Overview of 751 germline variants of RAG1 gene deduced from 1000 genome data.**

Supplementary material **Table S2. Overview of non-coding germline variants of Human RAG1.**

## Footnotes

Appendix A Supplementary data associated with this article can be found in the online version at 10.1016/j.dib.2016.05.021.
