# Supplementary material for: Data on the evolutionary history of the V(D)J recombination-activating protein 1 – RAG1 coupled with sequence and variant analyses
Source: Data Brief. 2016 May 20;8:87–92. doi: 10.1016/j.dib.2016.05.021 (PMC4887553; doi:10.1016/j.dib.2016.05.021)
Supplement: Supplementary file 1 — Supplementary material Fig. S1. Protein sequence alignment of RAG1 from selected animal genome. Different domains and regions are marked above the alignment. Sequence identity scores of ≥85%, ≥65% and ≥45% are marked by red, blue and green shades respectively. [file mmc1.pdf]

Fig. S1. Protein sequence alignment of RAG1 from selected animal genome. Different domains and regions are marked above the alignment. Sequence identity scores of ≥85%, ≥65% and ≥45% are marked by red, blue and green shades respectively.

```
                                  -------------------------N-terminal end-----------------------
RAG1-Human                    1 -MAASFPPTLGLSS-APDEIQHPHIKFSEWKFKLFRVRSFEKT-P-EEAQKEKKDSF--E     54
RAG1-Opossum                  1 -MEISVAPSQGLRP-VPDEIQYPHMKFSEWKFKLFKVRSSVKP-SPGEAQGPSKNTM--E     55
RAG1-Turkey                   1 -MSITSQMD------LPEEIQHPYAKFSEWKFKLFKVRPFQKA-PSDKSQCINKDQE--Q     50
RAG1-Turtle                   1 ---------MSLHT-MPEEIQHPYTKFSEWKFKLFKVRSFEKT-PSGDSQVANKEKA--E     47
RAG1-Lizard                   1 -METLPPPSMNLCF-VSEQTQLPYKKFAEWKFKLFKVRSIEK--PLPDGSHLANNN---K     53
RAG1-Xenopus                  1 -MEVALPN-------VPTEIHRPFTKFSEWKFKLFRVKSLERR-PSEEETEGSEVSYSSS     51
RAG1-Cod                      1 TMAVERPETDCPRSSMPDELQHPHLKFSHWKFKLFRVRSTDED-TMASETAPRIAAP--P     57
RAG1-Coelacanth               1 -MEVPLV-SMPLRN-MPAELHHPYKKFSEWKFKLFRVRSFEKTDPVEVETSNKENKP--S     55
RAG1-Zebrafish                1 MEKGRWSSEDAPRASMPDELSHP--KFSEWKFKLFRVRSMEKA-PVQNETPVEK------     51
RAG1-Tetraodon                1 -MAEACLETDDPRSTMPAELHHPHSKYSQWKFKLFRVRSMEKA-PLPSETLTEKGVL--L     56
RAG1-Stickleback              1 IMAEESLETDGHRSSMPAELCHPHSKYSQWKFKLFRLKSMEKV-PLPTETRPEEGAL--S     57
RAG1-Tilapia                  1 -MAAESLETDGPRSSMPPELHHPHSEYSQWKFKLFRVKSMEKA-PLPSETQHE-------     51
RAG1-Platyfish                1 -MAAENPETDGPRSFMPAELQHPHSKYSQWKFKLFRVKSMEKA-PLPSETQTEIGAS--L     56
RAG1L-Strongylocentrotus      - ------------------------------------------------------------      -

                                ------------------------------------------|------------------
RAG1-Human                   55 GKPSLEQSPAVLDKADG---------QKPVPTQPLLKAHPKFSKKFHDNEKARGKAIHQA    105
RAG1-Opossum                 56 GQSSLEETPVVICNTNI------PAPEKPALVQPTFTANSKSLEKPADIGKAGEKAIHQA    109
RAG1-Turkey                  51 EVASTDQNITLHKDEEV------PRGEKLILTQMDFMGNTQALEKNVNDMKIQDNTVHQN    104
RAG1-Turtle                  48 EVVSLDKGTVVQKDAAV------PQAEKPLLADAELKHSKQALEKDADNLKMKENAAHQA    101
RAG1-Lizard                  54 GKVAASLDQVIEEKTDM-----VALSHSPFETDTELNKSIQTIDKGAFHVSQREAETHQE    108
RAG1-Xenopus                 52 QETSPESTVVLEDLSLG-----STPQSPSNFKPQEASEKSNEVDNNETDLKSLGEETHIT    106
RAG1-Cod                     58 EVLPAPAAAAAIDPWSGEEGHLGLPGSVMKLCLGGKSKENVEGPGHRVDRKLQEMDLHME    117
RAG1-Coelacanth              56 HAAPLEATPLVIQHKES----------AALENIPPAGLKPIFVDNNQLEPNDMKLQTHIA    105
RAG1-Zebrafish               52 ----ENQPELAMEKTSS-------QGSVMRLCFGGKSKENVESARGRVDLKLQEIDTHMN    100
RAG1-Tetraodon               57 GLSAPAAPNTELGNGTA-------PGGVMKLCLGRKSKENVEGLSTRMDLKLQEMDAHMN    109
RAG1-Stickleback             58 GIPPPAALNIEVDNGTG-------AESVMKLCLGGKSKENVEGPGRRVDMKLQEMDTHMN    110
RAG1-Tilapia                 52 -ISPTAPPNIEIDHAVD-------PGSVMKLCLGGKSKENVEGPGRRVDVKLQEMETHMD    103
RAG1-Platyfish               57 GISPPTAPNIEADHGGG-------LASVMKLCLGGKSKENVEGAGKRVDMKLQEMDTHMN    109
RAG1L-Strongylocentrotus      1 ---------------------------------------------------MEIVELHRK      9
```

```
                                  ---------------------------CND------------------------------
RAG1-Human                   106  NLRHLCRICGNSFRADEHNRRYPVHGPVDGKTLGLLRKKEKRATSWPDLIAKVFRIDVKA  165
RAG1-Opossum                 110  SLRQLCRICGASFRADGQNRRYPVHGPVDSKTQGILRKKEKKVTSWPDLIAKVFKIDVKG  169
RAG1-Turkey                  105  NLKQLCRICGASFKTDCYKRTHPVHGPVDDETLCLLRKKEKKATSWPDLIAKVFKIDVRG  164
RAG1-Turtle                  102  NLQQLCRVCGVSFKTDRYKRSHPVHGPVDSETQGVLRKKEKKATSWPDLIAKVFKIDVRA  161
RAG1-Lizard                  109  NLQHLCRICGGSFKTDPYKRSHPVHGPIDDDMKALLRKKEKKATSWPDLLAKVLKIDVKG  168
RAG1-Xenopus                 107  AIQQLCRICGASFKMDQQNRSYPVHGPVDSETQDVLRRREKKVTSWPELILKVFKTDVRA  166
RAG1-Cod                     118  KLRCLCRLCGSALRK-VKGPERAVQGGLGESCRHALSRMGCKFTTWPDVIHKVFKVDVTG  176
RAG1-Coelacanth              106  NLQRLCRLCGDCLKTDGRNMSFAIQGPVDKSTAVLLRKMRVKISSWPDLILKVFSTDVRG  165
RAG1-Zebrafish               101  LLKNMCRLCGIAIQK-AKGPSHEVQGVLEESSRCALRRMGCKLVTWPEVILKVFKVDVTT  159
RAG1-Tetraodon               110  QLRCLCRLCGMMLRK-VKGPVHDVQGDLDDTSRGTLRKMGCKLTSWPEVIHKVFKVDVTE  168
RAG1-Stickleback             111  HLRCLCRLCGMALRT-VKGPVHDVNGDLDEVSKGALRKMGCKFTSWPEVILKVFKVDVKE  169
RAG1-Tilapia                 104  HLRGLCRVCGMVLRK-VKGPVHDVHGDLDEVSKCALRKMGCKFPSWPEVILKVFKVDVTE  162
RAG1-Platyfish               110  HLRCLCRICGMVLRK-VKGPVHDVHGDLDDASKCVLRKMGCKFTSWPDILLKVFKVDVTE  168
RAG1L-Strongylocentrotus      10  ALSQTCRVCGSYVKN----------------------KKSLSSKEKYEELILSVYGIDFKL   48


                                  ---------------------------CND------------------------------
RAG1-Human                   166  DVDSIHPTEFCHNCWSIMHRKFSS-APCEVYFPRNVTME---WHPHTPSCDICNTA----  217
RAG1-Opossum                 170  DNDSIHPTQFCHNCWNIMYGRFSN-SPHEVYFPRNATIE---WHPHTPSCDICQAA----  221
RAG1-Turkey                  165  DVDTIHPTRFCHNCWSIIHRKFSN-TPCEVYFPRNSTME---WQPHSTNCEVCHTP----  216
RAG1-Turtle                  162  DIDTIHPTRFCHNCWSIIHRKFSN-APCEVYFPRNSTME---WKSHSPNCTVCCTA----  213
RAG1-Lizard                  169  DIDTIHPTKFCHQCWTVIQKKLSN-SPYEIFFPKKGPME---WQPHSTSCDVCGTS----  220
RAG1-Xenopus                 167  DVDTIHPTRFCHNCWTIMNQKFSH-NSSEVYFPHNQAVE---WTPHSATCHVCHSSK---  219
RAG1-Cod                     177  DLESVHPPSFCQRCWMTVMR------GGGFCSFTPLRVPE---WTPHSALCSLCHARKTSS  228
RAG1-Coelacanth              166  DIKSVHPTHFCHTCWTVIRKWISDPNGQEMGVLADRVVG---WLPHSLFCKICDTG----  218
RAG1-Zebrafish               160  DMETVHPSLFCHRCWTAAIR------GGGFCSFTNTRIPD---WKPHTSQCNLCFPKK-SS  210
RAG1-Tetraodon               169  DIEAVHPLFFCHRCWMTAIR------GGGVCSFSRIRVPE---WKPHSSHCELCSPRK-AS  219
RAG1-Stickleback             170  DTESVHPLSFCHRCWVVTIR------GGGFCSFSRTRVPE---WKPHSSTCNLCSPRK-PS  220
RAG1-Tilapia                 163  DTESVHPLSFCHRCWMVAIR------GGGVCSFTRTRVPE---WKPHSSLCHLCYPKK-TS  213
RAG1-Platyfish               169  DAESIHPLSFCHRCWLAAIR------GGGVCSFTRTKVPE---WKPHTSHCYLCSPK----  216
RAG1L-Strongylocentrotus      49  DDEDVHPPRICVSCRLWMTRSDSRNAEGPTYPTSGKTLANFSAHPELEPCSICEAYQATK  108
```

```
                                   ---------------------------------------------------------------
RAG1-Human                   218  --RRGLKRKSLQPNLQ-LSKKLKTVLDQARQ-------------------ARQHKRRAQAR    256
RAG1-Opossum                 222  --RWGLKRKGRQSNPS-LSKKLKAMADRARR-------------------ARLPKSQAQPR    260
RAG1-Turkey                  217  --RRGVKRKSQPPNVQ-HGKRVKITAERARV-------------------SRGIKNQVL--    253
RAG1-Turtle                  214  --PRGVKRKNQPSNLQ-LGKKLKIMAERARK-------------------TKGVKKQSQ--    250
RAG1-Lizard                  221  --PRGVKRKKQVLNPQ-LNKKMRMMAGRARK-------------------IRQIRNTKQ--    257
RAG1-Xenopus                 220  --PWGKRKGAPQLNPH-KMKRRKTGSEFAKK-------------------SKASSNHSIQR    258
RAG1-Cod                     229  FQRLGRKRKKARGKT--LAKRCKWDPSDGTSSSDRRPLRPVSQHRHHHPGPGLQPQASTR    286
RAG1-Coelacanth              219  --NRAVKRKNQQQNPQ-FAKRLKI--GNSKR-------------------LRNKKQTVQSK    255
RAG1-Zebrafish               211  FQRVGKKRTKPLKSAHILPKRFRRDSSESSRVWRQTTE-----------NPDGKEWLKLS    259
RAG1-Tetraodon               220  YQRTGRKRKKGLPRALSLAKRTRWDNSDGNAGGERRKQW----------RSALITWPKSR    269
RAG1-Stickleback             221  FQRTGRKRRKAIPRAQSLAKRARRDPGDCIVVGERKALRPFGDNRH---GPVLRAQGQLS    277
RAG1-Tilapia                 214  FQRTGRKRRKVIPRAQSLAKRTRWDCDAIAV-GERRVLRPFGDRH----GPAIRAWKKFS    268
RAG1-Platyfish               217  --KTGRKRRKVVPKVQSLAKRA-----------------------------SPVLRAWKKNG    247
RAG1L-Strongylocentrotus     109  STKRKAVGTDGLPPPK-IPSAAVSGTDEQQASCSFTAPSPPTARIYQ--PIKPQTRSDSR    165

                                  --------------------------------------------|------RING-------
                                  -------------------------------------|--------------ZDD--------
RAG1-Human                   257  ISSKDVMKKIANCSKIHLSTK--------LLAVDFPEHFVKSISCQICEHILADPVETNCK    309
RAG1-Opossum                 261  KSSKDLMKKLTNCSKMHLSTK--------CLAVDYPVDFVKAISCQICEHILTDPVETTCK    313
RAG1-Turkey                  254  INNKNVMKEITNCKNRHLSTK--------LLAVDYPADFIKSISCQICEHILADPVETTCR    306
RAG1-Turtle                  251  INSKNLMKKIANCKKIHLSTK--------LLAVDYPADFVKSISCQVCEHILADPVETTCS    303
RAG1-Lizard                  258  VNQKSLMKKIASCKKIHLSTK--------ILAVDYPSDFVKSISCQICEHILADPVETTCK    310
RAG1-Xenopus                 259  NSSKALNQMKDSCKKIHLDTN--------LLVVDYPSDFVKSMSCQVCEHILSDPVQTSCK    311
RAG1-Cod                     287  RPREHRVQGFTHCQRDHLGTR--------LLSEQLPVDLVRSVTCQVCHHLLSEPVQAPCR    339
RAG1-Coelacanth              256  SKKNALIKKIANCKKIHLSTK--------FLAAEYPVDFVKSMSCQICEHILADPVQSPCK    308
RAG1-Zebrafish               260  VQRGQWVKNITRCQRDHLSTK--------LIPTEVPADLIRAVTCQVCDHLLSDPVQSPCR    312
RAG1-Tetraodon               270  AQREQWVKSLTHCQKDHLSIK--------LISEKLPEDFLFCFTCVVCDHLLSDPVQPPCG    322
RAG1-Stickleback             278  IQREHWVKNITHCQRDHLNTK--------LICEKLPVDFLYSFTCLVCDHLLSDPVQSPCR    330
RAG1-Tilapia                 269  IQREQWVRNITRCQKDHLSTK--------LLSEKLPVDFVASFTCLVCDHLLSDPVQSTCG    321
RAG1-Platyfish               248  IQREQWVKNITHCQNDHLNTK--------LIAEKLPTDFIISFTCLVCDHLLCDPVQSPCG    300
RAG1L-Strongylocentrotus     166  NAEGPTYHLCSVCAATTLTKEKAVGTDDLPPPEIPSAAVSGLDEQQASCSFTAPLPPTAT    225
```

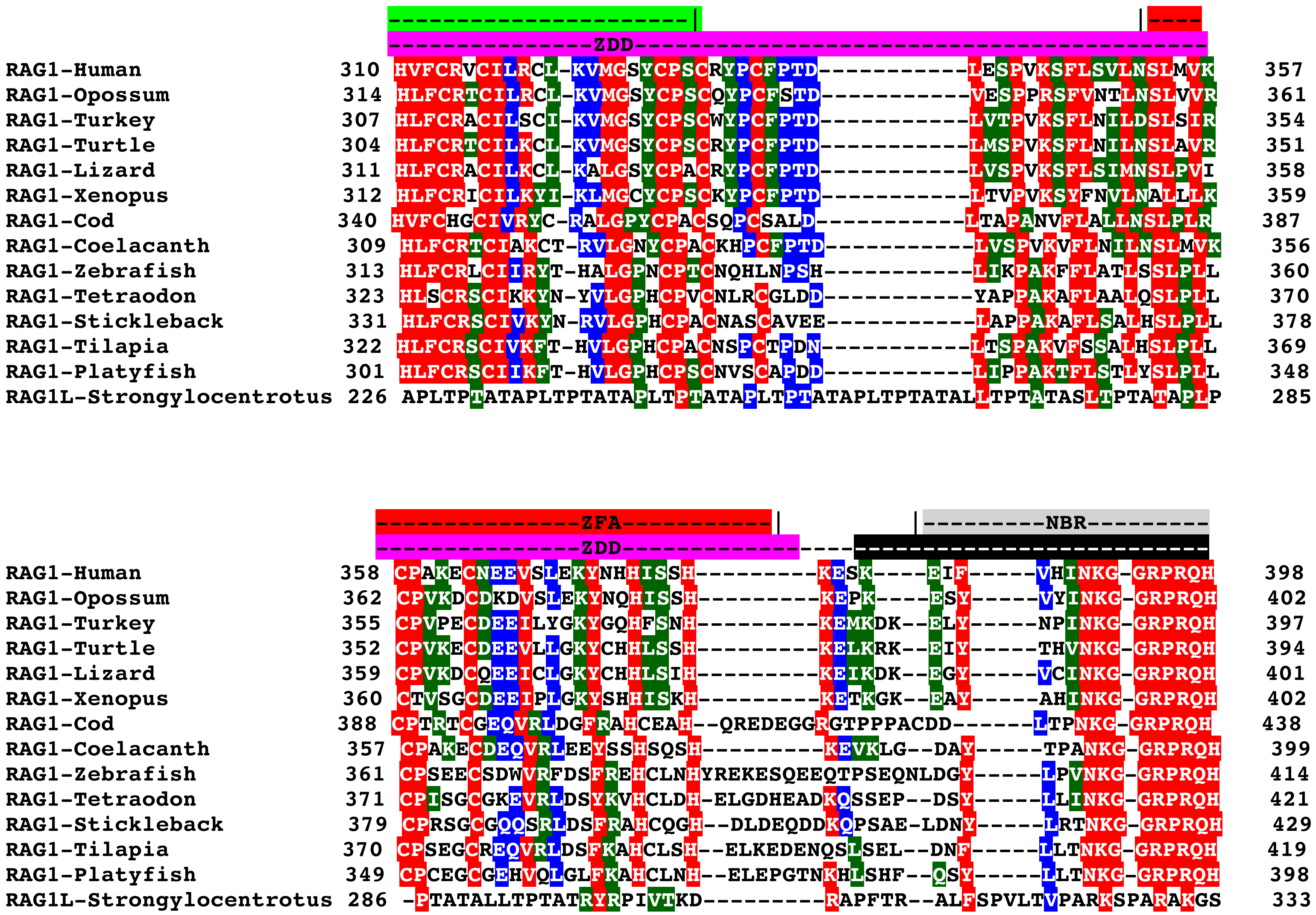

ZDD
RAG1-Human 310 HVFCRVCILRCL-KVMGSYCPSCRYPCFPTD------------LESPVKSFLSVLNSLMVK 357
RAG1-Opossum 314 HLFCRTCILRCL-KVMGSYCPSCQYPCFSTD------------VESPPRSFVNTLNSLVVR 361
RAG1-Turkey 307 HLFCRACILSCI-KVMGSYCPSCWYPCFPTD------------LVTPVKSFLNILDSLSIR 354
RAG1-Turtle 304 HLFCRTCILKCL-KVMGSYCPSCRYPCFPTD------------LMSPVKSFLNILNSLAVR 351
RAG1-Lizard 311 HLFCRACILKCL-KALGSYCPACRYPCFPTD------------LVSPVKSFLSIMNSLPVI 358
RAG1-Xenopus 312 HLFCRICILKYI-KLMGCYCPSCKYPCFPTD------------LTVPVKSYFNVLNALLLK 359
RAG1-Cod 340 HVFCHGCIVRYC-RALGPYCPACSQPCSALD------------LTAPANVFLALLNSLPLR 387
RAG1-Coelacanth 309 HLFCRTCIAKCT-RVLGNYCPACKHPCFPTD------------LVSPVKVFLNILNSLMVK 356
RAG1-Zebrafish 313 HLFCRLCIIRYT-HALGPNCPTCNQHLNPSH------------LIKPAKFFLATLSSLPLL 360
RAG1-Tetraodon 323 HLSCRSCIKKYN-YVLGPHCPVCNLRCGLDD------------YAPPAKAFLAALQSLPLL 370
RAG1-Stickleback 331 HLFCRSCIVKYN-RVLGPHCPACNASCAVEE------------LAPPAKAFLSALHSLPLL 378
RAG1-Tilapia 322 HLFCRSCIVKFT-HVLGPHCPACNSPCTPDN------------LTSPAKVFSSALHSLPLL 369
RAG1-Platyfish 301 HLFCRSCIIKFT-HVLGPHCPSCNVSCAPDD------------LIPPAKTFLSTLYSLPLL 348
RAG1L-Strongylocentrotus 226 APLTPTATAPLTPTATAPLTPTATAPLTPTATAPLTPTATALLTPTATASLTPTATAPLP 285
ZFA
ZDD
NBR
RAG1-Human 358 CPAKECNEEVSLEKYNHHISSH----------KESK----EIF-----VHINKG-GRPRQH 398
RAG1-Opossum 362 CPVKDCDKDVSLEKYNQHISSH----------KEPK----ESY-----VYINKG-GRPRQH 402
RAG1-Turkey 355 CPVPECDEEILYGKYGQHFSNH----------KEMKDK--ELY-----NPINKG-GRPRQH 397
RAG1-Turtle 352 CPVKECDEEVLLGKYCHHLSSH----------KELKRK--EIY-----THVNKG-GRPRQH 394
RAG1-Lizard 359 CPVKDCQEEICLGKYCHHLSIH----------KEIKDK--EGY-----VCINKG-GRPRQH 401
RAG1-Xenopus 360 CTVSGCDEEIPLGKYSHHISKH----------KETKGK--EAY-----AHINKG-GRPRQH 402
RAG1-Cod 388 CPTRTCGEQVRLDGFRAHCEAH--QREDEGGRGTPPPACDD------LTPNKG-GRPRQH 438
RAG1-Coelacanth 357 CPAKECDEQVRLEEYSSHSQSH----------KEVKLG--DAY-----TPANKG-GRPRQH 399
RAG1-Zebrafish 361 CPSEECSDWVRFDSFREHCLNHYREKESQEEQTPSEQNLDGY-----LPVNKG-GRPRQH 414
RAG1-Tetraodon 371 CPISGCGKEVRLDSYKVHCLDH-ELGDHEADKQSSEP--DSY-----LLINKG-GRPRQH 421
RAG1-Stickleback 379 CPRSGCGQQSRLDSFRAHCQGH--DLDEQDDKQPSAE-LDNY-----LRTNKG-GRPRQH 429
RAG1-Tilapia 370 CPSEGCREQVRLDSFKAHCLSH--ELKEDENQSLSEL--DNF-----LLTNKG-GRPRQH 419
RAG1-Platyfish 349 CPCEGCGEHVQLGLFKAHCLNH--ELEPGTNKHLSHF--QSY-----LLTNKG-GRPRQH 398
RAG1L-Strongylocentrotus 286 -PTATALLTPTATRYRPIVTKD----------RAPFTR--ALFSPVLTVPARKSPARAKGS 333

```
                                ---------------------------NBR--------------------------------
                                -----------------------Core RAG1-----------------------------
RAG1-Human                  399 LLSLTRRAQKHRLRELKLQVKAFADKEEGGDVKSVCMTLFLLALRARNEHRQADELEAIM 458
RAG1-Opossum                403 LLSLTRRAQKHRLRELKLQVKAFADKEEGGDLKAVCLTLFLLVLRARNEHRQADELEAIM 462
RAG1-Turkey                 398 LLSLTRRAQKHRLRELKRQVKAFAEKEEGGDIKAVCMTLFLLALRAKNEHKQADELEAIM 457
RAG1-Turtle                 395 LLSLTRRAQKHRLRELKLQVKAFAEKEEGGDIKAVCLTLFLLALRARNEHRQADELEAMM 454
RAG1-Lizard                 402 LLSLTRRAQKHRLRELKLQVKAFAEKEEGGDVKSVCLTLFLLALRARNEHRQADELEAIM 461
RAG1-Xenopus                403 LLTLTRRAQKHRLRELKMQVKAFADKEEEGDVKSVCLTLFLLALRARNEHRQADELEAIM 462
RAG1-Cod                    439 LLSLTRRAQKHRLRGLRCEVNQFAEKEEGGDVKAVCLTLFLLALRSNNEHRKADELEAIM 498
RAG1-Coelacanth             400 LLSLTRRAQKHRLRELKLQVRAFADKEEGGDVKSVCLTLFLLALRARNEHRQADELEAVM 459
RAG1-Zebrafish              415 LLSLTRRAQKHRLRDLKNQVKTFAEKEEGGDVKSVCLTLFLLALRAGNEHKQADELEAMM 474
RAG1-Tetraodon              422 LLSLTRRAQKHRLKDLKNHVKTFADKEEGGDLKSVCLTLFLLALRSGNEHRQADELEAIM 481
RAG1-Stickleback            430 LLSLTRRAQKHRLRDMKNQLKVFADKEEGGDLRSVCQTLFLLALRSGNEHRRADELEAMM 489
RAG1-Tilapia                420 LLSLTRRAQKHRLRDLKSQVKVFADKEEGGDLKSVCLTLFLLALRSGNEHRQADELEAMM 479
RAG1-Platyfish              399 LLSLTRRAQKHRLKDLKSQVKVFADKEEGGDLKSVCLTLFLLALRSGNEHRQADELEAMM 458
RAG1L-Strongylocentrotus    334 LHYVRRDCAKNRARGALDFMTSHSAAKNENETD-----LWFFGLHNRLRNEEDERAKMVM 388

                                -----------NBR------------------|------------------------------
                                --------------------------------------------------------------
RAG1-Human                  459 Q-----GKGSGLQPAVCLAIRVNTFLSCSQYHKMYRTVKAITGRQIFQPLHALRNAEKVL 513
RAG1-Opossum                463 Q-----GRGSGLQAAVCLAIRVNTFLSCSQYHKMYRTVKAITGRQIFQPLHALRNAEKTL 517
RAG1-Turkey                 458 Q-----GKGSGLHPAVCLAIRINTFLSCSQYHKMYRTVKAVTGRQIFQPLHALRTAEKAL 512
RAG1-Turtle                 455 Q-----GKGSGLHPAVCLAIRVNTFLSCSQYHKMYRTVKAITGRQIFQPLHALRTAEKAL 509
RAG1-Lizard                 462 Q-----GKGSGLHPAVCLAIRVNTFLSCSQYHKMYRTVKAVTGRQIFQPLHALRTAEKAL 516
RAG1-Xenopus                463 E-----GRGAGLHPAVCLAIRVNTFLSCSQYHKMYRTVKATTGRQIFQPLHALRNAEKAL 517
RAG1-Cod                    499 Q-----ARCFGLNAAVCLAIRVNTFLSCSQYHKMYRTVKATSGRQIFQPLHALRAAEKEL 553
RAG1-Coelacanth             460 Q-----GRGSALHPAVCLAIRVNTFLSCSQYHKMYRTVKATTGKQIFQPLHSLRNAEKAL 514
RAG1-Zebrafish              475 Q-----GRGFGLHPAVCLAIRVNTFLSCSQYHKMYRTVKATSGRQIFQPLHTLRNAEKEL 529
RAG1-Tetraodon              482 Q-----GRGSGLHPAVCLAIRVNTFLSCSQYHKMYRTVKATSGRQIFQPLHSLRAVEKEL 536
RAG1-Stickleback            490 Q-----GRGFELHPAVCLAIRVNTFLSCSQYHKMYRTVKATSGRQIFQPLNTLRAAEKGL 544
RAG1-Tilapia                480 Q-----GRGFGLSPAVCLAIRVNTFLSCSQYHKMYRTVKATSGRQIFQPLHTLRAAEKEL 534
RAG1-Platyfish              459 Q-----GRGFGLSPAVCLAIRVNTFLSCSQYHKMYRTVKATSGRQIFQPLHTLRAAEKEL 513
RAG1L-Strongylocentrotus    389 ELWTERKKSTDLSVDDCLAMRVGTLCTKGMYAEKYSFLKS-KGDKTFRPPGQLTKRESCY 447
```

```
                                 -------------------------------------------------------------------
                                 -------------------------- Core RAG1-------------------------------
RAG1-Human                  514 LPGYHHFE---------WQPPLKNVSSSTDVGIIDGLS-GLSSSVDDYPV-DTIAKRFRYD    563
RAG1-Opossum                518 LPGHHPFE---------WKPTLKNVSARTDVGIMDGLS-GLSASVDDYPV-DTIAKRFRYD    567
RAG1-Turkey                 513 LPGYHPFE---------WKPPLKNVSTNTEVGIIDGLS-GLPLSIDDYPI-DTIAKRFRYD    562
RAG1-Turtle                 510 LPGYHPFE---------WKPPLKNVSTNTEVGIIDGLS-GLPLSVDDYPV-DTIAKRFRYD    559
RAG1-Lizard                 517 LPGYHQFE---------WKPPLKNVSSNTEVGIIDGLS-GIQHLVDDYPV-DTIAKRFRYD    566
RAG1-Xenopus                518 LPGYHSFE---------WRPPLKNVSTRTDVGIIDGLS-GLNRSVDEYPV-DTISKRFRYD    567
RAG1-Cod                    554 LPGFHAFE---------WQPALASVSPSCHVGIIDGLS-GWAASVDDAPA-DTITRRFRYD    603
RAG1-Coelacanth             515 LPGFHPFE---------WQPPLKNVSSTTEVGIIDGMS-GMTQFVDEYPL-DTISKRFRYD    564
RAG1-Zebrafish              530 LPGFHQFE---------WQPALKNVSTSWDVGIIDGLS-GWTVSVDDVPA-DTISRRFRYD    579
RAG1-Tetraodon              537 LPGFHQFE---------WQPPLRNVSTSCSVGIINGLS-GWTSSVDDSPA-DTITRRFRYD    586
RAG1-Stickleback            545 LPGFHQFE---------WQPALKNVSTSCTVGIINGLS-GWASSVDDFPA-DTTTRRFRYD    594
RAG1-Tilapia                535 LPGFHQFE---------WQPALKNVSPSCNVGIINGLS-GWSSSVDDVPA-DTITRRFRYD    584
RAG1-Platyfish              514 LPGFHQFE---------WQPALKNVSTSYNVGIINGLS-GWTSSLDDSPA-DTITRRFRYD    563
RAG1L-Strongylocentrotus    448 MPGNVRFGLMEGGKCVYHTPEKSLEEFDDHSMYEPIRINVRSKLTEFALPNCIGVAWSYP     507


                                 -----------------Central domain----------------------------
                                 -----------------------------------------------------------
RAG1-Human                  564 SALVSALMDMEEDILEGMRSQDLDDYL-NGPFTVV---VKESCDGMGDVS-EKHGSGPVV    618
RAG1-Opossum                568 VALVSALMDMEEDILEGMKSKDLSDYL-NGPFTVV---VKESCDGMGDVS-EKHGSGPAV    622
RAG1-Turkey                 563 TALVSALKDMEEEILEGMKAKNLDDYL-NGPFTVV---VKECCDGMGDVS-EKHGSGPAV    617
RAG1-Turtle                 560 AALVSALMDMEEDILEGMKAKDLDDYL-NGPFTVV---VKESCDGMGDVS-EKHGSGPAV    614
RAG1-Lizard                 567 AALASALMDMEEDILEGLKRQDLDDYF-KGPFTVV---IKESCDGMGDVS-EKHGCGPAV    621
RAG1-Xenopus                568 AALVSALKDMEEDILEGLKTQGLDDYM-SGPFTVV---VKESCDGMGDVS-EKHGSGPAV    622
RAG1-Cod                    604 VALASALKDLEEDIMDGLRQQGLEDSACTEGFSVM---IKESCDGMGDVS-EKHGGGPAV    659
RAG1-Coelacanth             565 AALVSALKDLEEELLKGLIEEDLEDYL-SGPFTVI---IKESCDGMGDVS-EKHGSGPAV    619
RAG1-Zebrafish              580 VALVSALKDLEEDIMEGLRERALDDSMCTSGFTVV---VKESCDGMGDVS-EKHGSGPAV    635
RAG1-Tetraodon              587 VAIVSALKDLEEDIMEGLRDNGLEDSTCTLGFSIL---IKESCDGMGDVS-EKHGGGPLV    642
RAG1-Stickleback            595 VALVSALKDLEEDILEGLRESGMEDSACTSGFSVM---IKESCDGMGDVS-EKHGGGPPV    650
RAG1-Tilapia                585 VALVSALKDLEEDIMEGLRESGMEDSACTSGFSVM---IKECCDGMGDVS-EKHGGGPVV    640
RAG1-Platyfish              564 VALVGALKDLEEDIMEGLRDTGMEDSACTLGFRVM---IKECCDGMGDVS-EKHGGGPAV    619
RAG1L-Strongylocentrotus    508 EAVAKTLEELDENIREGMLKVGLNP---DGPSIIIDTTLKDGADGMGEIAVHKMKSDTFL    564
```

```
RAG1-Human                      619 PEKAVRFSFTIMKITIAHSSQ---------NVKVFEEAKPNSELCCKPLCLMLADESDHET 670
RAG1-Opossum                    623 PEKAVRFSFTIMNISIAQGQE---------SVRIFEEAKPNSELCCKPLCLMLADESDHET 674
RAG1-Turkey                     618 PEKAVRFSFTVMNIAIDHGNE---------RIRIFEEVKPNSELCCKPLCLMLADESDHET 669
RAG1-Turtle                     615 PEKAVRFSFTLMSISIAHGNE---------NVRVFEEVKPNSELCCKPLCLMLADESDHET 666
RAG1-Lizard                     622 PEKAVRFSFTLMTISVTHGNA---------SIRVFEECKPNSELCCKPLCLMLADESDHET 673
RAG1-Xenopus                    623 PEKAVRFSFTVMNISVANNSG---------QVRIFEETKPNSELCCKPLCLMLADESDHET 674
RAG1-Cod                        660 PEKAVRFSFTVMSVSLRVADDGEEAGNAEEVIIFQEPKPNSELSCKPLCLMFVDESDHET 719
RAG1-Coelacanth                 620 PEKAVRYSFTIMTISVANSHN--------ENVTIFEEGKPNSELCCKPLCLMLADESDHET 672
RAG1-Zebrafish                  636 PEKAVRFSFTIMSISIRLEGE-------DDGITIFQEQKPNSELSCRPLCLMFVDESDHET 689
RAG1-Tetraodon                  643 PEKAVRFSFTIMSVSVKAGGE--------KDKVIFTEPKPNSELSCKPLSLMFVDESDHET 695
RAG1-Stickleback                651 PEKAVRFSFTVMSVSVLADEE-------VEEVTIFTEAKPNSELSCKPLCLMFVDESNHET 704
RAG1-Tilapia                    641 PEKAVRFSFTVMSVSVLADDE-------EEEVAIFTEPKPNSELSCKPLCLMFVDESDHET 694
RAG1-Platyfish                  620 PEKAVRFSFTVMSVSIQAEDE-------QEEVTIFTEPKPNSELSCKPLCLMFVDESDHET 673
RAG1L-Strongylocentrotus        565 PDKAFRASFVVLKCEVKRDDG--------TRDLVFEEPKPNSVIVNRPLLEAIGDENSAST 617
```

```
                                                                                  ----ZFB---
RAG1-Human                      671 LTAILSPLIAEREAMKSSELMLELGGILRTFKFIFRGTGYDEKLVREVEGLEASGSVYIC 730
RAG1-Opossum                    675 LTAILSPLIAEREAMKSSELLLEMGGIQRTFKFIFRGTGYDEKLVREVEGLEASGSVYIC 734
RAG1-Turkey                     670 LTAILSPLIAEREAMKNSELLLEMGGILRTFKFIFRGTGYDEKLVREVEGLEASGSTYIC 729
RAG1-Turtle                     667 LTAILSPLIAERETMKTSVLLLEMGGILRTFKFIFRGTGYDEKLVREVEGLEASGSTYIC 726
RAG1-Lizard                     674 LTAILSPLVAEREAMKDSVLILDMAGIPRTFKFIFRGTGYDEKLVREVEGLEASGSTYIC 733
RAG1-Xenopus                    675 LTAILCPLIAEREAMKTAELLLEIGGILRSFKFMFRGTGYDEKLVREVEGLEASGSIYIC 734
RAG1-Cod                        720 LTAVLGPVSAERSAIKRSRLILSIGGLSRLFSFRFRGSGYDEKMVRDVEGMEASGSTYVC 779
RAG1-Coelacanth                 673 LTAILGPVIAEREAMKNSELFLEMGGILRSFKFIFRGTGYDEKLIRDVEGLEASGSSYIC 732
RAG1-Zebrafish                  690 LTAILGPVVAERKAMMESRLIISVGGLLRSFRFFFRGTGYDEKMVREMEGLEASGSTYIC 749
RAG1-Tetraodon                  696 LTAIMSPIIAERDAMKNSRLIVSIGGLPRSFRIHFRGTGYDEKMVREMEGLEAAGSTYIC 755
RAG1-Stickleback                705 LTAVLGPIVAERNAMKESRLILSVGGLPRSFRFHFRGTGYDEKMVREVEGLEASGSTYVC 764
RAG1-Tilapia                    695 LTGVLAPIVAERNAMKESRLILSMGGLPRSFRFHFRGTGYDEKMVREMEGLEASGSTYIC 754
RAG1-Platyfish                  674 LTAVLGPIVAERNAMKESRLILSMGGMPRSFRFHFRGTGYDEKMVREMEGLEASGSTYIC 733
RAG1L-Strongylocentrotus        618 SAVLMRKMEKERLILQNSIMTIHAGTYTRLHRFTIYNSMIDEKLARSSGGLQGSGSNFIC 677
```

```
                           ------------ZFB---------------

RAG1-Human                731 TLCDATRLEASQNLVFHSITRSHAENLERYEVWRSNPYHESVEELRDRVKGVSAKPFIET 790
RAG1-Opossum              735 TLCDATRLEASQNLVFHSITRSHAENLERYEVWRSNPYQESAEELRDRVKGVSAKPFIET 794
RAG1-Turkey               730 TLCDATRLEASQNLVFHSITRSHAENLERYEIWRSNPYHESVDELRDRVKGVSAKPFIET 789
RAG1-Turtle               727 TLCDATRLEASQNLVLHSITRSHAENLERYEVWRSNPYHESVDELRDRVKGVSAKPFIET 786
RAG1-Lizard               734 TLCDATRLEASQNLILHSITRSHAENLERYELWRTNPYHETVDELRDRVKGVSAKPFIET 793
RAG1-Xenopus              735 TLCDSTRLEASQNLVNHSITRSHGENLQRYETWRSNPHHESVDELRDRVKGVSAKPFIET 794
RAG1-Cod                  780 TLCDSTRADAARNMVLHSVTRSHQENLERYETWRTNPFSESADELRDRVKGISAKPFLET 839
RAG1-Coelacanth           733 TLCDSTRSEASQNFILHSITRSHKENLERYEIWRSNPYQEPVEELRDRVKGVSAKPFIET 792
RAG1-Zebrafish            750 TLCDSTRAEASQNMVLHSITRSHDENLERYEIWRKNPFSESADELRDRVKGVSAKPFMET 809
RAG1-Tetraodon            756 TLCDSSRVEASENMVLHSITRSHNENLERYEIWRTNPFSESVDELRDRVKGVSAKAFMET 815
RAG1-Stickleback          765 TLCDSTRAEASKNMVLHSITRSHEENLDRYEIWRTNPFSESVEELRDRVKGISAKPFMET 824
RAG1-Tilapia              755 TLCDSSRAEASQNMVLHSITRSHEENLERYEIWRTNPFSESVDELRDRVKGVSAKPFLET 814
RAG1-Platyfish            734 TLCDSSRAEAAQNMVLHSVTRSHDENLERYEIWRTNPFSESVDELRDRVKGVSAKPFLET 793
RAG1L-Strongylocentrotus  678 TLCHATKTSAKTQLGSFKIDRTLTETQQTLTYITTNPDNLTPDELTTEAGGVKRKPLLTS 737

RAG1-Human                791 VPS---IDALHCDIGNAAEFYKIFQLEIGEVYKNPNASKEERKRWQATLDKHL----RKK 843
RAG1-Opossum              795 VPS---IDALHCDIGNAAEFYKLFQMEIGEVYKNPNASKEERKRWQATLDKHL----RKK 847
RAG1-Turkey               790 VPS---IDALHCDIGNATEFYRIFQMEIGEVYKNPDASKEERKRWQLTLDKHL----RKK 842
RAG1-Turtle               787 VPS---IDALHCDIGNAAEFYKIFQFEIGEVYKNPNASKEERKRWQSTLDKHL----RKK 839
RAG1-Lizard               794 VPS---IDALHCDIGNAAEFYKIFQFEIGEVYKNTDASKEERRRWQSTLDKHL----RKK 846
RAG1-Xenopus              795 LPS---IDALHCDIGNAAEFYRIFQLEIGEFYKNFSATKEEKKRWQATLDKHL----RKR 847
RAG1-Cod                  840 HPT---LDALHCDIGNATEFYKIFQDEIGEVFQKTNPSREERRSWRAALDKQL----RKT 892
RAG1-Coelacanth           793 LPS---IDALHCDIGNATEFYKIFQDEIGEIYKNPNPSREEKKRWHSVLDKHL----RKN 845
RAG1-Zebrafish            810 QPT---LDALHCDIGNATEFYKIFQDEIGEVYQKPNPSREERRRWRSTLDKQL----RKK 862
RAG1-Tetraodon            816 HPT---LDALHCEIGIATEFYKIFQDEIGEVYQKVNPSREERRGWRAALDKQL----RKS 868
RAG1-Stickleback          825 QPT---MDALHCDIGNATEFYKIFQDEIGEVYRKVNPSREERRSWRAALDKQL----RMK 877
RAG1-Tilapia              815 QPT---LDALHCDIGNATEFYKIFQDEIGEVYKKANPSREERRSWRAALDKQL----RKK 867
RAG1-Platyfish            794 QPT---IDALHCDIGNATEFYKIFQDEIGEVYKNPNPSREDRRSWRAALDKQL----RKK 846
RAG1L-Strongylocentrotus  738 EPKQQLMDATHADIDLGQFFKKIIVREMAGVHKW--EASENVKQYIVDAERRLNINVREL 795
```

```
                                 -----------------C-termenal domain------------------------
                                 ----------------------------------------------------------
RAG1-Human                   844 MNLKPIMRMNGNFARKLMTKETVDAVCELIPSEERHEALRELMDLYLKMKPVWRSSCPAK  903
RAG1-Opossum                 848 MNLKPIMRMNGNFARRLMTKETVEAVCELIHCEERHEALRELMDLYLKMKPVWRSTCPAK  907
RAG1-Turkey                  843 MKLKPMMRMSGNFARKLMSKETVEAVCELIKCEERHEALKELMDLYLKMKPVWRSSCPAK  902
RAG1-Turtle                  840 MNLKPIMRMNGNFARKLMTKETVEAICELIKCEERHEALRELMDLYLKMKPVWRSSCPAK  899
RAG1-Lizard                  847 MNLKPMTRMNGNFARKLMTKETVEAVCELIKSEERHEALRELMDLYLKMKPVWRSSCPTK  906
RAG1-Xenopus                 848 MNLKPIMRMNGNFARKLMSKETVEAVCELVACEERQTALTELMDLYLKMKPVWRSSCPAK  907
RAG1-Cod                     893 MKLKPVMRMNGNYARKLMSVEAVEAVCELVPSESRRQALRQLMEIYLEMKPVWRATSPAT  952
RAG1-Coelacanth              846 MNLKPVMRMNGNYARKLMTKETVNAVCELIPSEERQEALKELVDLYLKMKPVWRSTCPAK  905
RAG1-Zebrafish               863 MKLKPVMRMNGNYARRLMTREAVEAVCELVPSAERREALLKLMDLYLQMKPVWRSTCPSR  922
RAG1-Tetraodon               869 MKLKPVMRMNGNYARRLMSLEAVELVCELVPTEERREALRELMTLYLQMKPVWRATCPAK  928
RAG1-Stickleback             878 MKLKPVMRMNGNYARRLMTQEVVEVICELVPSEERREALRELMRLYIQMKPVWRATCPAK  937
RAG1-Tilapia                 868 MKLKPVMRMNGNYARRLMTQEAVDVVCELVPSEERREALRELMKLYLQMKPVWRATCPAK  927
RAG1-Platyfish               847 MKLKPVMRMNGNYARRLMTMEATEVVCELVPSEERREVLRELMRLYLQMKPVWRATCPAK  906
RAG1L-Strongylocentrotus     796 LGTAPSLMMPGNYARALFKEKNEDVFLELIRNEERKELLRSVLQKFRALRKIYREHHPNK  855


                                 ----------------------------------------------------------
                                 ----------------------------------------------------------
RAG1-Human                   904 ECPESLCQYSFNSQRFAELLSTKFKYRYEGKITNYFHKTLAHVPEII---ERDGSIGAWA  960
RAG1-Opossum                 908 ECPESLCQYSFNSQRFAELLSTKFQYRYEGKITNYFHKTLAHVPEII---ERDGSIGAWA  964
RAG1-Turkey                  903 ECPELLCQYSYNSQRFAELLSTKFKYRYEGKITNYFHKTLAHVPEII---ERDGSIGAWA  959
RAG1-Turtle                  900 ECPELLCQYSFNSQRFAELLSTKFKYRYEGKITNYFHKTLAHVPEII---ERDGSIGAWA  956
RAG1-Lizard                  907 ECPELVCQYSFNSQRFAELLATKFRYRYAGKITNYFHKTLAHVPEII---ERDGSIGAWA  963
RAG1-Xenopus                 908 ECPELLCQYSFHSQRFAELLSTKFKYRYEGKITNYFHKTLAHVPEII---ERDGSIGAWA  964
RAG1-Cod                     953 ECPDQLCRYSFNSQRFADLLASTFSYRYNGKITNYLHKTLAHVPEII---ERDGSIGAWA 1009
RAG1-Coelacanth              906 ECPELLCQYSFHSQRFAELLSTMYRYRYEGKITNYLHKTLAHVPEII---ERDGSIGAWA  962
RAG1-Zebrafish               923 DCPDQLCQYSYNSQQFADLLSSMFKYRYDGKITNYLHKTLAHVPEIV---ERDGSIGAWA  979
RAG1-Tetraodon               929 ECPDQLCRYSFNSQRFAEVLSSTFKYRYNKKITNYLHKTLAHVPEII---ERDGSIGAWA  985
RAG1-Stickleback             938 ECPDQLCSYSFNSQRFADLLSSTFKYRYNGKITNYLHKTLAHVPEII---ERDGSIGAWA  994
RAG1-Tilapia                 928 ECPDQLCRYSFNSQRFADLLSTTFKYRYNGKITNYLHKTLAHVPEII---ERDGSIGAWA  984
RAG1-Platyfish               907 ECPDQLCRYSFNSQRFADLLSATFKYRYNGKITNYLHKTLAHVPEII---ERDGSIGAWA  963
RAG1L-Strongylocentrotus     856 REVQG---FKKKAVQIGRELLEHFEYVC---WPNYLHKIFEHTQEAMLSEDGPGSIGILS  909
```

```
                                   ------------------
                                   --------------------------C-terminal end---------------------
RAG1-Human                   961  SEGNESGNKLFRRFRKMNARQSKCYE-MEDVLKHHWLYTSKYLQKFMNAH-NALKTSGFT   1018
RAG1-Opossum                 965  SEGNESGNKLFRRFRKMNARQSKCYE-MEDVLKHHWLYTSKYLQKFMNAH-NALKNSGLI   1022
RAG1-Turkey                  960  SEGNESGNKLFRRFRKMNARQSKFYE-MEDVLKHHWLYTSKFLQKFMNAH-KVLRSQGFV   1017
RAG1-Turtle                  957  SEGNESGNKLFRRFRKMNARQSKCYE-MEDVLKHHWLYTSKYLQKFMNAH-NTLKGQGFT   1014
RAG1-Lizard                  964  SEGNESGNKLFRRFRKMNARQSKFYE-MEDVLKHHWLYTSKHLQKFMNAH-NTLKSQGFT   1021
RAG1-Xenopus                 965  SEGNESGNKLFRRFRKMNARQSKVYE-MEDVLKHHWLYTSKHLQKFMNAH-INLKNQGFT   1022
RAG1-Cod                    1010  SEGNESANKLFRRFRKMNARQSKVFE-LEDVLKHHWLYTSKYLQKYMEAHKDSAKVLNAC   1068
RAG1-Coelacanth              963  SEGNESGNKLFRRFRKMNARQSKYYE-LEDVLKHHWLYTSKYLQKFMNAH-KALKGEMFI   1020
RAG1-Zebrafish               980  SEGNESGNKLFRRFRKMNARQSKTFE-LEDILKHHWLYTSKYLQKFMEAHKNSVKAMQAT   1038
RAG1-Tetraodon               986  SEGNESANKLFRRFRKMNARQSKSFE-LEDVLKHHWLYTSKYLQKFMEAHKDSAKALQAS   1044
RAG1-Stickleback             995  SEGNESANKLFRRFRKMNARQSKAFE-LEDVLKHHWLYTSKYLQKFMEAHKDSAKALQAT   1053
RAG1-Tilapia                 985  SEGNESANKLFRRFRKMNARQSKTFE-LEDVLKHHWLYTSKYLQKFMEAHKDSAKALQAT   1043
RAG1-Platyfish               964  SEGNESANKLFRRFRKMNARQSKAFE-LEDVLKHHWLYTSKYLQKFMEAH----------   1012
RAG1L-Strongylocentrotus     910  SEGSEAANKLFRKLRNNFSRRGDVLDGLRDILWFHWLYTSPKLVRLRAVTRGTYTCSRCG    969


                                   -------------------------
RAG1-Human                  1019  MNPQASLGDPLGIEDSLESQDSMEF   1043
RAG1-Opossum                1023  INPQADFEDSLGLDDSQEILDSMEF   1047
RAG1-Turkey                 1018  IDPDDGLGDSSPPEISLESSDSVEL   1042
RAG1-Turtle                 1015  IDPGQSLEDSLALEGTLDITDSMEF   1039
RAG1-Lizard                 1022  ISPEEGGGDFITLDESLEIPDSMEF   1046
RAG1-Xenopus                1023  VD-LDNPDQMERLESSVESLESVEF   1046
RAG1-Cod                    1069  FEPAVAEGD----VDM--CLDAPGF   1087
RAG1-Coelacanth             1021  KGPEDIPEEPFDFEDSLGTFDSMEF   1045
RAG1-Zebrafish              1039  INPEETPEE----ADN--SLDVPDF   1057
RAG1-Tetraodon              1045  IDPVLSKDD----EDM----TEIDF   1061
RAG1-Stickleback            1054  IDPEASQDC----ENVFSSLEVNDF   1074
RAG1-Tilapia                1044  IDPVEIQDD----DEM--SLEVPDF   1062
RAG1-Platyfish                 -  -------------------------      -
RAG1L-Strongylocentrotus     970  AEGHNKKSCNVKAT-----------    983
```
