# Supplementary material for: Data on the evolutionary history of the V(D)J recombination-activating protein 1 – RAG1 coupled with sequence and variant analyses
Source: Data Brief. 2016 May 20;8:87–92. doi: 10.1016/j.dib.2016.05.021 (PMC4887553; doi:10.1016/j.dib.2016.05.021)
Supplement: Supplementary file 2 — Supplementary material Table S1. Overview of 751 germline variants of RAG1 gene deduced from 1000 genome data. [file mmc2.pdf]

Table S1. Overview of 751 germline variants of RAG1 gene deduced from 1000 genome data.

Variant ID	Chromosomal location	Alleles	gmaf	Class	status	Variant types	Aachange	Aapos	sift	polyphen
rs4151025	11:3659515711:36595157	G/A	0.009 (A)	SNP	Multiple_observations,Frequency,HapMap,1000Genomes,ESP,	Synonymous variant	A	101	-	-
rs4151027	11:3659536011:36595360	C/T	0.001 (T)	SNP	Multiple_observations,Frequency,HapMap,1000Genomes,ESP,	Missense variant	S/L	169	31deleterious(0.03)	1benign(0)
rs4151029	11:3659559411:36595594	G/A	0.001 (A)	SNP	Multiple_observations,Frequency,HapMap,1000Genomes,ESP,	Missense variant	R/H	247	81tolerated(0.08)	26benign(0.025)
rs3740955	11:3659560011:36595600	A/G	0.409 (A)	SNP	Multiple_observations,Frequency,HapMap,1000Genomes,ESP,	Missense variant	H/R	249	371tolerated(0.37)	1benign(0)
rs4151031	11:3659620011:36596200	G/A	0.004 (A)	SNP	Multiple_observations,Frequency,HapMap,1000Genomes,ESP,	Missense variant	R/K	449	131tolerated(0.13)	393benign(0.392)
rs4151033	11:3659749211:36597492	G/A	0.013 (A)	SNP	Multiple_observations,Frequency,HapMap,1000Genomes,ESP,	Missense variant	E/K	880	71tolerated(0.07)	877possibly damaging(0.876)
rs4151034	11:3659751311:36597513	G/A	0.003 (A)	SNP	Multiple_observations,Frequency,HapMap,1000Genomes,ESP,	Missense variant	D/N	887	131tolerated(0.13)	20benign(0.019)
rs1980131	11:3659773411:36597734	A/G	0.070 (G)	SNP	Multiple_observations,Frequency,HapMap,1000Genomes,ESP,	Synonymous variant	A	960	-	-
rs4151030	11:3659576011:36595760	C/A	0.017 (A)	SNP	Multiple_observations,Frequency,HapMap,1000Genomes,Cited,ESP,	Missense variant	D/E	302	31deleterious(0.03)	705possibly damaging(0.704)
rs2227973	11:3659731311:36597313	A/G	0.246 (G)	SNP	Multiple_observations,Frequency,HapMap,1000Genomes,Cited,ESP,	Missense variant	K/R	820	121tolerated(0.12)	1benign(0)
rs7107464	11:3658494411:36584944	T/C	0.183 (C)	SNP	Multiple_observations,Frequency,HapMap,1000Genomes,	Upstream gene variant	-	-	-	-
rs1515060	11:3658632211:36586322	G/A	0.164 (A)	SNP	Multiple_observations,Frequency,HapMap,1000Genomes,	Upstream gene variant	-	-	-	-
rs3758873	11:3658701711:36587017	A/C	0.378 (C)	SNP	Multiple_observations,Frequency,HapMap,1000Genomes,	Upstream gene variant	-	-	-	-
rs12277745	11:3658718011:36587180	C/T	0.018 (T)	SNP	Multiple_observations,Frequency,HapMap,1000Genomes,	Upstream gene variant	-	-	-	-
rs4150997	11:3658892111:36588921	A/G	0.003 (G)	SNP	Multiple_observations,Frequency,HapMap,1000Genomes,	Upstream gene variant	-	-	-	-
rs4150998	11:3658894711:36588947	G/A	0.003 (A)	SNP	Multiple_observations,Frequency,HapMap,1000Genomes,	Upstream gene variant	-	-	-	-
rs2056094	11:3658907111:36589071	G/C	0.069 (C)	SNP	Multiple_observations,Frequency,HapMap,1000Genomes,	Upstream gene variant	-	-	-	-
rs4150999	11:3658919311:36589193	G/C	0.021 (C)	SNP	Multiple_observations,Frequency,HapMap,1000Genomes,	Upstream gene variant	-	-	-	-
rs4151000	11:3658934111:36589341	C/T	0.063 (T)	SNP	Multiple_observations,Frequency,HapMap,1000Genomes,	Upstream gene variant	-	-	-	-
rs872053	11:3658962211:36589622	A/G	0.069 (G)	SNP	Multiple_observations,Frequency,HapMap,1000Genomes,	5 prime UTR variant	-	-	-	-
rs4151001	11:3659011811:36590118	A/G	0.015 (G)	SNP	Multiple_observations,Frequency,HapMap,1000Genomes,	Intron variant	-	-	-	-
rs4151002	11:3659021311:36590213	G/A	0.125 (A)	SNP	Multiple_observations,Frequency,HapMap,1000Genomes,	Intron variant	-	-	-	-
rs4151005	11:3659145711:36591457	A/G	0.035 (G)	SNP	Multiple_observations,Frequency,HapMap,1000Genomes,	Intron variant	-	-	-	-
rs4151007	11:3659177611:36591776	T/C	0.002 (C)	SNP	Multiple_observations,Frequency,HapMap,1000Genomes,	Intron variant	-	-	-	-
rs4151012	11:3659301111:36593011	T/C	0.002 (C)	SNP	Multiple_observations,Frequency,HapMap,1000Genomes,	Intron variant	-	-	-	-
rs4151016	11:3659350611:36593506	T/C	0.003 (C)	SNP	Multiple_observations,Frequency,HapMap,1000Genomes,	Intron variant	-	-	-	-
rs4151017	11:3659354311:36593543	G/A	0.005 (A)	SNP	Multiple_observations,Frequency,HapMap,1000Genomes,	Intron variant	-	-	-	-
rs4151018	11:3659369111:36593691	A/G	0.005 (G)	SNP	Multiple_observations,Frequency,HapMap,1000Genomes,	Intron variant	-	-	-	-
rs4151019	11:3659378811:36593788	C/T	0.003 (T)	SNP	Multiple_observations,Frequency,HapMap,1000Genomes,	Intron variant	-	-	-	-
rs4151023	11:3659441711:36594417	A/G	0.010 (G)	SNP	Multiple_observations,Frequency,HapMap,1000Genomes,	Intron variant	-	-	-	-
rs4151024	11:3659446011:36594460	T/G	0.016 (G)	SNP	Multiple_observations,Frequency,HapMap,1000Genomes,	Intron variant	-	-	-	-
rs4151032	11:3659642711:36596427	C/T	0.003 (T)	SNP	Multiple_observations,Frequency,HapMap,1000Genomes,	Missense variant	P/S	525	41deleterious(0.04)	975probably damaging(0.974)
rs4151036	11:3659838411:36598384	G/A	0.010 (A)	SNP	Multiple_observations,Frequency,HapMap,1000Genomes,	3 prime UTR variant	-	-	-	-
rs4151038	11:3659857511:36598575	G/A	0.010 (A)	SNP	Multiple_observations,Frequency,HapMap,1000Genomes,	3 prime UTR variant	-	-	-	-
rs4151040	11:3659906911:36599069	C/T	0.070 (T)	SNP	Multiple_observations,Frequency,HapMap,1000Genomes,	3 prime UTR variant	-	-	-	-
rs4151041	11:3659909011:36599090	A/G	0.003 (G)	SNP	Multiple_observations,Frequency,HapMap,1000Genomes,	3 prime UTR variant	-	-	-	-
rs4151044	11:3659996411:36599964	C/T	0.016 (T)	SNP	Multiple_observations,Frequency,HapMap,1000Genomes,	3 prime UTR variant	-	-	-	-
rs4151045	11:3660023211:36600232	T/C	0.070 (C)	SNP	Multiple_observations,Frequency,HapMap,1000Genomes,	3 prime UTR variant	-	-	-	-
rs4151046	11:3660031511:36600315	G/T	0.003 (T)	SNP	Multiple_observations,Frequency,HapMap,1000Genomes,	3 prime UTR variant	-	-	-	-
rs4151047	11:3660051611:36600516	G/T	0.017 (T)	SNP	Multiple_observations,Frequency,HapMap,1000Genomes,	3 prime UTR variant	-	-	-	-
rs1056403	11:3660117011:36601170	G/A	0.412 (G)	SNP	Multiple_observations,Frequency,HapMap,1000Genomes,	3 prime UTR variant	-	-	-	-
rs2673017	11:3660131711:36601317	A/G	0.027 (G)	SNP	Multiple_observations,Frequency,HapMap,1000Genomes,	Downstream gene variant	-	-	-	-
rs7480202	11:3660513411:36605134	C/T	0.104 (T)	SNP	Multiple_observations,Frequency,HapMap,1000Genomes,	Downstream gene variant	-	-	-	-
rs16929067	11:3660573511:36605735	G/C	0.099 (C)	SNP	Multiple_observations,Frequency,HapMap,1000Genomes,	Downstream gene variant	-	-	-	-
rs11033698	11:3660609311:36606093	T/G	0.147 (G)	SNP	Multiple_observations,Frequency,HapMap,1000Genomes,	Downstream gene variant	-	-	-	-
rs12421641	11:3660621911:36606219	C/A	0.104 (A)	SNP	Multiple_observations,Frequency,HapMap,1000Genomes,	Downstream gene variant	-	-	-	-
rs138801620	11:3659491411:36594914	C/T	0.001 (T)	SNP	Multiple_observations,Frequency,ESP,	Synonymous variant	H	20	-	-
rs147486240	11:3659500611:36595006	A/T	-	SNP	Multiple_observations,Frequency,ESP,	Missense variant	D/V	51	301tolerated(0.3)	9benign(0.008)
rs143654819	11:3659504811:36595048	T/C	-	SNP	Multiple_observations,Frequency,ESP,	Missense variant	V/A	65	321tolerated(0.32)	1benign(0)
rs144430517	11:3659528611:36595286	G/T	-	SNP	Multiple_observations,Frequency,ESP,	Missense variant	K/N	144	21deleterious(0.02)	825possibly damaging(0.824)
rs149229197	11:3659538111:36595381	G/T	-	SNP	Multiple_observations,Frequency,ESP,	Missense variant	C/F	176	1deleterious(0)	867possibly damaging(0.866)
rs34841221	11:3659543111:36595431	G/A	-	SNP	Multiple_observations,Frequency,ESP,	Missense variant	E/K	193	61tolerated(0.06)	627possibly damaging(0.626)
rs141049427	11:3659600811:36596008	T/C	-	SNP	Multiple_observations,Frequency,ESP,	Missense variant	I/T	385	611tolerated(0.61)	1benign(0)
rs143289774	11:3659643211:36596432	G/A	0.001 (A)	SNP	Multiple_observations,Frequency,ESP,	Synonymous variant	L	526	-	-
rs142333735	11:3659658111:36596581	A/T	-	SNP	Multiple_observations,Frequency,ESP,	Missense variant	D/V	576	1deleterious(0)	989probably damaging(0.988)
rs139863630	11:3659686311:36596863	C/T	-	SNP	Multiple_observations,Frequency,ESP,	Missense variant	T/M	670	71tolerated(0.07)	986probably damaging(0.985)
rs143227621	11:3659688911:36596889	A/G	0.000 (G)	SNP	Multiple_observations,Frequency,ESP,	Missense variant	I/V	679	1001tolerated(1)	523possibly damaging(0.522)
rs61758791	11:3659730511:36597305	A/G	-	SNP	Multiple_observations,Frequency,ESP,	Synonymous variant	E	817	-	-
rs147656090	11:3659732211:36597322	A/C	-	SNP	Multiple_observations,Frequency,ESP,	Missense variant	N/T	823	11deleterious(0.01)	5benign(0.004)

rs145772007	11:3659748011:36597480	G/A	-	SNP	Multiple_observations,Frequency,ESP,	Missense variant	E/K	876	31deleterious(0.03)	1benign(0)
rs104894291	11:3659604111:36596041	G/T/A	-	SNP	Multiple_observations,Frequency,Cited,ESP,	Missense variant	R/H	396	1deleterious(0)	874possibly damaging(0.873)
rs104894291	11:3659604111:36596041	G/T/A	-	SNP	Multiple_observations,Frequency,Cited,ESP,	Missense variant	R/L	396	1deleterious(0)	760possibly damaging(0.759)
rs141524540	11:3659615711:36596157	A/G	-	SNP	Multiple_observations,Frequency,Cited,ESP,	Missense variant	M/V	435	1deleterious(0)	1benign(0)
rs34357808	11:3659504311:36595043	A/G	0.011 (G)	SNP	Multiple_observations,Frequency,1000Genomes,ESP,	Synonymous variant	P	63	-	-
rs138676205	11:3659514911:36595149	G/A	0.003 (A)	SNP	Multiple_observations,Frequency,1000Genomes,ESP,	Missense variant	G/S	99	91tolerated(0.09)	1benign(0)
rs76897604	11:3659557911:36595579	A/G	0.003 (G)	SNP	Multiple_observations,Frequency,1000Genomes,ESP,	Missense variant	Q/R	242	1001tolerated(1)	1benign(0)
rs141560248	11:3659742511:36597425	C/T	0.001 (T)	SNP	Multiple_observations,Frequency,1000Genomes,ESP,	Synonymous variant	A	857	-	-
rs139113046	11:3659787011:36597870	A/G	0.001 (G)	SNP	Multiple_observations,Frequency,1000Genomes,ESP,	Missense variant	M/V	1006	1deleterious(0)	127benign(0.126)
rs12282321	11:3658461711:36584617	G/T	0.016 (T)	SNP	Multiple_observations,Frequency,1000Genomes,	Upstream gene variant	-	-	-	-
rs73453392	11:3658469611:36584696	G/A	0.012 (A)	SNP	Multiple_observations,Frequency,1000Genomes,	Upstream gene variant	-	-	-	-
rs73453395	11:3658479211:36584792	A/G	0.007 (G)	SNP	Multiple_observations,Frequency,1000Genomes,	Upstream gene variant	-	-	-	-
rs11033696	11:3658521911:36585219	C/T	0.164 (T)	SNP	Multiple_observations,Frequency,1000Genomes,	Upstream gene variant	-	-	-	-
rs73453397	11:3658576011:36585760	C/G	0.013 (G)	SNP	Multiple_observations,Frequency,1000Genomes,	Upstream gene variant	-	-	-	-
rs111610814	11:3658669211:36586692	A/G	0.065 (G)	SNP	Multiple_observations,Frequency,1000Genomes,	Upstream gene variant	-	-	-	-
rs73453401	11:3658676811:36586768	C/T	0.070 (T)	SNP	Multiple_observations,Frequency,1000Genomes,	Upstream gene variant	-	-	-	-
rs114563822	11:3658690811:36586908	C/T	0.025 (T)	SNP	Multiple_observations,Frequency,1000Genomes,	Upstream gene variant	-	-	-	-
rs73455505	11:3658726711:36587267	G/A	0.012 (A)	SNP	Multiple_observations,Frequency,1000Genomes,	Upstream gene variant	-	-	-	-
rs114990708	11:3658783011:36587830	G/A	0.010 (A)	SNP	Multiple_observations,Frequency,1000Genomes,	Upstream gene variant	-	-	-	-
rs12279639	11:3658807811:36588078	G/C	0.018 (C)	SNP	Multiple_observations,Frequency,1000Genomes,	Upstream gene variant	-	-	-	-
rs4150996	11:3658840311:36588403	C/G/T	0.012 (T)	SNP	Multiple_observations,Frequency,1000Genomes,	Upstream gene variant	-	-	-	-
rs4150996	11:3658840311:36588403	C/G/T	0.012 (T)	SNP	Multiple_observations,Frequency,1000Genomes,	Upstream gene variant	-	-	-	-
rs872052	11:3658957311:36589573	G/A	0.001 (A)	SNP	Multiple_observations,Frequency,1000Genomes,	Upstream gene variant	-	-	-	-
rs4151004	11:3659086111:36590861	G/A	0.022 (A)	SNP	Multiple_observations,Frequency,1000Genomes,	Intron variant	-	-	-	-
rs4151006	11:3659176111:36591761	C/T	0.001 (T)	SNP	Multiple_observations,Frequency,1000Genomes,	Intron variant	-	-	-	-
rs4151008	11:3659192011:36591920	A/G	0.012 (G)	SNP	Multiple_observations,Frequency,1000Genomes,	Intron variant	-	-	-	-
rs4151011	11:3659208211:36592082	T/C	0.023 (C)	SNP	Multiple_observations,Frequency,1000Genomes,	Intron variant	-	-	-	-
rs112287696	11:3659240011:36592400	C/T	0.005 (T)	SNP	Multiple_observations,Frequency,1000Genomes,	Intron variant	-	-	-	-
rs113248703	11:3659256411:36592564	C/A	0.005 (A)	SNP	Multiple_observations,Frequency,1000Genomes,	Intron variant	-	-	-	-
rs4151013	11:3659316411:36593164	C/T	0.019 (T)	SNP	Multiple_observations,Frequency,1000Genomes,	Intron variant	-	-	-	-
rs4151014	11:3659336211:36593362	A/T	0.027 (T)	SNP	Multiple_observations,Frequency,1000Genomes,	Intron variant	-	-	-	-
rs4151015	11:3659343111:36593431	G/A	0.002 (A)	SNP	Multiple_observations,Frequency,1000Genomes,	Intron variant	-	-	-	-
rs74534829	11:3659353011:36593530	G/T	0.006 (T)	SNP	Multiple_observations,Frequency,1000Genomes,	Intron variant	-	-	-	-
rs75083727	11:3659373611:36593736	C/A	0.004 (A)	SNP	Multiple_observations,Frequency,1000Genomes,	Intron variant	-	-	-	-
rs1515061	11:3659396711:36593967	A/T	0.001 (T)	SNP	Multiple_observations,Frequency,1000Genomes,	Intron variant	-	-	-	-
rs4151042	11:3659948011:36599480	A/C	0.004 (C)	SNP	Multiple_observations,Frequency,1000Genomes,	3 prime UTR variant	-	-	-	-
rs113179198	11:3660150111:36601501	C/T	0.005 (T)	SNP	Multiple_observations,Frequency,1000Genomes,	Downstream gene variant	-	-	-	-
rs111728084	11:3660165611:36601656	A/G	0.088 (G)	SNP	Multiple_observations,Frequency,1000Genomes,	Downstream gene variant	-	-	-	-
rs4151051	11:3660187211:36601872	G/A	0.106 (A)	SNP	Multiple_observations,Frequency,1000Genomes,	Downstream gene variant	-	-	-	-
rs61880060	11:3660206511:36602065	G/C	0.161 (C)	SNP	Multiple_observations,Frequency,1000Genomes,	Downstream gene variant	-	-	-	-
rs73455516	11:3660295811:36602958	C/T	0.070 (T)	SNP	Multiple_observations,Frequency,1000Genomes,	Downstream gene variant	-	-	-	-
rs73455517	11:3660306511:36603065	A/C	0.126 (C)	SNP	Multiple_observations,Frequency,1000Genomes,	Downstream gene variant	-	-	-	-
rs111360078	11:3660314611:36603146	G/A	0.004 (A)	SNP	Multiple_observations,Frequency,1000Genomes,	Downstream gene variant	-	-	-	-
rs113270047	11:3660329711:36603297	C/T	0.126 (T)	SNP	Multiple_observations,Frequency,1000Genomes,	Downstream gene variant	-	-	-	-
rs73455518	11:3660373311:36603733	A/G	0.070 (G)	SNP	Multiple_observations,Frequency,1000Genomes,	Downstream gene variant	-	-	-	-
rs112079096	11:3660471211:36604712	A/G	0.009 (G)	SNP	Multiple_observations,Frequency,1000Genomes,	Downstream gene variant	-	-	-	-
rs76254210	11:3659248911:36592489	T/C	-	SNP	Multiple_observations,Frequency,	Intron variant	-	-	-	-
rs4151022	11:3659414711:36594147	C/T	-	SNP	Multiple_observations,Frequency,	Intron variant	-	-	-	-
rs4151048	11:3660068411: between 36600684 & 36600685	-/T	0.071 (T)	insertion	Multiple_observations,Frequency,	Feature elongation, 3 prime UTR var	-	-	-	-
rs35085546	11:3660330211: between 36603302 & 36603303	-/G	-	insertion	Multiple_observations,Frequency,	Downstream gene variant	-	-	-	-
rs104894285	11:3659653511:36596535	C/T	-	SNP	Multiple_observations,ESP,	Missense variant	R/C	561	1deleterious(0)	976probably damaging(0.975)
rs104894286	11:3659706411:36597064	G/A	-	SNP	Multiple_observations,ESP,	Missense variant	R/H	737	1deleterious(0)	965probably damaging(0.964)
rs104894289	11:3659604011:36596040	C/T	-	SNP	Multiple_observations,Cited,	Missense variant	R/C	396	1deleterious(0)	910probably damaging(0.909)
rs148380512	11:3659671811:36596718	G/A	0.001 (A)	SNP	Multiple_observations,1000Genomes,ESP,	Missense variant	A/T	622	1deleterious(0)	957probably damaging(0.956)
rs147887098	11:3660140511:36601405	A/G	0.001 (G)	SNP	Multiple_observations,1000Genomes,	Downstream gene variant	-	-	-	-
rs7924442	11:3660316611:36603166	G/C	0.019 (C)	SNP	Multiple_observations,1000Genomes,	Downstream gene variant	-	-	-	-
rs141180078	11:3660410011:36604100	G/A	0.016 (A)	SNP	Multiple_observations,1000Genomes,	Downstream gene variant	-	-	-	-
rs76511022	11:3660433311:36604333	T/C	0.016 (C)	SNP	Multiple_observations,1000Genomes,	Downstream gene variant	-	-	-	-
rs12275170	11:3660551611:36605516	C/A	0.003 (A)	SNP	Multiple_observations,1000Genomes,	Downstream gene variant	-	-	-	-
rs72236516	11:3658514811:36585148-36585151	ACTA/-	0.166 (-)	deletion	Multiple_observations,	Upstream gene variant	-	-	-	-

rs58012522	11:3658515111:36585151-36585154	AACT/-	-	deletion	Multiple_observations,	Upstream gene variant	-	-		-	-
rs137987566	11:3658763211:36587632-36587633	AG/-	-	deletion	Multiple_observations,	Upstream gene variant	-	-		-	-
rs1399601	11:3658789111:36587891	G/C	-	SNP	Multiple_observations,	Upstream gene variant	-	-		-	-
rs76177616	11:3659244611:36592446	G/A	-	SNP	Multiple_observations,	Intron variant	-	-		-	-
rs1801203	11:3659532111:36595321	C/T	-	SNP	Multiple_observations,	Missense variant	A/V		156	371tolerated(0.37)	847possibly damaging(0.846)
rs104894292	11:3659614011:36596140	A/G	-	SNP	Multiple_observations,	Missense variant	D/G		429	1deleterious(0)	847possibly damaging(0.846)
rs104894284	11:3659653611:36596536	G/A	-	SNP	Multiple_observations,	Missense variant	R/H		561	1deleterious(0)	965probably damaging(0.964)
rs28933392	11:3659701811:36597018	G/A	-	SNP	Multiple_observations,	Missense variant	E/K		722	21deleterious(0.02)	877possibly damaging(0.876)
rs104894282	11:3659717411:36597174	G/T	-	SNP	Multiple_observations,	Stop gained	E/*		774	-	-
rs104894287	11:3659737511:36597375	C/T	-	SNP	Multiple_observations,	Missense variant	R/W		841	1deleterious(0)	976probably damaging(0.975)
rs104894290	11:3659758911:36597589	A/G	-	SNP	Multiple_observations,	Missense variant	Y/C		912	1deleterious(0)	985probably damaging(0.984)
rs104894283	11:3659766811:36597668	T/G	-	SNP	Multiple_observations,	Stop gained	Y/*		938	-	-
rs104894288	11:3659779611:36597796	A/C	-	SNP	Multiple_observations,	Missense variant	Q/P		981	1deleterious(0)	545possibly damaging(0.544)
rs141384582	11:3660109711:36601097	T/-	-	deletion	Multiple_observations,	3 prime UTR variant, Feature trunca	-	-		-	-
rs2673016	11:3660278311:36602783	C/T	-	SNP	Multiple_observations,	Downstream gene variant	-	-		-	-
rs111978058	11:3660329811: between 36603298 & 36603299	-/G	-	insertion	Multiple_observations,	Downstream gene variant	-	-		-	-
rs72124072	11:3660628211:36606282	A/-	0.126 (-)	deletion	Multiple_observations,	Downstream gene variant	-	-		-	-
rs149364682	11:3659494811:36594948	C/G	-	SNP	Frequency,ESP,	Missense variant	L/V		32	1deleterious(0)	627possibly damaging(0.626)
rs148700564	11:3659513611:36595136	C/T	-	SNP	Frequency,ESP,	Synonymous variant	N		94	-	-
rs142057334	11:3659513811:36595138	A/G	-	SNP	Frequency,ESP,	Missense variant	E/G		95	391tolerated(0.39)	1benign(0)
rs146457887	11:3659518811:36595188	C/T	-	SNP	Frequency,ESP,	Missense variant	R/C		112	1deleterious(0)	910probably damaging(0.909)
rs140648865	11:3659527011:36595270	G/T	-	SNP	Frequency,ESP,	Missense variant	G/V		139	501tolerated(0.5)	1benign(0)
rs148288583	11:3659545111:36595451	C/T	-	SNP	Frequency,ESP,	Synonymous variant	N		199	-	-
rs141561979	11:3659550511:36595505	C/T	-	SNP	Frequency,ESP,	Synonymous variant	A		217	-	-
rs147203889	11:3659559311:36595593	C/G	-	SNP	Frequency,ESP,	Missense variant	R/G		247	21deleterious(0.02)	5benign(0.004)
rs144616804	11:3659560511:36595605	A/G	-	SNP	Frequency,ESP,	Missense variant	R/G		251	201tolerated(0.2)	3benign(0.002)
rs148393376	11:3659565311:36595653	G/A	-	SNP	Frequency,ESP,	Missense variant	A/T		267	1001tolerated(1)	1benign(0)
rs145962212	11:3659577111:36595771	C/T	-	SNP	Frequency,ESP,	Missense variant	T/I		306	1deleterious(0)	998probably damaging(0.997)
rs139883723	11:3659581011:36595810	G/C	-	SNP	Frequency,ESP,	Missense variant	R/T		319	1deleterious(0)	3benign(0.002)
rs144391165	11:3659582111:36595821	G/A	-	SNP	Frequency,ESP,	Missense variant	V/I		323	51deleterious(0.05)	263benign(0.262)
rs145877904	11:3659615311:36596153	G/C	-	SNP	Frequency,ESP,	Synonymous variant	V		433	-	-
rs138387845	11:3659622511:36596225	C/T	-	SNP	Frequency,ESP,	Synonymous variant	I		457	-	-
rs142486791	11:3659625411:36596254	C/T	-	SNP	Frequency,ESP,	Missense variant	P/L		467	1deleterious(0)	939probably damaging(0.938)
rs148235228	11:3659649111:36596491	C/T	-	SNP	Frequency,ESP,	Missense variant	S/F		546	221tolerated(0.22)	963probably damaging(0.962)
rs141265699	11:3659651911:36596519	C/T	-	SNP	Frequency,ESP,	Synonymous variant	T		555	-	-
rs138101978	11:3659655911:36596559	G/T	-	SNP	Frequency,ESP,	Missense variant	A/S		569	1deleterious(0)	932probably damaging(0.931)
rs145951370	11:3659665511:36596655	T/A	-	SNP	Frequency,ESP,	Missense variant	S/T		601	11deleterious(0.01)	806possibly damaging(0.805)
rs144503943	11:3659694211:36596942	C/G	-	SNP	Frequency,ESP,	Synonymous variant	G		696	-	-
rs148763154	11:3659703511:36597035	C/G	-	SNP	Frequency,ESP,	Synonymous variant	V		727	-	-
rs142419805	11:3659716711:36597167	T/C	-	SNP	Frequency,ESP,	Synonymous variant	S		771	-	-
rs142345523	11:3659740611:36597406	G/A	-	SNP	Frequency,ESP,	Missense variant	R/K		851	21deleterious(0.02)	719possibly damaging(0.718)
rs138119069	11:3659756911:36597569	C/T	-	SNP	Frequency,ESP,	Synonymous variant	C		905	-	-
rs144893101	11:3659762811:36597628	C/T	-	SNP	Frequency,ESP,	Missense variant	T/M		925	1deleterious(0)	986probably damaging(0.985)
rs138510915	11:3659772811:36597728	A/G	-	SNP	Frequency,ESP,	Synonymous variant	A		958	-	-
rs114432633	11:3658496111:36584961	T/G	0.013 (G)	SNP	Frequency,1000Genomes,	Upstream gene variant	-	-		-	-
rs115977457	11:3658546211:36585462	T/G	0.018 (G)	SNP	Frequency,1000Genomes,	Upstream gene variant	-	-		-	-
rs115620583	11:3658599511:36585995	A/G	0.003 (G)	SNP	Frequency,1000Genomes,	Upstream gene variant	-	-		-	-
rs115073791	11:3658632411:36586324	C/T	0.020 (T)	SNP	Frequency,1000Genomes,	Upstream gene variant	-	-		-	-
rs115387428	11:3658670611:36586706	G/A	0.018 (A)	SNP	Frequency,1000Genomes,	Upstream gene variant	-	-		-	-
rs114954186	11:3658673111:36586731	A/G	0.020 (G)	SNP	Frequency,1000Genomes,	Upstream gene variant	-	-		-	-
rs115022045	11:3658918711:36589187	A/G	0.006 (G)	SNP	Frequency,1000Genomes,	Upstream gene variant	-	-		-	-
rs117817879	11:3658954111:36589541	C/T	0.005 (T)	SNP	Frequency,1000Genomes,	Upstream gene variant	-	-		-	-
rs115789056	11:3659221111:36592211	C/T	0.010 (T)	SNP	Frequency,1000Genomes,	Intron variant	-	-		-	-
rs115582302	11:3659842611:36598426	A/G	0.004 (G)	SNP	Frequency,1000Genomes,	3 prime UTR variant	-	-		-	-
rs116411362	11:3660219211:36602192	G/T	0.003 (T)	SNP	Frequency,1000Genomes,	Downstream gene variant	-	-		-	-
rs117683643	11:3660290411:36602904	C/T	0.005 (T)	SNP	Frequency,1000Genomes,	Downstream gene variant	-	-		-	-
rs114653635	11:3660324811:36603248	G/A	0.009 (A)	SNP	Frequency,1000Genomes,	Downstream gene variant	-	-		-	-
rs115242321	11:3660351411:36603514	T/A	0.014 (A)	SNP	Frequency,1000Genomes,	Downstream gene variant	-	-		-	-
rs78967763	11:3660370611:36603706	C/G	0.018 (G)	SNP	Frequency,1000Genomes,	Downstream gene variant	-	-		-	-
rs114549317	11:3660375611:36603756	C/G	0.021 (G)	SNP	Frequency,1000Genomes,	Downstream gene variant	-	-		-	-

rs118170873	11:3660390111:36603901	A/G	0.003 (G)	SNP	Frequency,1000Genomes,	Downstream gene variant	-	-		-	-
rs114495988	11:3660396611:36603966	A/T	0.004 (T)	SNP	Frequency,1000Genomes,	Downstream gene variant	-	-		-	-
rs74524795	11:3658568711:36585687	A/C	-	SNP	Frequency,	Upstream gene variant	-	-		-	-
rs113291580	11:3658938711:36589387	G/C	-	SNP	Frequency,	Upstream gene variant	-	-		-	-
rs4151003	11:3659039711:36590397	A/G	-	SNP	Frequency,	Intron variant	-	-		-	-
rs111793475	11:3659116211:36591162	G/C	-	SNP	Frequency,	Intron variant	-	-		-	-
rs4151009	11:3659200711:36592007	A/G	-	SNP	Frequency,	Intron variant	-	-		-	-
rs4151010	11:3659208111:36592081	A/T	-	SNP	Frequency,	Intron variant	-	-		-	-
rs111789593	11:3659230011:36592300	T/A	-	SNP	Frequency,	Intron variant	-	-		-	-
rs4151020	11:3659388811:36593888	G/T	-	SNP	Frequency,	Intron variant	-	-		-	-
rs4151021	11:3659401111:36594011	C/A	-	SNP	Frequency,	Intron variant	-	-		-	-
rs113502270	11:3659483511:36594835	T/C	-	SNP	Frequency,	Intron variant, Splice region variant	-	-		-	-
rs144019501	11:3659517311:36595173	C/G	-	SNP	Frequency,	Missense variant	L/V		107	11deleterious(0.01)	627possibly damaging(0.626)
rs4151026	11:3659519011: between 36595190 & 36595191	-/C	-	insertion	Frequency,	Feature elongation, Frameshift variant			113	-	-
rs4151028	11:3659548411:36595484	C/T/A	-	SNP	Frequency,	Synonymous variant	S		210	-	-
rs4151028	11:3659548411:36595484	C/T/A	-	SNP	Frequency,	Synonymous variant	S		210	-	-
rs147055289	11:3659551011:36595510	G/A	-	SNP	Frequency,	Missense variant	R/Q		219	221tolerated(0.22)	1benign(0)
rs138419861	11:3659551611:36595516	T/A	-	SNP	Frequency,	Missense variant	L/H		221	231tolerated(0.23)	939probably damaging(0.938)
rs112047157	11:3659631311:36596313	A/G	-	SNP	Frequency,	Missense variant	M/V		487	1deleterious(0)	127benign(0.126)
rs75591129	11:3659764611:36597646	A/C	-	SNP	Frequency,	Missense variant	Y/S		931	11deleterious(0.01)	950probably damaging(0.949)
rs4151035	11:3659822411:36598224	G/A	-	SNP	Frequency,	3 prime UTR variant	-	-		-	-
rs4151037	11:3659840611:36598406	T/C	-	SNP	Frequency,	3 prime UTR variant	-	-		-	-
rs4151039	11:3659872511:36598725	C/T	-	SNP	Frequency,	3 prime UTR variant	-	-		-	-
rs112766186	11:3659908611:36599086	C/T	-	SNP	Frequency,	3 prime UTR variant	-	-		-	-
rs113060327	11:3659926111:36599261	A/G	-	SNP	Frequency,	3 prime UTR variant	-	-		-	-
rs4151043	11:3659991911:36599919	C/G	-	SNP	Frequency,	3 prime UTR variant	-	-		-	-
rs4151049	11:3660129311:36601293	A/-	-	deletion	Frequency,	3 prime UTR variant, Feature trunca	-	-		-	-
rs113674944	11:3660137911:36601379	G/A	-	SNP	Frequency,	Downstream gene variant	-	-		-	-
rs113728396	11:3660164711:36601647	C/T	-	SNP	Frequency,	Downstream gene variant	-	-		-	-
rs4151050	11:3660171111:36601711	G/A	-	SNP	Frequency,	Downstream gene variant	-	-		-	-
TMP_ESP_11_36594833	11:3659483311:36594833	G/A	-	SNP	ESP,	Intron variant, Splice region variant	-	-		-	-
rs200575481	11:3659485511:36594855	A/G	-	SNP	ESP,	Initiator codon variant	M/V		1	1deleterious(0)	127benign(0.126)
rs150201913	11:3659491011:36594910	A/G	-	SNP	ESP,	Missense variant	Q/R		19	71tolerated(0.07)	424benign(0.423)
TMP_ESP_11_36594953	11:3659495311:36594953	C/A	-	SNP	ESP,	Missense variant	F/L		33	1deleterious(0)	511possibly damaging(0.51)
TMP_ESP_11_36594955	11:3659495511:36594955	G/A	-	SNP	ESP,	Missense variant	R/Q		34	1deleterious(0)	644possibly damaging(0.643)
TMP_ESP_11_36595005	11:3659500511:36595005	G/T	-	SNP	ESP,	Missense variant	D/Y		51	41deleterious(0.04)	37benign(0.036)
rs150199231	11:3659510511:36595105	A/G	0.005 (G)	SNP	ESP,	Missense variant	H/R		84	551tolerated(0.55)	629possibly damaging(0.628)
TMP_ESP_11_36595109	11:3659511011:36595110-36595111	AA/-	-	deletion	ESP,	Frameshift variant, Feature truncation			86	-	-
TMP_ESP_11_36595111	11:3659511111:36595111	A/T	-	SNP	ESP,	Missense variant	K/M		86	81tolerated(0.08)	37benign(0.036)
TMP_ESP_11_36595175	11:3659517511:36595175	T/G	-	SNP	ESP,	Synonymous variant	L		107	-	-
rs193922464	11:3659517611:36595176	C/T	-	SNP	ESP,	Stop gained	R/*		108	-	-
TMP_ESP_11_36595279	11:3659527911:36595279	G/A	-	SNP	ESP,	Missense variant	R/Q		142	11deleterious(0.01)	644possibly damaging(0.643)
TMP_ESP_11_36595310	11:3659531011:36595310	G/T	-	SNP	ESP,	Synonymous variant	P		152	-	-
TMP_ESP_11_36595337	11:3659533711:36595337	C/T	-	SNP	ESP,	Synonymous variant	I		161	-	-
TMP_ESP_11_36595365	11:3659536511:36595365	C/T	-	SNP	ESP,	Missense variant	H/Y		171	31deleterious(0.03)	511possibly damaging(0.51)
TMP_ESP_11_36595367	11:3659536711:36595367	C/G	-	SNP	ESP,	Missense variant	H/Q		171	11deleterious(0.01)	745possibly damaging(0.744)
TMP_ESP_11_36595396	11:3659539611:36595396	G/T	-	SNP	ESP,	Missense variant	S/I		181	51tolerated(0.05)	8benign(0.007)
TMP_ESP_11_36595413	11:3659541311:36595413	T/A	-	SNP	ESP,	Missense variant	F/I		187	131tolerated(0.13)	629possibly damaging(0.628)
TMP_ESP_11_36595445	11:3659544511:36595445	G/A	-	SNP	ESP,	Synonymous variant	P		197	-	-
rs202178215	11:3659550711:36595507	G/A	-	SNP	ESP,	Missense variant	R/H		218	371tolerated(0.37)	874possibly damaging(0.873)
TMP_ESP_11_36595509	11:3659550911:36595509	C/T	-	SNP	ESP,	Missense variant	R/W		219	81tolerated(0.08)	37benign(0.036)
TMP_ESP_11_36595630	11:3659563011:36595630	G/T	-	SNP	ESP,	Missense variant	S/I		259	51deleterious(0.05)	8benign(0.007)
TMP_ESP_11_36595657	11:3659565711:36595657	A/G	-	SNP	ESP,	Missense variant	N/S		268	161tolerated(0.16)	1benign(0)
TMP_ESP_11_36595669	11:3659566911:36595669	T/A	-	SNP	ESP,	Missense variant	I/K		272	11deleterious(0.01)	605possibly damaging(0.604)
TMP_ESP_11_36595678	11:3659567811:36595678	G/A	-	SNP	ESP,	Missense variant	S/N		275	81tolerated(0.08)	290benign(0.289)
TMP_ESP_11_36595694	11:3659569411:36595694	A/G	-	SNP	ESP,	Synonymous variant	A		280	-	-
TMP_ESP_11_36595706	11:3659570611:36595706	A/G	-	SNP	ESP,	Synonymous variant	P		284	-	-
rs201462389	11:3659582811:36595828	G/A	0.001 (A)	SNP	ESP,	Missense variant	G/D		325	1deleterious(0)	994probably damaging(0.993)
TMP_ESP_11_36595858	11:3659585811:36595858	G/T	-	SNP	ESP,	Missense variant	C/F		335	21deleterious(0.02)	972probably damaging(0.971)
rs151077440	11:3659591811:36595918	T/C	-	SNP	ESP,	Missense variant	M/T		355	511tolerated(0.51)	1benign(0)

TMP_ESP_11_36595922	11:3659592211:36595922	G/A	-	SNP	ESP,	Synonymous variant	V		356	-	-
TMP_ESP_11_36595944	11:3659594411:36595944	A/G	-	SNP	ESP,	Missense variant	N/D		364	1001tolerated(1)	1benign(0)
TMP_ESP_11_36596042	11:3659604211:36596042	C/G	-	SNP	ESP,	Synonymous variant	R		396	-	-
rs202189218	11:3659606111:36596061	A/T	-	SNP	ESP,	Missense variant	T/S		403	11deleterious(0.01)	627possibly damaging(0.626)
TMP_ESP_11_36596090	11:3659609011:36596090	G/A	-	SNP	ESP,	Synonymous variant	R		412	-	-
TMP_ESP_11_36596152	11:3659615211:36596152	T/C	-	SNP	ESP,	Missense variant	V/A		433	1deleterious(0)	627possibly damaging(0.626)
rs199474678	11:3659627411:36596274	C/T	-	SNP	ESP,	Missense variant	R/C		474	1deleterious(0)	910probably damaging(0.909)
TMP_ESP_11_36596331	11:3659633111:36596331	G/A	-	SNP	ESP,	Missense variant	A/T		493	1deleterious(0)	847possibly damaging(0.846)
TMP_ESP_11_36596378	11:3659637811:36596378	T/C	-	SNP	ESP,	Synonymous variant	N		508	-	-
rs193922461	11:3659642011:36596420	G/T	-	SNP	ESP,	Missense variant	W/C		522	21deleterious(0.02)	985probably damaging(0.984)
TMP_ESP_11_36596453	11:3659645311:36596453	T/A	-	SNP	ESP,	Synonymous variant	T		533	-	-
rs150790148	11:3659652811:36596528	G/C	-	SNP	ESP,	Missense variant	K/N		558	1deleterious(0)	950probably damaging(0.949)
TMP_ESP_11_36596550	11:3659655011:36596550	T/C	-	SNP	ESP,	Synonymous variant	L		566	-	-
rs199474688	11:3659672411:36596724	C/T	-	SNP	ESP,	Missense variant	R/C		624	1deleterious(0)	976probably damaging(0.975)
TMP_ESP_11_36596950	11:3659695011:36596950	G/A	-	SNP	ESP,	Missense variant	R/Q		699	1deleterious(0)	878possibly damaging(0.877)
TMP_ESP_11_36596996	11:3659699711:36596997	G/-	-	deletion	ESP,	Frameshift variant, Feature truncation			715	-	-
TMP_ESP_11_36597013	11:3659701311:36597013	G/A	-	SNP	ESP,	Missense variant	G/D		720	1deleterious(0)	995probably damaging(0.994)
TMP_ESP_11_36597017	11:3659701711:36597017	C/T	-	SNP	ESP,	Synonymous variant	L		721	-	-
TMP_ESP_11_36597091	11:3659709111:36597091	T/G	-	SNP	ESP,	Missense variant	F/C		746	11deleterious(0.01)	88benign(0.087)
TMP_ESP_11_36597164	11:3659716411:36597164	G/A	-	SNP	ESP,	Synonymous variant	E		770	-	-
TMP_ESP_11_36597165	11:3659716511:36597165	T/A	-	SNP	ESP,	Missense variant	S/T		771	231tolerated(0.23)	1benign(0)
TMP_ESP_11_36597172	11:3659717211:36597172	A/G	-	SNP	ESP,	Missense variant	E/G		773	11deleterious(0.01)	9benign(0.008)
rs151322536	11:3659725811:36597258	G/A	-	SNP	ESP,	Missense variant	G/S		802	21deleterious(0.02)	988probably damaging(0.987)
rs186717025	11:3659738111:36597381	A/G	0.001 (G)	SNP	ESP,	Missense variant	K/E		843	1deleterious(0)	9benign(0.008)
TMP_ESP_11_36597423	11:3659742311:36597423	G/T	-	SNP	ESP,	Missense variant	A/S		857	21deleterious(0.02)	932probably damaging(0.931)
TMP_ESP_11_36597448	11:3659744911:36597449-36597450	TG/-	-	deletion	ESP,	Frameshift variant, Feature truncation			865	-	-
rs200264827	11:3659747911:36597479	C/G	0.001 (G)	SNP	ESP,	Synonymous variant	S		875	-	-
rs150721661	11:3659760511:36597605	G/A	0.005 (A)	SNP	ESP,	Synonymous variant	Q		917	-	-
TMP_ESP_11_36597607	11:3659760711:36597607	G/A	-	SNP	ESP,	Missense variant	R/H		918	61tolerated(0.06)	965probably damaging(0.964)
rs199921936	11:3659766211:36597662	C/A	-	SNP	ESP,	Synonymous variant	T		936	-	-
TMP_ESP_11_36597698	11:3659769811:36597698	A/T	-	SNP	ESP,	Missense variant	E/D		948	1deleterious(0)	870possibly damaging(0.869)
rs150739647	11:3659777811:36597778	G/A	-	SNP	ESP,	Missense variant	R/Q		975	1deleterious(0)	644possibly damaging(0.643)
TMP_ESP_11_36597845	11:3659784511:36597845	C/T	-	SNP	ESP,	Synonymous variant	Y		997	-	-
TMP_ESP_11_36597857	11:3659785711:36597857	C/T	-	SNP	ESP,	Synonymous variant	Y		1001	-	-
TMP_ESP_11_36597879	11:3659787911:36597879	C/T	-	SNP	ESP,	Missense variant	H/Y		1009	1deleterious(0)	511possibly damaging(0.51)
TMP_ESP_11_36597943	11:3659794311:36597943	G/T	-	SNP	ESP,	Missense variant	G/V		1030	121tolerated(0.12)	56benign(0.055)
TMP_ESP_11_36597971	11:3659797111:36597971	T/G	-	SNP	ESP,	Missense variant	D/E		1039	101tolerated(0.1)	4benign(0.003)
TMP_ESP_11_36597993	11:3659799311:36597993	C/T	-	SNP	ESP,	3 prime UTR variant	-	-		-	-
rs147440161	11:3659548311:36595483	C/T	-	somatic_S	Cited,	Missense variant	S/F		210	191tolerated(0.19)	27benign(0.026)
rs143969029	11:3659637411:36596374	G/A	0.001 (A)	SNP	1000Genomes,ESP,	Missense variant	R/Q		507	1deleterious(0)	644possibly damaging(0.643)
rs139494020	11:3658459811:36584598	C/G	0.006 (G)	SNP	1000Genomes,	Upstream gene variant	-	-		-	-
rs145046096	11:3658465411:36584654	C/T	0.001 (T)	SNP	1000Genomes,	Upstream gene variant	-	-		-	-
rs140777638	11:3658535711:36585357	G/T	0.006 (T)	SNP	1000Genomes,	Upstream gene variant	-	-		-	-
rs145584687	11:3658551011:36585510	C/T	0.001 (T)	SNP	1000Genomes,	Upstream gene variant	-	-		-	-
rs148875767	11:3658553611:36585536	C/G	0.001 (G)	SNP	1000Genomes,	Upstream gene variant	-	-		-	-
rs145589374	11:3658563111:36585631	A/G	0.001 (G)	SNP	1000Genomes,	Upstream gene variant	-	-		-	-
rs138113175	11:3658635211:36586352	C/G	0.004 (G)	SNP	1000Genomes,	Upstream gene variant	-	-		-	-
rs142639510	11:3658636611:36586366	C/T	0.004 (T)	SNP	1000Genomes,	Upstream gene variant	-	-		-	-
rs145640682	11:3658639611:36586396	T/C	0.001 (C)	SNP	1000Genomes,	Upstream gene variant	-	-		-	-
rs148076313	11:3658641911:36586419	T/A	0.001 (A)	SNP	1000Genomes,	Upstream gene variant	-	-		-	-
rs141913434	11:3658653411:36586534	T/C	0.001 (C)	SNP	1000Genomes,	Upstream gene variant	-	-		-	-
rs139913944	11:3658703411:36587034	A/G	0.006 (G)	SNP	1000Genomes,	Upstream gene variant	-	-		-	-
rs143196268	11:3658703711:36587037	C/A	0.002 (A)	SNP	1000Genomes,	Upstream gene variant	-	-		-	-
rs147500932	11:3658717411:36587174	G/T	0.001 (T)	SNP	1000Genomes,	Upstream gene variant	-	-		-	-
rs140212393	11:3658732511:36587325	A/C	0.001 (C)	SNP	1000Genomes,	Upstream gene variant	-	-		-	-
rs145610899	11:3658735511:36587355	T/C	0.001 (C)	SNP	1000Genomes,	Upstream gene variant	-	-		-	-
rs144896778	11:3658807611:36588076	G/C	0.006 (C)	SNP	1000Genomes,	Upstream gene variant	-	-		-	-
rs148608607	11:3658829811:36588298	A/G	0.001 (G)	SNP	1000Genomes,	Upstream gene variant	-	-		-	-
rs144104412	11:3658922311:36589223	G/T	0.001 (T)	SNP	1000Genomes,	Upstream gene variant	-	-		-	-
rs141124967	11:3658991311:36589913	A/G	0.001 (G)	SNP	1000Genomes,	Intron variant	-	-		-	-

rs144872922	11:3659024511:36590245	G/A	0.001 (A)	SNP	1000Genomes,	Intron variant	-	-	-	-
rs138179285	11:3659036411:36590364	A/C	0.006 (C)	SNP	1000Genomes,	Intron variant	-	-	-	-
rs149133410	11:3659041011:36590410	G/A	0.001 (A)	SNP	1000Genomes,	Intron variant	-	-	-	-
rs148329600	11:3659054811:36590548	T/C	0.001 (A)	SNP	1000Genomes,	Intron variant	-	-	-	-
rs143081800	11:3659060911:36590609	G/C	0.001 (C)	SNP	1000Genomes,	Intron variant	-	-	-	-
rs147510686	11:3659095011:36590950	A/G	0.003 (G)	SNP	1000Genomes,	Intron variant	-	-	-	-
rs138443357	11:3659110411:36591104	T/C	0.001 (C)	SNP	1000Genomes,	Intron variant	-	-	-	-
rs139477887	11:3659239211:36592392	A/C	0.001 (C)	SNP	1000Genomes,	Intron variant	-	-	-	-
rs144021230	11:3659380411:36593804	G/C	0.002 (C)	SNP	1000Genomes,	Intron variant	-	-	-	-
rs146498720	11:3659392111:36593921	C/T	0.001 (T)	SNP	1000Genomes,	Intron variant	-	-	-	-
rs142236848	11:3659449111:36594491	C/A	0.001 (A)	SNP	1000Genomes,	Intron variant	-	-	-	-
rs141654332	11:3659565211:36595652	C/T	0.001 (T)	SNP	1000Genomes,	Synonymous variant	I	266	-	-
rs139084848	11:3659780911:36597809	T/C	0.001 (C)	SNP	1000Genomes,	Synonymous variant	Y	985	-	-
rs144069419	11:3659825911:36598259	G/T	0.001 (C)	SNP	1000Genomes,	3 prime UTR variant	-	-	-	-
rs145963034	11:3659908711:36599087	G/A	0.001 (A)	SNP	1000Genomes,	3 prime UTR variant	-	-	-	-
rs139938937	11:3659915211:36599152	C/A	0.001 (A)	SNP	1000Genomes,	3 prime UTR variant	-	-	-	-
rs145552187	11:3659974711:36599747	G/A	0.003 (A)	SNP	1000Genomes,	3 prime UTR variant	-	-	-	-
rs148483119	11:3660015111:36600151	T/C	0.002 (C)	SNP	1000Genomes,	3 prime UTR variant	-	-	-	-
rs140971504	11:3660250411:36602504	C/T	0.001 (T)	SNP	1000Genomes,	Downstream gene variant	-	-	-	-
rs138870142	11:3660273811:36602738	A/T	0.002 (T)	SNP	1000Genomes,	Downstream gene variant	-	-	-	-
rs149442029	11:3660275811:36602758	G/A	0.008 (A)	SNP	1000Genomes,	Downstream gene variant	-	-	-	-
rs142337839	11:3660328411:36603284	T/A	0.004 (A)	SNP	1000Genomes,	Downstream gene variant	-	-	-	-
rs145930516	11:3660344511:36603445	C/G	0.001 (G)	SNP	1000Genomes,	Downstream gene variant	-	-	-	-
rs139878501	11:3660394511:36603945	C/T	0.004 (T)	SNP	1000Genomes,	Downstream gene variant	-	-	-	-
rs76511123	11:3660417911:36604179	A/T	0.001 (T)	SNP	1000Genomes,	Downstream gene variant	-	-	-	-
rs139141414	11:3660420411:36604204	C/T	0.002 (T)	SNP	1000Genomes,	Downstream gene variant	-	-	-	-
rs139907436	11:3660441011:36604410	G/T	0.009 (T)	SNP	1000Genomes,	Downstream gene variant	-	-	-	-
rs147659805	11:3660484511:36604845	C/T	0.004 (T)	SNP	1000Genomes,	Downstream gene variant	-	-	-	-
rs149165412	11:3660520811:36605208	T/C	0.001 (C)	SNP	1000Genomes,	Downstream gene variant	-	-	-	-
rs143285432	11:3660522611:36605226	C/T	0.001 (T)	SNP	1000Genomes,	Downstream gene variant	-	-	-	-
rs140539498	11:3660598211:36605982	A/C	0.001 (C)	SNP	1000Genomes,	Downstream gene variant	-	-	-	-
rs79486620	11:3660608711:36606087	A/G	0.005 (G)	SNP	1000Genomes,	Downstream gene variant	-	-	-	-
rs138489163	11:3660620511:36606205	G/A	0.005 (A)	SNP	1000Genomes,	Downstream gene variant	-	-	-	-
rs187301762	11:3658471211:36584712	G/T	0.001 (T)	SNP	-	Upstream gene variant	-	-	-	-
rs191748582	11:3658523711:36585237	G/A	0.003 (A)	SNP	-	Upstream gene variant	-	-	-	-
rs184525443	11:3658537811:36585378	T/C	0.002 (C)	SNP	-	Upstream gene variant	-	-	-	-
rs150102929	11:3658548111:36585481	A/G	0.001 (G)	SNP	-	Upstream gene variant	-	-	-	-
rs189260790	11:3658600911:36586009	T/G	0.001 (G)	SNP	-	Upstream gene variant	-	-	-	-
rs182154807	11:3658607711:36586077	A/G	0.001 (G)	SNP	-	Upstream gene variant	-	-	-	-
rs185543806	11:3658630011:36586300	C/T	0.001 (T)	SNP	-	Upstream gene variant	-	-	-	-
rs189029926	11:3658634011:36586340	C/T	0.001 (T)	SNP	-	Upstream gene variant	-	-	-	-
rs143939152	11:3658648511:36586485-36586491	CGTTGTC/-	0.006 (-)	deletion	-	Upstream gene variant	-	-	-	-
rs181637298	11:3658685211:36586852	G/T	0.001 (T)	SNP	-	Upstream gene variant	-	-	-	-
rs150640980	11:3658700711:36587007	T/A	0.002 (A)	SNP	-	Upstream gene variant	-	-	-	-
rs185816377	11:3658780711:36587807	G/A	0.001 (A)	SNP	-	Upstream gene variant	-	-	-	-
rs190278950	11:3658780811:36587808	C/A	0.001 (A)	SNP	-	Upstream gene variant	-	-	-	-
rs151163578	11:3658886411:36588864	C/A	0.005 (A)	SNP	-	Upstream gene variant	-	-	-	-
rs185970602	11:3658893411:36588934	A/G	0.001 (G)	SNP	-	Upstream gene variant	-	-	-	-
rs183964566	11:3658912311:36589123	A/G	0.002 (G)	SNP	-	Upstream gene variant	-	-	-	-
rs189163766	11:3658916711:36589167	G/A	0.001 (A)	SNP	-	Upstream gene variant	-	-	-	-
rs190968516	11:3658968411:36589684	A/G	0.001 (G)	SNP	-	Intron variant	-	-	-	-
rs183176795	11:3658978011:36589780	A/G	0.001 (G)	SNP	-	Intron variant	-	-	-	-
rs187498735	11:3659007211:36590072	T/C	0.001 (C)	SNP	-	Intron variant	-	-	-	-
rs191783409	11:3659048111:36590481	T/C	0.001 (C)	SNP	-	Intron variant	-	-	-	-
rs200252068	11:3659048411:36590484	C/A	-	SNP	-	Intron variant	-	-	-	-
rs183093085	11:3659052311:36590523	G/A	0.002 (A)	SNP	-	Intron variant	-	-	-	-
rs187642373	11:3659067811:36590678	T/C	0.001 (C)	SNP	-	Intron variant	-	-	-	-
rs142254848	11:3659068411:36590684-36590686	GCT/-	-	deletion	-	Intron variant, Feature truncation	-	-	-	-
rs192626550	11:3659078811:36590788	G/C	0.001 (C)	SNP	-	Intron variant	-	-	-	-
rs200979608	11:3659112311:36591123-36591124	AA/-	-	deletion	-	Intron variant, Feature truncation	-	-	-	-

rs201800589	11:3659128011:36591280-36591282	TAA/-	0.012 (-)	deletion	-	Intron variant, Feature truncation	-	-	-	-
rs141557519	11:3659128111:36591281-36591285	AATAA/-	0.012 (-)	deletion	-	Intron variant, Feature truncation	-	-	-	-
rs184240786	11:3659128911:36591289	A/G	0.005 (G)	SNP	-	Intron variant	-	-	-	-
rs189467851	11:3659138611:36591386	G/A	0.001 (A)	SNP	-	Intron variant	-	-	-	-
rs192467736	11:3659143411:36591434	A/G	0.001 (G)	SNP	-	Intron variant	-	-	-	-
rs184503790	11:3659147411:36591474	G/A	0.001 (A)	SNP	-	Intron variant	-	-	-	-
rs188757378	11:3659150011:36591500	C/G	0.001 (G)	SNP	-	Intron variant	-	-	-	-
rs11329058	11:3659170711:36591707	T/-	-	deletion	-	Intron variant, Feature truncation	-	-	-	-
rs144137229	11:3659175111:36591751	A/G	0.000 (G)	SNP	-	Intron variant	-	-	-	-
rs189475567	11:3659178011:36591780	G/T	0.001 (T)	SNP	-	Intron variant	-	-	-	-
rs201395382	11:3659190311:36591903	T/C	-	SNP	-	Intron variant	-	-	-	-
rs112440348	11:3659240011:36592400	G/C	-	SNP	-	Intron variant	-	-	-	-
rs79295607	11:3659245411:36592454	T/C	-	SNP	-	Intron variant	-	-	-	-
rs76603665	11:3659248211:36592482	G/A	-	SNP	-	Intron variant	-	-	-	-
rs200068995	11:3659248911:36592489	G/A	-	SNP	-	Intron variant	-	-	-	-
rs55679809	11:3659274411:36592744	C/T	-	SNP	-	Intron variant	-	-	-	-
rs181022935	11:3659298911:36592989	A/G	0.001 (G)	SNP	-	Intron variant	-	-	-	-
rs185265982	11:3659300911:36593009	C/T	0.001 (T)	SNP	-	Intron variant	-	-	-	-
rs183216792	11:3659301511:36593015	G/C	0.005 (C)	SNP	-	Intron variant	-	-	-	-
rs186126515	11:3659321811:36593218	G/A	0.003 (A)	SNP	-	Intron variant	-	-	-	-
rs191690982	11:3659335311:36593353	A/T	0.001 (T)	SNP	-	Intron variant	-	-	-	-
rs188380538	11:3659344811:36593448	A/G	0.001 (G)	SNP	-	Intron variant	-	-	-	-
rs183012850	11:3659352611:36593526	C/T	0.001 (T)	SNP	-	Intron variant	-	-	-	-
rs186641408	11:3659352711:36593527	G/A	0.001 (A)	SNP	-	Intron variant	-	-	-	-
rs142774263	11:3659379811:36593798	A/-	-	deletion	-	Intron variant, Feature truncation	-	-	-	-
rs184560088	11:3659392211:36593922	G/A	0.002 (A)	SNP	-	Intron variant	-	-	-	-
rs189005815	11:3659392711:36593927	T/C	0.001 (C)	SNP	-	Intron variant	-	-	-	-
rs185100010	11:3659402211:36594022	C/T	0.001 (T)	SNP	-	Intron variant	-	-	-	-
rs35678890	11:3659433211: between 36594332 & 36594333	-/C	-	insertion	-	Feature elongation, Intron variant	-	-	-	-
rs189996292	11:3659443311:36594433	G/C	0.001 (C)	SNP	-	Intron variant	-	-	-	-
rs151218623	11:3659452811:36594528	T/A	0.001 (A)	SNP	-	Intron variant	-	-	-	-
rs201501543	11:3659481411:36594814	A/C	0.001 (C)	SNP	-	Intron variant	-	-	-	-
COSM231244	11:3659492111:36594921	A/T	-	somatic_S	-	Missense variant	I/F	23	31deleterious(0.03)	629possibly damaging(0.628)
COSM339567	11:3659493111:36594931	C/A	-	somatic_S	-	Stop gained	S/*	26	-	-
COSM1353713	11:3659495411:36594954	C/T	-	somatic_S	-	Missense variant	R/W	34	1deleterious(0)	910probably damaging(0.909)
COSM368513	11:3659495511:36594955	G/T	-	somatic_S	-	Missense variant	R/L	34	1deleterious(0)	760possibly damaging(0.759)
COSM1507897	11:3659496111:36594961	G/T	-	somatic_S	-	Missense variant	R/I	36	1deleterious(0)	867possibly damaging(0.866)
COSM186659	11:3659497911:36594979	C/A	-	somatic_S	-	Missense variant	P/H	42	1deleterious(0)	17benign(0.016)
COSM1507896	11:3659499811:36594998	A/T	-	somatic_S	-	Missense variant	E/D	48	381tolerated(0.38)	4benign(0.003)
COSM1298010	11:3659500111:36595001	G/A	-	somatic_S	-	Synonymous variant	K	49	-	-
COSM257851	11:3659500111:36595001	G/T	-	somatic_S	-	Missense variant	K/N	49	781tolerated(0.78)	424benign(0.423)
rs201625282	11:3659504011:36595040	T/G	-	SNP	-	Synonymous variant	S	62	-	-
COSM1287527	11:3659509411:36595094	G/T	-	somatic_S	-	Missense variant	L/F	80	71tolerated(0.07)	2benign(0.001)
CD065781	11:3659510811:36595108	HGMD_MUTA	-	deletion	-	Coding sequence variant		85	-	-
RAG1base_RAG1_DNA:g.65	11:3659510811:36595108-36595110	CTA/-	-	deletion	-	Feature truncation, Missense varian	PK/Q	85	-	-
CD982919	11:3659511011:36595110	HGMD_MUTA	-	deletion	-	Coding sequence variant		86	-	-
RAG1base_HSRAG1:g.368_3	11:3659511011:36595110-36595111	AA/-	-	deletion	-	Frameshift variant, Feature truncation		86	-	-
RAG1base_RAG1_DNA:g.65	11:3659511011:36595110-36595111	AA/-	-	deletion	-	Frameshift variant, Feature truncation		86	-	-
COSM1507895	11:3659513111:36595131	G/C	-	somatic_S	-	Missense variant	D/H	93	251tolerated(0.25)	64benign(0.063)
COSM926729	11:3659513611:36595136	C/T	-	somatic_S	-	Synonymous variant	N	94	-	-
COSM136829	11:3659518811:36595188	C/T	-	somatic_S	-	Missense variant	R/C	112	1deleterious(0)	910probably damaging(0.909)
COSM317943	11:3659522011:36595220	G/C	-	somatic_S	-	Missense variant	E/D	122	501tolerated(0.5)	2benign(0.001)
CM065423	11:3659526011:36595260	HGMD_MUTA	-	SNP	-	Coding sequence variant		136	-	-
C;vf=60925611;source=Pher	11:3659526011:36595260	A/C	-	SNP	-	Missense variant	K/Q	136	61tolerated(0.06)	825possibly damaging(0.824)
CM067457	11:3659527811:36595278	HGMD_MUTA	-	SNP	-	Coding sequence variant		142	-	-
T;vf=60925612;source=Pher	11:3659527811:36595278	C/T	-	SNP	-	Stop gained	R/*	142	-	-
COSM376240	11:3659530511:36595305	T/C	-	somatic_S	-	Missense variant	W/R	151	1deleterious(0)	913probably damaging(0.912)
COSM343989	11:3659536111:36595361	G/C	-	somatic_S	-	Synonymous variant	S	169	-	-
COSM171937	11:3659536111:36595361	G/A	-	somatic_S	-	Synonymous variant	S	169	-	-
CD003351	11:3659537311:36595373	HGMD_MUTA	-	deletion	-	Coding sequence variant		173	-	-

RAG1base_HSRAG1:g.631de	11:3659537311:36595373	T/-	-	deletion	-	Frameshift variant, Feature truncation		173	-	-
RAG1base_RAG1_DNA:g.68	11:3659537311:36595373	T/-	-	deletion	-	Frameshift variant, Feature truncation		173	-	-
COSM1353714	11:3659541711:36595417	G/A	-	somatic_S	-	Missense variant	S/N	188	161tolerated(0.16)	511possibly damaging(0.51)
CM065427	11:3659542911:36595429	HGMD_MUTA	-	SNP	-	Coding sequence variant		192	-	-
A;vf=60925614;source=Pher	11:3659542911:36595429	G/A	-	SNP	-	Missense variant	C/Y	192	71tolerated(0.07)	5benign(0.004)
COSM1353715	11:3659545111:36595451	C/T	-	somatic_S	-	Synonymous variant	N	199	-	-
rs200182366	11:3659547211:36595472	C/A	0.004 (A)	SNP	-	Synonymous variant	P	206	-	-
COSM108879	11:3659548311:36595483	C/T	-	somatic_S	-	Missense variant	S/F	210	191tolerated(0.19)	27benign(0.026)
COSM396370	11:3659551111:36595511	G/A	-	somatic_S	-	Synonymous variant	R	219	-	-
COSM1263746	11:3659556211:36595562	C/A	-	somatic_S	-	Synonymous variant	L	236	-	-
rs146046446	11:3659557311:36595573	T/-	-	deletion	-	Frameshift variant, Feature truncation		240	-	-
COSM223454	11:3659558111:36595581	G/A	-	somatic_S	-	Missense variant	A/T	243	181tolerated(0.18)	847possibly damaging(0.846)
rs199474683	11:3659558411:36595584	A/G	-	SNP	-	Missense variant	R/G	244	41deleterious(0.04)	3benign(0.002)
COSM1507894	11:3659559311:36595593	C/T	-	somatic_S	-	Missense variant	R/C	247	51tolerated(0.05)	1benign(0)
CM065424	11:3659559611:36595596	HGMD_MUTA	-	SNP	-	Coding sequence variant		248	-	-
T;vf=60925615;source=Pher	11:3659559611:36595596	C/T	-	SNP	-	Stop gained	Q/*	248	-	-
RAG1base_RAG1_DNA:g.70	11:3659561911:36595619	A/-	-	deletion	-	Frameshift variant, Feature truncation		255	-	-
CD003352	11:3659562911:36595629	HGMD_MUTA	-	deletion	-	Coding sequence variant		259	-	-
RAG1base_HSRAG1:g.887de	11:3659562911:36595629	A/-	-	deletion	-	Frameshift variant, Feature truncation		259	-	-
COSM336373	11:3659564111:36595641	A/T	-	somatic_S	-	Missense variant	M/L	263	131tolerated(0.13)	1benign(0)
rs188125314	11:3659573311:36595733	C/T	0.000 (T)	SNP	-	Synonymous variant	C	293	-	-
CD056838	11:3659574111:36595741	HGMD_MUTA	-	deletion	-	Coding sequence variant		296	-	-
rs121918568	11:3659579411:36595794	C/T	-	SNP	-	Missense variant	R/W	314	1deleterious(0)	963probably damaging(0.962)
CM081766	11:3659579411:36595794	HGMD_MUTA	-	SNP	-	Coding sequence variant		314	-	-
T;vf=60925617;source=Pher	11:3659579411:36595794	C/T	-	SNP	-	Missense variant	R/W	314	1deleterious(0)	963probably damaging(0.962)
rs121918571	11:3659583711:36595837	G/A	-	SNP	-	Missense variant	C/Y	328	1deleterious(0)	993probably damaging(0.992)
CM010066	11:3659583711:36595837	HGMD_MUTA	-	SNP	-	Coding sequence variant		328	-	-
A;vf=60925567;source=Pher	11:3659583711:36595837	G/A	-	SNP	-	Missense variant	C/Y	328	1deleterious(0)	993probably damaging(0.992)
rs181967562	11:3659583911:36595839	C/T	0.000 (T)	SNP	-	Missense variant	P/S	329	1deleterious(0)	953probably damaging(0.952)
CM016162	11:3659585311:36595853	HGMD_MUTA	-	SNP	-	Coding sequence variant		333	-	-
A;vf=60925618;source=Pher	11:3659585311:36595853	A/T	-	SNP	-	Stop gained	Y/*	333	-	-
COSM428954	11:3659587011:36595870	A/T	-	somatic_S	-	Missense variant	D/V	339	1deleterious(0)	893possibly damaging(0.892)
rs200758244	11:3659590211:36595902	G/A	0.001 (A)	SNP	-	Missense variant	V/I	350	1001tolerated(1)	1benign(0)
CD010112	11:3659591511:36595915	HGMD_MUTA	-	deletion	-	Coding sequence variant		354	-	-
RAG1base_HSRAG1:g.1173d	11:3659591511:36595915	T/-	-	deletion	-	Frameshift variant, Feature truncation		354	-	-
COSM1317316	11:3659591511:36595915	T/G	-	somatic_S	-	Missense variant	L/R	354	1deleterious(0)	989probably damaging(0.988)
COSM1507893	11:3659591511:36595915	T/A	-	somatic_S	-	Missense variant	L/Q	354	1deleterious(0)	989probably damaging(0.988)
rs186342720	11:3659594511:36595945	A/G	0.001 (G)	SNP	-	Missense variant	N/S	364	291tolerated(0.29)	1benign(0)
COSM926732	11:3659598211:36595982	C/A	-	somatic_S	-	Synonymous variant	I	376	-	-
RAG1base_HSRAG1:g.1258d	11:3659600011:36596000	A/-	-	deletion	-	Frameshift variant, Feature truncation		382	-	-
CD010113	11:3659600311:36596003	HGMD_MUTA	-	deletion	-	Coding sequence variant		383	-	-
CM065417	11:3659603411:36596034	HGMD_MUTA	-	SNP	-	Coding sequence variant		394	-	-
T;vf=60925619;source=Pher	11:3659603411:36596034	C/T	-	SNP	-	Missense variant	R/W	394	1deleterious(0)	910probably damaging(0.909)
CM981692	11:3659604011:36596040	HGMD_MUTA	-	SNP	-	Coding sequence variant		396	-	-
T;vf=60925570;source=Pher	11:3659604011:36596040	C/T	-	SNP	-	Missense variant	R/C	396	1deleterious(0)	910probably damaging(0.909)
T;vf=60925620;source=Pher	11:3659604011:36596040	C/T	-	SNP	-	Missense variant	R/C	396	1deleterious(0)	910probably damaging(0.909)
CM010067	11:3659604111:36596041	HGMD_MUTA	-	SNP	-	Coding sequence variant		396	-	-
CM981691	11:3659604111:36596041	HGMD_MUTA	-	SNP	-	Coding sequence variant		396	-	-
A;vf=60925571;source=Pher	11:3659604111:36596041	G/A	-	SNP	-	Missense variant	R/H	396	1deleterious(0)	874possibly damaging(0.873)
T;vf=60925572;source=Pher	11:3659604111:36596041	G/T	-	SNP	-	Missense variant	R/L	396	1deleterious(0)	760possibly damaging(0.759)
A;vf=60925621;source=Pher	11:3659604111:36596041	G/A	-	SNP	-	Missense variant	R/H	396	1deleterious(0)	874possibly damaging(0.873)
COSM304336	11:3659604111:36596041	G/A	-	somatic_S	-	Missense variant	R/H	396	1deleterious(0)	874possibly damaging(0.873)
rs199474682	11:3659605511:36596055	T/C	-	SNP	-	Missense variant	S/P	401	1deleterious(0)	745possibly damaging(0.744)
CM010068	11:3659605511:36596055	HGMD_MUTA	-	SNP	-	Coding sequence variant		401	-	-
COSM1353716	11:3659605911:36596059	T/C	-	somatic_S	-	Missense variant	L/P	402	1deleterious(0)	913probably damaging(0.912)
CM016163	11:3659606411:36596064	HGMD_MUTA	-	SNP	-	Coding sequence variant		404	-	-
T;vf=60925622;source=Pher	11:3659606411:36596064	C/T	-	SNP	-	Missense variant	R/W	404	1deleterious(0)	910probably damaging(0.909)
CM065419	11:3659606511:36596065	HGMD_MUTA	-	SNP	-	Coding sequence variant		404	-	-
A;vf=60925623;source=Pher	11:3659606511:36596065	G/A	-	SNP	-	Missense variant	R/Q	404	1deleterious(0)	644possibly damaging(0.643)
rs199474684	11:3659608311:36596083	G/A	-	SNP	-	Missense variant	R/Q	410	1deleterious(0)	644possibly damaging(0.643)

CM010069	11:3659608311:36596083	HGMD_MUTA	-	SNP	-	Coding sequence variant		410	-	-
A;vf=60925573;source=Pher	11:3659608311:36596083	G/A	-	SNP	-	Missense variant	R/Q	410	1deleterious(0)	644possibly damaging(0.643)
CM981693	11:3659614011:36596140	HGMD_MUTA	-	SNP	-	Coding sequence variant		429	-	-
G;vf=60925574;source=Pher	11:3659614011:36596140	A/G	-	SNP	-	Missense variant	D/G	429	1deleterious(0)	847possibly damaging(0.846)
rs199474679	11:3659615111:36596151	G/A	-	SNP	-	Missense variant	V/M	433	11deleterious(0.01)	918probably damaging(0.917)
CM010070	11:3659615111:36596151	HGMD_MUTA	-	SNP	-	Coding sequence variant		433	-	-
A;vf=60925575;source=Pher	11:3659615111:36596151	G/A	-	SNP	-	Missense variant	V/M	433	11deleterious(0.01)	918probably damaging(0.917)
CM010071	11:3659615711:36596157	HGMD_MUTA	-	SNP	-	Coding sequence variant		435	-	-
G;vf=60925576;source=Pher	11:3659615711:36596157	A/G	-	SNP	-	Missense variant	M/V	435	1deleterious(0)	1benign(0)
rs199474685	11:3659618511:36596185	C/T	-	SNP	-	Missense variant	A/V	444	1deleterious(0)	855possibly damaging(0.854)
CM010072	11:3659618511:36596185	HGMD_MUTA	-	SNP	-	Coding sequence variant		444	-	-
T;vf=60925577;source=Pher	11:3659618511:36596185	C/T	-	SNP	-	Missense variant	A/V	444	1deleterious(0)	855possibly damaging(0.854)
CM065415	11:3659620011:36596200	HGMD_MUTA	-	SNP	-	Coding sequence variant		449	-	-
A;vf=60925624;source=Pher	11:3659620011:36596200	G/A	-	SNP	-	Missense variant	R/K	449	131tolerated(0.13)	393benign(0.392)
CM056040	11:3659620111:36596201	HGMD_MUTA	-	SNP	-	Coding sequence variant		449	-	-
rs199474677	11:3659621511:36596215	T/A	-	SNP	-	Missense variant	L/Q	454	1deleterious(0)	913probably damaging(0.912)
rs201779957	11:3659622111:36596221	C/A	-	SNP	-	Missense variant	A/D	456	1deleterious(0)	939probably damaging(0.938)
rs191898756	11:3659623311:36596233	G/C	0.001 (C)	SNP	-	Missense variant	G/A	460	11deleterious(0.01)	919probably damaging(0.918)
CD010114	11:3659626311:36596263	HGMD_MUTA	-	deletion	-	Coding sequence variant		470	-	-
RAG1base_HSRAG1:g.1521_	11:3659626311:36596263-36596264	GC/-	-	deletion	-	Frameshift variant, Feature truncation		470	-	-
CM065418	11:3659627411:36596274	HGMD_MUTA	-	SNP	-	Coding sequence variant		474	-	-
CM097360	11:3659627411:36596274	HGMD_MUTA	-	SNP	-	Coding sequence variant		474	-	-
A;vf=60925625;source=Pher	11:3659627411:36596274	C/A	-	SNP	-	Missense variant	R/S	474	1deleterious(0)	760possibly damaging(0.759)
rs199474686	11:3659627511:36596275	G/A	-	SNP	-	Missense variant	R/H	474	1deleterious(0)	874possibly damaging(0.873)
CM010073	11:3659627511:36596275	HGMD_MUTA	-	SNP	-	Coding sequence variant		474	-	-
A;vf=60925579;source=Pher	11:3659627511:36596275	G/A	-	SNP	-	Missense variant	R/H	474	1deleterious(0)	874possibly damaging(0.873)
A;vf=60925626;source=Pher	11:3659627511:36596275	G/A	-	SNP	-	Missense variant	R/H	474	1deleterious(0)	874possibly damaging(0.873)
COSM314667	11:3659634011:36596340	G/T	-	somatic_S	-	Missense variant	G/W	496	1deleterious(0)	985probably damaging(0.984)
COSM1353717	11:3659634411:36596344	G/A	-	somatic_S	-	Missense variant	R/K	497	111tolerated(0.11)	393benign(0.392)
rs104894298	11:3659637311:36596373	C/T	-	SNP	-	Missense variant	R/W	507	1deleterious(0)	910probably damaging(0.909)
CM010074	11:3659637311:36596373	HGMD_MUTA	-	SNP	-	Coding sequence variant		507	-	-
T;vf=60925580;source=Pher	11:3659637311:36596373	C/T	-	SNP	-	Missense variant	R/W	507	1deleterious(0)	910probably damaging(0.909)
T;vf=60925627;source=Pher	11:3659637311:36596373	C/T	-	SNP	-	Missense variant	R/W	507	1deleterious(0)	910probably damaging(0.909)
COSM186660	11:3659638411:36596384	G/A	-	somatic_S	-	Synonymous variant	E	510	-	-
COSM1263747	11:3659639811:36596398	C/T	-	somatic_S	-	Missense variant	P/L	515	1deleterious(0)	985probably damaging(0.984)
rs61758790	11:3659641411:36596414	T/G	-	SNP	-	Missense variant	F/L	520	11deleterious(0.01)	806possibly damaging(0.805)
CM010075	11:3659642011:36596420	HGMD_MUTA	-	SNP	-	Coding sequence variant		522	-	-
T;vf=60925581;source=Pher	11:3659642011:36596420	G/T	-	SNP	-	Missense variant	W/C	522	21deleterious(0.02)	985probably damaging(0.984)
COSM269995	11:3659642311:36596423	G/A	-	somatic_S	-	Synonymous variant	Q	523	-	-
COSM1507892	11:3659642811:36596428	C/A	-	somatic_S	-	Missense variant	P/H	525	11deleterious(0.01)	993probably damaging(0.992)
COSM1263748	11:3659642811:36596428	C/T	-	somatic_S	-	Missense variant	P/L	525	31deleterious(0.03)	985probably damaging(0.984)
RAG1base_HSRAG1:g.1723_	11:3659646511:36596465-36596477	TATTGATGGG	-	deletion	-	Frameshift variant, Feature truncation		537	-	-
CD982920	11:3659646611:36596466	HGMD_MUTA	-	deletion	-	Coding sequence variant		538	-	-
rs17853743	11:3659648311:36596483	A/G	-	SNP	-	Synonymous variant	G	543	-	-
COSM352369	11:3659649611:36596496	G/T	-	somatic_S	-	Missense variant	V/L	548	91tolerated(0.09)	805possibly damaging(0.804)
COSM167756	11:3659651411:36596514	G/A	-	somatic_S	-	Missense variant	D/N	554	41deleterious(0.04)	957probably damaging(0.956)
rs199474681	11:3659653111:36596531	G/T	-	SNP	-	Missense variant	R/S	559	1deleterious(0)	871possibly damaging(0.87)
CM010076	11:3659653111:36596531	HGMD_MUTA	-	SNP	-	Coding sequence variant		559	-	-
T;vf=60925583;source=Pher	11:3659653111:36596531	G/T	-	SNP	-	Missense variant	R/S	559	1deleterious(0)	871possibly damaging(0.87)
T;vf=60925628;source=Pher	11:3659653111:36596531	G/T	-	SNP	-	Missense variant	R/S	559	1deleterious(0)	871possibly damaging(0.87)
CM981695	11:3659653511:36596535	HGMD_MUTA	-	SNP	-	Coding sequence variant		561	-	-
T;vf=60925584;source=Pher	11:3659653511:36596535	C/T	-	SNP	-	Missense variant	R/C	561	1deleterious(0)	976probably damaging(0.975)
T;vf=60925629;source=Pher	11:3659653511:36596535	C/T	-	SNP	-	Missense variant	R/C	561	1deleterious(0)	976probably damaging(0.975)
COSM415610	11:3659653511:36596535	C/A	-	somatic_S	-	Missense variant	R/S	561	1deleterious(0)	927probably damaging(0.926)
CM981694	11:3659653611:36596536	HGMD_MUTA	-	SNP	-	Coding sequence variant		561	-	-
A;vf=60925585;source=Pher	11:3659653611:36596536	G/A	-	SNP	-	Missense variant	R/H	561	1deleterious(0)	965probably damaging(0.964)
A;vf=60925630;source=Pher	11:3659653611:36596536	G/A	-	SNP	-	Missense variant	R/H	561	1deleterious(0)	965probably damaging(0.964)
COSM1507891	11:3659654111:36596541	G/T	-	somatic_S	-	Missense variant	D/Y	563	1deleterious(0)	993probably damaging(0.992)
A;vf=60925631;source=Pher	11:3659654811:36596548	C/A	-	SNP	-	Missense variant	A/D	565	1deleterious(0)	985probably damaging(0.984)
COSM186661	11:3659658011:36596580	G/T	-	somatic_S	-	Missense variant	D/Y	576	1deleterious(0)	993probably damaging(0.992)

COSM1475405	11:3659658511:36596585	C/T	-	somatic_S	-	Synonymous variant	I	577	-	-
COSM163910	11:3659659711:36596597	G/C	-	somatic_S	-	Missense variant	M/I	581	11deleterious(0.01)	455possibly damaging(0.454)
COSM84060	11:3659660811:36596608	A/G	-	somatic_S	-	Missense variant	D/G	585	1001tolerated(1)	18benign(0.017)
CM010077	11:3659662111:36596621	HGMD_MUTA	-	SNP	-	Coding sequence variant		589	-	-
G;vf=60925586;source=Phen	11:3659662111:36596621	G/C	-	SNP	-	Stop gained	Y/*	589	-	-
G;vf=60925632;source=Phen	11:3659662111:36596621	G/C	-	SNP	-	Stop gained	Y/*	589	-	-
COSM1507890	11:3659665511:36596655	T/C	-	somatic_S	-	Missense variant	S/P	601	1deleterious(0)	921probably damaging(0.92)
CM065428	11:3659666011:36596660	HGMD_MUTA	-	SNP	-	Coding sequence variant		602	-	-
G;vf=60925633;source=Phen	11:3659666011:36596660	T/G	-	SNP	-	Missense variant	C/W	602	1deleterious(0)	975probably damaging(0.974)
COSM338957	11:3659666811:36596668	T/C	-	somatic_S	-	Missense variant	M/T	605	1deleterious(0)	575possibly damaging(0.574)
COSM1353718	11:3659667211:36596672	A/G	-	somatic_S	-	Synonymous variant	G	606	-	-
COSM13951	11:3659667211:36596672	A/T	-	somatic_S	-	Synonymous variant	G	606	-	-
rs183806098	11:3659667511:36596675	C/T	0.001 (T)	SNP	-	Synonymous variant	D	607	-	-
COSM394912	11:3659669311:36596693	G/T	-	somatic_S	-	Synonymous variant	G	613	-	-
COSM1353719	11:3659671611:36596716	A/G	-	somatic_S	-	Missense variant	K/R	621	11deleterious(0.01)	870possibly damaging(0.869)
COSM13915	11:3659671711:36596717	G/T	-	somatic_S	-	Missense variant	K/N	621	11deleterious(0.01)	950probably damaging(0.949)
CM065422	11:3659671811:36596718	HGMD_MUTA	-	SNP	-	Coding sequence variant		622	-	-
C;vf=60925634;source=Phen	11:3659671811:36596718	C/G	-	SNP	-	Missense variant	A/P	622	21deleterious(0.02)	985probably damaging(0.984)
CM010078	11:3659672411:36596724	HGMD_MUTA	-	SNP	-	Coding sequence variant		624	-	-
T;vf=60925587;source=Phen	11:3659672411:36596724	C/T	-	SNP	-	Missense variant	R/C	624	1deleterious(0)	976probably damaging(0.975)
T;vf=60925635;source=Phen	11:3659672411:36596724	C/T	-	SNP	-	Missense variant	R/C	624	1deleterious(0)	976probably damaging(0.975)
rs199474680	11:3659672511:36596725	G/A	-	SNP	-	Missense variant	R/H	624	1deleterious(0)	965probably damaging(0.964)
CM961214	11:3659672511:36596725	HGMD_MUTA	-	SNP	-	Coding sequence variant		624	-	-
A;vf=60925588;source=Phen	11:3659672511:36596725	G/A	-	SNP	-	Missense variant	R/H	624	1deleterious(0)	965probably damaging(0.964)
A;vf=60925636;source=Phen	11:3659672511:36596725	G/A	-	SNP	-	Missense variant	R/H	624	1deleterious(0)	965probably damaging(0.964)
COSM138808	11:3659676411:36596764	G/A	-	somatic_S	-	Missense variant	S/N	637	411tolerated(0.41)	2benign(0.001)
rs144475142	11:3659677111:36596771	G/A	-	SNP	-	Synonymous variant	Q	639	-	-
CI003354	11:3659677911: between 36596779 & 36596780	HGMD_MUTA	-	insertion	-	Feature elongation, Coding sequence variant		642	-	-
RAG1base_HSRAG1:g.2038_	11:3659678011: between 36596780 & 36596781	-/PhenCode_v	-	sequence	-	Feature elongation, Coding sequence variant		643	-	-
COSM1353720	11:3659679011:36596790	G/T	-	somatic_S	-	Stop gained	E/*	646	-	-
CD065782	11:3659680111:36596801	HGMD_MUTA	-	deletion	-	Coding sequence variant		649	-	-
RAG1base_RAG1_DNA:g.823	11:3659680111:36596801	T/-	-	deletion	-	Frameshift variant, Feature truncation		649	-	-
COSM328851	11:3659680311:36596803	A/C	-	somatic_S	-	Missense variant	N/T	650	1deleterious(0)	916probably damaging(0.915)
CM065414	11:3659680411:36596804	HGMD_MUTA	-	SNP	-	Coding sequence variant		650	-	-
COSM1353721	11:3659683711:36596837	G/A	-	somatic_S	-	Missense variant	M/I	661	31deleterious(0.03)	455possibly damaging(0.454)
COSM186662	11:3659684711:36596847	G/T	-	somatic_S	-	Stop gained	E/*	665	-	-
CD057527	11:3659685611:36596856	HGMD_MUTA	-	deletion	-	Coding sequence variant		668	-	-
RAG1base_RAG1_DNA:g.829	11:3659685611:36596856	C/-	-	deletion	-	Frameshift variant, Feature truncation		668	-	-
rs199474689	11:3659686011:36596860	A/G	-	SNP	-	Missense variant	E/G	669	1deleterious(0)	916probably damaging(0.915)
CM010394	11:3659686011:36596860	HGMD_MUTA	-	SNP	-	Coding sequence variant		669	-	-
G;vf=60925590;source=Phen	11:3659686011:36596860	A/G	-	SNP	-	Missense variant	E/G	669	1deleterious(0)	916probably damaging(0.915)
COSM186663	11:3659686311:36596863	C/T	-	somatic_S	-	Missense variant	T/M	670	71tolerated(0.07)	986probably damaging(0.985)
RAG1base_HSRAG1:g.2182_	11:3659692411:36596924-36596931	AATGCTTG/-	-	deletion	-	Frameshift variant, Feature truncation		690	-	-
CD010115	11:3659692511:36596925	HGMD_MUTA	-	deletion	-	Coding sequence variant		691	-	-
rs199474676	11:3659694911:36596949	C/T	-	SNP	-	Missense variant	R/W	699	1deleterious(0)	976probably damaging(0.975)
COSM287899	11:3659694911:36596949	C/T	-	somatic_S	-	Missense variant	R/W	699	1deleterious(0)	976probably damaging(0.975)
COSM363644	11:3659695111:36596951	G/T	-	somatic_S	-	Synonymous variant	R	699	-	-
COSM1492530	11:3659697911:36596979	G/T	-	somatic_S	-	Missense variant	G/C	709	1deleterious(0)	999probably damaging(0.998)
CM065421	11:3659698011:36596980	HGMD_MUTA	-	SNP	-	Coding sequence variant		709	-	-
A;vf=60925639;source=Phen	11:3659698011:36596980	G/A	-	SNP	-	Missense variant	G/D	709	1deleterious(0)	995probably damaging(0.994)
COSM322973	11:3659699711:36596997	G/T	-	somatic_S	-	Missense variant	V/L	715	11deleterious(0.01)	805possibly damaging(0.804)
rs199776076	11:3659700011:36597000	C/T	-	SNP	-	Missense variant	R/W	716	1deleterious(0)	976probably damaging(0.975)
CM065420	11:3659700011:36597000	HGMD_MUTA	-	SNP	-	Coding sequence variant		716	-	-
T;vf=60925640;source=Phen	11:3659700011:36597000	C/T	-	SNP	-	Missense variant	R/W	716	1deleterious(0)	976probably damaging(0.975)
CM961215	11:3659701811:36597018	HGMD_MUTA	-	SNP	-	Coding sequence variant		722	-	-
A;vf=60925592;source=Phen	11:3659701811:36597018	G/A	-	SNP	-	Missense variant	E/K	722	21deleterious(0.02)	877possibly damaging(0.876)
rs201192233	11:3659703911:36597039	A/C	0.001 (C)	SNP	-	Missense variant	I/L	729	1deleterious(0)	523possibly damaging(0.522)
CM016165	11:3659704311:36597043	HGMD_MUTA	-	SNP	-	Coding sequence variant		730	-	-
T;vf=60925641;source=Phen	11:3659704311:36597043	G/T	-	SNP	-	Missense variant	C/F	730	21deleterious(0.02)	963probably damaging(0.962)
CM016164	11:3659704811:36597048	HGMD_MUTA	-	SNP	-	Coding sequence variant		732	-	-

T;vf=60925642;source=Pher	11:3659704811:36597048	C/T	-	SNP	-	Missense variant	L/F	732	1deleterious(0)	950probably damaging(0.949)
CM981696	11:3659706411:36597064	HGMD_MUTA	-	SNP	-	Coding sequence variant		737	-	-
A;vf=60925593;source=Pher	11:3659706411:36597064	G/A	-	SNP	-	Missense variant	R/H	737	1deleterious(0)	965probably damaging(0.964)
A;vf=60925643;source=Pher	11:3659706411:36597064	G/A	-	SNP	-	Missense variant	R/H	737	1deleterious(0)	965probably damaging(0.964)
COSM1470480	11:3659706411:36597064	G/A	-	somatic_S	-	Missense variant	R/H	737	1deleterious(0)	965probably damaging(0.964)
COSM926739	11:3659707211:36597072	G/T	-	somatic_S	-	Missense variant	A/S	740	11deleterious(0.01)	932probably damaging(0.931)
rs199474687	11:3659711211:36597112	A/T	-	SNP	-	Missense variant	H/L	753	1deleterious(0)	871possibly damaging(0.87)
CM010079	11:3659711211:36597112	HGMD_MUTA	-	SNP	-	Coding sequence variant		753	-	-
T;vf=60925594;source=Pher	11:3659711211:36597112	T/A	-	SNP	-	Missense variant	H/L	753	1deleterious(0)	871possibly damaging(0.87)
COSM145840	11:3659712911:36597129	C/T	-	somatic_S	-	Missense variant	R/C	759	11deleterious(0.01)	976probably damaging(0.975)
CM961216	11:3659717411:36597174	HGMD_MUTA	-	SNP	-	Coding sequence variant		774	-	-
T;vf=60925595;source=Pher	11:3659717411:36597174	G/T	-	SNP	-	Stop gained	E/*	774	-	-
rs121918572	11:3659718011:36597180	C/T	-	SNP	-	Missense variant	R/W	776	1deleterious(0)	976probably damaging(0.975)
CM090373	11:3659718011:36597180	HGMD_MUTA	-	SNP	-	Coding sequence variant		776	-	-
COSM355977	11:3659718011:36597180	C/T	-	somatic_S	-	Missense variant	R/W	776	1deleterious(0)	976probably damaging(0.975)
CM099821	11:3659718111:36597181	HGMD_MUTA	-	SNP	-	Coding sequence variant		776	-	-
A;vf=60925644;source=Pher	11:3659718111:36597181	G/A	-	SNP	-	Missense variant	R/Q	776	1deleterious(0)	878possibly damaging(0.877)
COSM386382	11:3659718111:36597181	G/T	-	somatic_S	-	Missense variant	R/L	776	1deleterious(0)	927probably damaging(0.926)
CM065425	11:3659718611:36597186	HGMD_MUTA	-	SNP	-	Coding sequence variant		778	-	-
CM065426	11:3659718611:36597186	HGMD_MUTA	-	SNP	-	Coding sequence variant		778	-	-
G;vf=60925645;source=Pher	11:3659718611:36597186	G/C	-	SNP	-	Missense variant	R/G	778	1deleterious(0)	927probably damaging(0.926)
T;vf=60925646;source=Pher	11:3659718611:36597186	C/T	-	SNP	-	Missense variant	R/W	778	1deleterious(0)	976probably damaging(0.975)
rs121918569	11:3659718711:36597187	G/A	-	SNP	-	Missense variant	R/Q	778	21deleterious(0.02)	878possibly damaging(0.877)
CM081768	11:3659718711:36597187	HGMD_MUTA	-	SNP	-	Coding sequence variant		778	-	-
A;vf=60925647;source=Pher	11:3659718711:36597187	G/A	-	SNP	-	Missense variant	R/Q	778	21deleterious(0.02)	878possibly damaging(0.877)
COSM1168721	11:3659718711:36597187	G/A	-	somatic_S	-	Missense variant	R/Q	778	21deleterious(0.02)	878possibly damaging(0.877)
COSM1507889	11:3659719411:36597194	A/T	-	somatic_S	-	Missense variant	K/N	780	1deleterious(0)	950probably damaging(0.949)
COSM1353722	11:3659719711:36597197	G/C	-	somatic_S	-	Synonymous variant	G	781	-	-
rs200300629	11:3659719911:36597199	T/A	-	SNP	-	Missense variant	V/D	782	1deleterious(0)	977probably damaging(0.976)
rs61752933	11:3659728211:36597282	A/G	-	SNP	-	Missense variant	I/V	810	11deleterious(0.01)	523possibly damaging(0.522)
CM068079	11:3659731311:36597313	HGMD_MUTA	-	SNP	-	Coding sequence variant		820	-	-
COSM428955	11:3659733311:36597333	G/C	-	somatic_S	-	Missense variant	E/Q	827	1deleterious(0)	916probably damaging(0.915)
CP005308	11:3659734111:36597341	HGMD_MUTA	-	indel	-	Coding sequence variant		829	-	-
RAG1base_RAG1_DNA:g.87	11:3659734111:36597341-36597342	GA/TT	-	substituti	-	Stop gained	RK/S*	829	-	-
CM010080	11:3659737511:36597375	HGMD_MUTA	-	SNP	-	Coding sequence variant		841	-	-
T;vf=60925596;source=Pher	11:3659737511:36597375	C/T	-	SNP	-	Missense variant	R/W	841	1deleterious(0)	976probably damaging(0.975)
T;vf=60925649;source=Pher	11:3659737511:36597375	C/T	-	SNP	-	Missense variant	R/W	841	1deleterious(0)	976probably damaging(0.975)
COSM1507888	11:3659739511:36597395	A/G	-	somatic_S	-	Synonymous variant	K	847	-	-
COSM369807	11:3659739811:36597398	A/T	-	somatic_S	-	Synonymous variant	P	848	-	-
rs199474690	11:3659741811:36597418	A/T	-	SNP	-	Missense variant	N/I	855	11deleterious(0.01)	977probably damaging(0.976)
CM010081	11:3659741811:36597418	HGMD_MUTA	-	SNP	-	Coding sequence variant		855	-	-
T;vf=60925597;source=Pher	11:3659741811:36597418	T/A	-	SNP	-	Missense variant	N/I	855	11deleterious(0.01)	977probably damaging(0.976)
COSM1127793	11:3659745011:36597450	G/A	-	somatic_S	-	Missense variant	V/M	866	41deleterious(0.04)	977probably damaging(0.976)
COSM428956	11:3659745311:36597453	G/A	-	somatic_S	-	Missense variant	D/N	867	41deleterious(0.04)	2benign(0.001)
rs193922462	11:3659745711:36597457	C/T	-	SNP	-	Missense variant	A/V	868	701tolerated(0.7)	957probably damaging(0.956)
rs201313833	11:3659745911:36597459	G/A	-	SNP	-	Missense variant	V/I	869	21deleterious(0.02)	805possibly damaging(0.804)
CM010082	11:3659746911:36597469	HGMD_MUTA	-	SNP	-	Coding sequence variant		872	-	-
A;vf=60925598;source=Pher	11:3659746911:36597469	A/T	-	SNP	-	Stop gained	L/*	872	-	-
CD016167	11:3659747711:36597477	HGMD_MUTA	-	deletion	-	Coding sequence variant		875	-	-
RAG1base_RAG1_DNA:g.89	11:3659747711:36597477	T/-	-	deletion	-	Frameshift variant, Feature truncation		875	-	-
COSM1470479	11:3659748011:36597480	G/A	-	somatic_S	-	Missense variant	E/K	876	31deleterious(0.03)	1benign(0)
COSM377837	11:3659748711:36597487-36597488	GG/TC	-	somatic_s	-	Missense variant	R/I	878	1deleterious(0)	965probably damaging(0.964)
rs199474691	11:3659750811:36597508	T/G	-	SNP	-	Missense variant	L/R	885	1deleterious(0)	977probably damaging(0.976)
CM002095	11:3659750811:36597508	HGMD_MUTA	-	SNP	-	Coding sequence variant		885	-	-
G;vf=60925599;source=Pher	11:3659750811:36597508	T/G	-	SNP	-	Missense variant	L/R	885	1deleterious(0)	977probably damaging(0.976)
G;vf=60925651;source=Pher	11:3659750811:36597508	T/G	-	SNP	-	Missense variant	L/R	885	1deleterious(0)	977probably damaging(0.976)
CM056379	11:3659754011:36597540	HGMD_MUTA	-	SNP	-	Coding sequence variant		896	-	-
C;vf=60925652;source=Pher	11:3659754011:36597540	T/C	-	SNP	-	Missense variant	W/R	896	1deleterious(0)	977probably damaging(0.976)
CM961217	11:3659754311:36597543	HGMD_MUTA	-	SNP	-	Coding sequence variant		897	-	-
T;vf=60925600;source=Pher	11:3659754311:36597543	C/T	-	SNP	-	Stop gained	R/*	897	-	-

T;vf=60925653;source=Pher	11:3659754311:36597543	C/T	-	SNP	-	Stop gained	R/*	897	-	-
COSM276989	11:3659754411:36597544	G/A	-	somatic_S	-	Missense variant	R/Q	897	1deleterious(0)	878possibly damaging(0.877)
CM010395	11:3659755411:36597554	HGMD_MUTA	-	SNP	-	Coding sequence variant		900	-	-
A;vf=60925601;source=Pher	11:3659755411:36597554	C/A	-	SNP	-	Stop gained	C/*	900	-	-
CM981697	11:3659758911:36597589	HGMD_MUTA	-	SNP	-	Coding sequence variant		912	-	-
G;vf=60925602;source=Pher	11:3659758911:36597589	A/G	-	SNP	-	Missense variant	Y/C	912	1deleterious(0)	985probably damaging(0.984)
COSM314666	11:3659758911:36597589	A/T	-	somatic_S	-	Missense variant	Y/F	912	31deleterious(0.03)	870possibly damaging(0.869)
COSM284453	11:3659763111:36597631	A/C	-	somatic_S	-	Missense variant	K/T	926	911tolerated(0.91)	916probably damaging(0.915)
CM961218	11:3659766811:36597668	HGMD_MUTA	-	SNP	-	Coding sequence variant		938	-	-
G;vf=60925603;source=Pher	11:3659766811:36597668	T/G	-	SNP	-	Stop gained	Y/*	938	-	-
rs182385524	11:3659772111:36597721	T/C	0.001 (C)	SNP	-	Missense variant	I/T	956	1deleterious(0)	629possibly damaging(0.628)
CM065416	11:3659772111:36597721	HGMD_MUTA	-	SNP	-	Coding sequence variant		956	-	-
C;vf=60925654;source=Pher	11:3659772111:36597721	T/C	-	SNP	-	Missense variant	I/T	956	1deleterious(0)	629possibly damaging(0.628)
CM010083	11:3659773011:36597730	HGMD_MUTA	-	SNP	-	Coding sequence variant		959	-	-
A;vf=60925604;source=Pher	11:3659773011:36597730	G/A	-	SNP	-	Stop gained	W/*	959	-	-
COSM257852	11:3659774711:36597747	G/T	-	somatic_S	-	Stop gained	E/*	965	-	-
COSM335997	11:3659774811:36597748	A/T	-	somatic_S	-	Missense variant	E/V	965	1deleterious(0)	825possibly damaging(0.824)
rs193922463	11:3659775811:36597758	C/A	-	SNP	-	Missense variant	N/K	968	11deleterious(0.01)	732possibly damaging(0.731)
COSM72375	11:3659776011:36597760	A/C	-	somatic_S	-	Missense variant	K/T	969	1deleterious(0)	732possibly damaging(0.731)
COSM926744	11:3659777111:36597771	C/T	-	somatic_S	-	Missense variant	R/C	973	1deleterious(0)	910probably damaging(0.909)
CM016160	11:3659777211:36597772	HGMD_MUTA	-	SNP	-	Coding sequence variant		973	-	-
A;vf=60925655;source=Pher	11:3659777211:36597772	G/A	-	SNP	-	Missense variant	R/H	973	1deleterious(0)	874possibly damaging(0.873)
rs121918570	11:3659777711:36597777	C/T	-	SNP	-	Missense variant	R/W	975	11deleterious(0.01)	910probably damaging(0.909)
CM081767	11:3659777711:36597777	HGMD_MUTA	-	SNP	-	Coding sequence variant		975	-	-
T;vf=60925656;source=Pher	11:3659777711:36597777	C/T	-	SNP	-	Missense variant	R/W	975	11deleterious(0.01)	910probably damaging(0.909)
COSM1507887	11:3659777711:36597777	C/A	-	somatic_S	-	Synonymous variant	R	975	-	-
CM010084	11:3659777811:36597778	HGMD_MUTA	-	SNP	-	Coding sequence variant		975	-	-
A;vf=60925605;source=Pher	11:3659777811:36597778	G/A	-	SNP	-	Missense variant	R/Q	975	1deleterious(0)	644possibly damaging(0.643)
CD099822	11:3659778911:36597789	HGMD_MUTA	-	deletion	-	Coding sequence variant		979	-	-
RAG1base_RAG1_DNA:g.92	11:3659778911:36597789-36597791	GCC/-	-	deletion	-	Inframe deletion	A/-	979	-	-
CM054093	11:3659779611:36597796	HGMD_MUTA	-	SNP	-	Coding sequence variant		981	-	-
C;vf=60925658;source=Pher	11:3659779611:36597796	A/C	-	SNP	-	Missense variant	Q/P	981	1deleterious(0)	545possibly damaging(0.544)
CM016161	11:3659782811:36597828	HGMD_MUTA	-	SNP	-	Coding sequence variant		992	-	-
G;vf=60925659;source=Pher	11:3659782811:36597828	A/G	-	SNP	-	Missense variant	K/E	992	1deleterious(0)	627possibly damaging(0.626)
CM065413	11:3659782911:36597829	HGMD_MUTA	-	SNP	-	Coding sequence variant		992	-	-
G;vf=60925660;source=Pher	11:3659782911:36597829	A/G	-	SNP	-	Missense variant	K/R	992	1deleterious(0)	627possibly damaging(0.626)
COSM1263745	11:3659783011:36597830	A/G	-	somatic_S	-	Synonymous variant	K	992	-	-
CM092639	11:3659787011:36597870	HGMD_MUTA	-	SNP	-	Coding sequence variant		1006	-	-
rs200043512	11:3659788011:36597880	A/G	-	SNP	-	Missense variant	H/R	1009	1deleterious(0)	629possibly damaging(0.628)
COSM1223038	11:3659791611:36597916	C/A	-	somatic_S	-	Missense variant	P/H	1021	1deleterious(0)	1benign(0)
COSM926745	11:3659792411:36597924	A/G	-	somatic_S	-	Missense variant	S/G	1024	231tolerated(0.23)	1benign(0)
COSM1289550	11:3659792611:36597926	C/T	-	somatic_S	-	Synonymous variant	S	1024	-	-
COSM322972	11:3659797511:36597975	A/T	-	somatic_S	-	Missense variant	M/L	1041	31deleterious(0.03)	84benign(0.083)
COSM212763	11:3659797811:36597978	G/T	-	somatic_S	-	Stop gained	E/*	1042	-	-
rs189589191	11:3659800911:36598009	G/A	0.001 (A)	SNP	-	3 prime UTR variant	-	-	-	-
rs141129968	11:3659832311: between 36598323 & 36598324	-/CAC	-	insertion	-	Feature elongation, 3 prime UTR var	-	-	-	-
rs35276810	11:3659856811: between 36598568 & 36598569	-/C	-	insertion	-	Feature elongation, 3 prime UTR var	-	-	-	-
rs149724031	11:3659916411:36599164	G/A	0.003 (A)	SNP	-	3 prime UTR variant	-	-	-	-
rs187884172	11:3659943611:36599436	G/A	0.001 (A)	SNP	-	3 prime UTR variant	-	-	-	-
rs185464049	11:3659954811:36599548	A/G	0.002 (G)	SNP	-	3 prime UTR variant	-	-	-	-
rs188175007	11:3659999611:36599996	T/A	0.001 (A)	SNP	-	3 prime UTR variant	-	-	-	-
rs35353578	11:3660005111:36600051	T/-	-	deletion	-	3 prime UTR variant, Feature trunca	-	-	-	-
rs200013770	11:3660027511:36600275	T/C	-	SNP	-	3 prime UTR variant	-	-	-	-
rs192931118	11:3660052711:36600527	G/A	0.001 (A)	SNP	-	3 prime UTR variant	-	-	-	-
rs184027330	11:3660080011:36600800	C/T	0.000 (T)	SNP	-	3 prime UTR variant	-	-	-	-
rs188367509	11:3660080311:36600803	C/T	0.001 (T)	SNP	-	3 prime UTR variant	-	-	-	-
rs199555129	11:3660092211:36600922-36600925	TAAG/-	0.011 (-)	deletion	-	3 prime UTR variant, Feature trunca	-	-	-	-
rs180966342	11:3660100611:36601006	T/G	0.001 (G)	SNP	-	3 prime UTR variant	-	-	-	-
rs143299264	11:3660101611:36601016-36601017	TA/-	-	deletion	-	3 prime UTR variant, Feature trunca	-	-	-	-
rs183729240	11:3660114211:36601142	A/G	0.002 (G)	SNP	-	3 prime UTR variant	-	-	-	-

rs190801060	11:3660120211:36601202	A/G	0.001 (G)	SNP	-	3 prime UTR variant	-	-	-	-
rs182094929	11:3660146311:36601463	A/G	0.003 (G)	SNP	-	Downstream gene variant	-	-	-	-
rs201747712	11:3660160011:36601600	C/T	-	SNP	-	Downstream gene variant	-	-	-	-
rs199609389	11:3660163311:36601633	C/T	-	SNP	-	Downstream gene variant	-	-	-	-
rs186982324	11:3660177011:36601770	G/A	0.008 (A)	SNP	-	Downstream gene variant	-	-	-	-
rs191430374	11:3660187411:36601874	C/A	0.008 (A)	SNP	-	Downstream gene variant	-	-	-	-
rs181256191	11:3660199711:36601997	A/G	0.001 (G)	SNP	-	Downstream gene variant	-	-	-	-
rs185362250	11:3660213911:36602139	G/A	0.001 (A)	SNP	-	Downstream gene variant	-	-	-	-
rs190038779	11:3660229411:36602294	C/G	0.001 (G)	SNP	-	Downstream gene variant	-	-	-	-
rs182324113	11:3660237011:36602370	C/T	0.001 (T)	SNP	-	Downstream gene variant	-	-	-	-
rs35587415	11:3660253411: between 36602534 & 36602535	-/C	-	insertion	-	Downstream gene variant	-	-	-	-
rs150250678	11:3660256511:36602565	T/A	0.001 (A)	SNP	-	Downstream gene variant	-	-	-	-
rs187577150	11:3660259511:36602595	G/A	0.001 (A)	SNP	-	Downstream gene variant	-	-	-	-
rs192292930	11:3660263711:36602637	A/G	0.001 (G)	SNP	-	Downstream gene variant	-	-	-	-
rs183672476	11:3660265711:36602657	G/A	0.001 (A)	SNP	-	Downstream gene variant	-	-	-	-
rs188719571	11:3660266711:36602667	A/T	0.001 (T)	SNP	-	Downstream gene variant	-	-	-	-
rs192039745	11:3660295111:36602951	G/A	0.001 (A)	SNP	-	Downstream gene variant	-	-	-	-
rs187305921	11:3660330011:36603300	G/T	0.001 (T)	SNP	-	Downstream gene variant	-	-	-	-
rs191751000	11:3660360811:36603608	T/C	0.001 (C)	SNP	-	Downstream gene variant	-	-	-	-
rs183964030	11:3660403111:36604031	T/G	0.001 (G)	SNP	-	Downstream gene variant	-	-	-	-
rs188987207	11:3660430311:36604303	C/T	0.003 (T)	SNP	-	Downstream gene variant	-	-	-	-
rs182437568	11:3660445611:36604456	C/T	0.001 (T)	SNP	-	Downstream gene variant	-	-	-	-
rs185429654	11:3660446911:36604469	T/C	0.001 (C)	SNP	-	Downstream gene variant	-	-	-	-
rs77373993	11:3660469811:36604698	-/TA	-	indel	-	Downstream gene variant	-	-	-	-
rs77373993	11:3660469811:36604698	-/TA	-	indel	-	Downstream gene variant	-	-	-	-
rs35263664	11:3660471411: between 36604714 & 36604715	-/A	-	insertion	-	Downstream gene variant	-	-	-	-
rs140423805	11:3660494911: between 36604949 & 36604950	-/C	0.017 (C)	insertion	-	Downstream gene variant	-	-	-	-
rs190332772	11:3660515311:36605153	T/C	0.001 (C)	SNP	-	Downstream gene variant	-	-	-	-
rs207471775	11:3660516911:36605169	A/C	-	SNP	-	Downstream gene variant	-	-	-	-
rs181635836	11:3660520511:36605205	T/C	0.001 (C)	SNP	-	Downstream gene variant	-	-	-	-
