# Supplementary material for: Data on the evolutionary history of the V(D)J recombination-activating protein 1 – RAG1 coupled with sequence and variant analyses
Source: Data Brief. 2016 May 20;8:87–92. doi: 10.1016/j.dib.2016.05.021 (PMC4887553; doi:10.1016/j.dib.2016.05.021)
Supplement: Supplementary file 3 — Supplementary material Table S2. Overview of non-coding germline variants of Human RAG1. [file mmc3.pdf]

Table S2. Summary of non-coding germline variants of Human RAG1.

Variant ID	Chromosomal position	Alleles	GMAF	Variant cla	status	Variant type	rSNP	LDP	PR	DR	miRR	RBP	eQ
rs12277745	***11:3658718011:36587180***	***C/T***	***0.018 (T)***	***SNP***	***MO,FR,HM,TG,***	***USG***	***y***	***y***	***y***	***n***	***n***	***y***	***y***
rs3758873	***11:3658701711:36587017***	***A/C***	***0.378 (C)***	***SNP***	***MO,FR,HM,TG,***	***USG***	***y***	***y***	***y***	***n***	***n***	***y***	***y***
rs4150999	***11:3658919311:36589193***	***G/C***	***0.021 (C)***	***SNP***	***MO,FR,HM,TG,***	***USG***	***y***	***y***	***y***	***n***	***n***	***y***	***y***
rs4151047	***11:3660051611:36600516***	***G/T***	***0.017 (T)***	***SNP***	***MO,FR,HM,TG,***	***3'U***	***y***	***y***	***y***	***n***	***n***	***y***	***y***
rs4151001	***11:3659011811:36590118***	***A/G***	***0.015 (G)***	***SNP***	***MO,FR,HM,TG,***	***intronic***	***y***	***y***	***n***	***n***	***n***	***y***	***y***
TMP_ESP_11_36594833	11:3659483311:36594833	G/A	-	SNP	ESP,	intronic	NA	NA	NA	NA	NA	NA	NA
TMP_ESP_11_36597993	11:3659799311:36597993	C/T	-	SNP	ESP,	3'U	NA	NA	NA	NA	NA	NA	NA
rs11033696	*11:3658521911:36585219*	*C/T*	*0.164 (T)*	*SNP*	*MO,FR,TG,*	*USG*	*y*	*y*	*y*	*y*	*n*	*y*	*n*
rs12282321	*11:3658461711:36584617*	*G/T*	*0.016 (T)*	*SNP*	*MO,FR,TG,*	*USG*	*y*	*y*	*y*	*y*	*n*	*y*	*n*
rs1515060	*11:3658632211:36586322*	*G/A*	*0.164 (A)*	*SNP*	*MO,FR,HM,TG,*	*USG*	*y*	*y*	*y*	*y*	*n*	*y*	*n*
rs7107464	*11:3658494411:36584944*	*T/C*	*0.183 (C)*	*SNP*	*MO,FR,HM,TG,*	*USG*	*y*	*y*	*y*	*y*	*n*	*y*	*n*
rs73453392	*11:3658469611:36584696*	*G/A*	*0.012 (A)*	*SNP*	*MO,FR,TG,*	*USG*	*y*	*y*	*y*	*y*	*n*	*y*	*n*
rs73453395	*11:3658479211:36584792*	*A/G*	*0.007 (G)*	*SNP*	*MO,FR,TG,*	*USG*	*y*	*y*	*y*	*y*	*n*	*y*	*n*
rs73453397	*11:3658576011:36585760*	*C/G*	*0.013 (G)*	*SNP*	*MO,FR,TG,*	*USG*	*y*	*y*	*y*	*y*	*n*	*y*	*n*
rs114432633	11:3658496111:36584961	T/G	0.013 (G)	SNP	FR,TG,	USG	y	n	y	y	n	y	n
rs115073791	11:3658632411:36586324	C/T	0.020 (T)	SNP	FR,TG,	USG	y	n	y	y	n	y	n
rs115620583	11:3658599511:36585995	A/G	0.003 (G)	SNP	FR,TG,	USG	y	n	y	y	n	y	n
rs115977457	11:3658546211:36585462	T/G	0.018 (G)	SNP	FR,TG,	USG	y	n	y	y	n	y	n
rs138113175	11:3658635211:36586352	C/G	0.004 (G)	SNP	TG,	USG	y	n	y	y	n	y	n
rs139494020	11:3658459811:36584598	C/G	0.006 (G)	SNP	TG,	USG	y	n	y	y	n	y	n
rs140777638	11:3658535711:36585357	G/T	0.006 (T)	SNP	TG,	USG	y	n	y	y	n	y	n
rs142639510	11:3658636611:36586366	C/T	0.004 (T)	SNP	TG,	USG	y	n	y	y	n	y	n
rs145046096	11:3658465411:36584654	C/T	0.001 (T)	SNP	TG,	USG	y	n	y	y	n	y	n
rs145584687	11:3658551011:36585510	C/T	0.001 (T)	SNP	TG,	USG	y	n	y	y	n	y	n
rs145589374	11:3658563111:36585631	A/G	0.001 (G)	SNP	TG,	USG	y	n	y	y	n	y	n
rs145640682	11:3658639611:36586396	T/C	0.001 (C)	SNP	TG,	USG	y	n	y	y	n	y	n
rs148076313	11:3658641911:36586419	T/A	0.001 (A)	SNP	TG,	USG	y	n	y	y	n	y	n
rs148875767	11:3658553611:36585536	C/G	0.001 (G)	SNP	TG,	USG	y	n	y	y	n	y	n
rs150102929	11:3658548111:36585481	A/G	0.001 (G)	SNP	-	USG	y	n	y	y	n	y	n
rs182154807	11:3658607711:36586077	A/G	0.001 (G)	SNP	-	USG	y	n	y	y	n	y	n
rs184525443	11:3658537811:36585378	T/C	0.002 (C)	SNP	-	USG	y	n	y	y	n	y	n
rs185543806	11:3658630011:36586300	C/T	0.001 (T)	SNP	-	USG	y	n	y	y	n	y	n
rs187301762	11:3658471211:36584712	G/T	0.001 (T)	SNP	-	USG	y	n	y	y	n	y	n
rs189029926	11:3658634011:36586340	C/T	0.001 (T)	SNP	-	USG	y	n	y	y	n	y	n
rs189260790	11:3658600911:36586009	T/G	0.001 (G)	SNP	-	USG	y	n	y	y	n	y	n
rs191748582	11:3658523711:36585237	G/A	0.003 (A)	SNP	-	USG	y	n	y	y	n	y	n

rs58012522	11:3658515111:36585151-36585154	AACT/-	-	deletion	MO,	USG	y	n	y	y	n	y	n
rs72236516	11:3658514811:36585148-36585151	ACTA/-	0.166 (-)	deletion	MO,	USG	y	n	y	y	n	y	n
rs74524795	11:3658568711:36585687	A/C	-	SNP	FR,	USG	y	n	y	y	n	y	n
rs1056403	*11:3660117011:36601170*	*G/A*	*0.412 (G)*	*SNP*	*MO,FR,HM,TG,*	*3'U*	*y*	*y*	*y*	*n*	*n*	*y*	*n*
rs12279639	*11:3658807811:36588078*	*G/C*	*0.018 (C)*	*SNP*	*MO,FR,TG,*	*USG*	*y*	*y*	*y*	*n*	*n*	*y*	*n*
rs2056094	*11:3658907111:36589071*	*G/C*	*0.069 (C)*	*SNP*	*MO,FR,HM,TG,*	*USG*	*y*	*y*	*y*	*n*	*n*	*y*	*n*
rs4150996	*11:3658840311:36588403*	*C/G/T*	*0.012 (T)*	*SNP*	*MO,FR,TG,*	*USG*	*y*	*y*	*y*	*n*	*n*	*y*	*n*
rs4150996	*11:3658840311:36588403*	*C/G/T*	*0.012 (T)*	*SNP*	*MO,FR,TG,*	*USG*	*y*	*y*	*y*	*n*	*n*	*y*	*n*
rs4150997	*11:3658892111:36588921*	*A/G*	*0.003 (G)*	*SNP*	*MO,FR,HM,TG,*	*USG*	*y*	*y*	*y*	*n*	*n*	*y*	*n*
rs4150998	*11:3658894711:36588947*	*G/A*	*0.003 (A)*	*SNP*	*MO,FR,HM,TG,*	*USG*	*y*	*y*	*y*	*n*	*n*	*y*	*n*
rs4151000	*11:3658934111:36589341*	*C/T*	*0.063 (T)*	*SNP*	*MO,FR,HM,TG,*	*USG*	*y*	*y*	*y*	*n*	*n*	*y*	*n*
rs4151036	*11:3659838411:36598384*	*G/A*	*0.010 (A)*	*SNP*	*MO,FR,HM,TG,*	*3'U*	*y*	*y*	*y*	*n*	*n*	*y*	*n*
rs4151038	*11:3659857511:36598575*	*G/A*	*0.010 (A)*	*SNP*	*MO,FR,HM,TG,*	*3'U*	*y*	*y*	*y*	*n*	*n*	*y*	*n*
rs4151040	*11:3659906911:36599069*	*C/T*	*0.070 (T)*	*SNP*	*MO,FR,HM,TG,*	*3'U*	*y*	*y*	*y*	*n*	*n*	*y*	*n*
rs4151041	*11:3659909011:36599090*	*A/G*	*0.003 (G)*	*SNP*	*MO,FR,HM,TG,*	*3'U*	*y*	*y*	*y*	*n*	*n*	*y*	*n*
rs4151042	*11:3659948011:36599480*	*A/C*	*0.004 (C)*	*SNP*	*MO,FR,TG,*	*3'U*	*y*	*y*	*y*	*n*	*n*	*y*	*n*
rs4151044	*11:3659996411:36599964*	*C/T*	*0.016 (T)*	*SNP*	*MO,FR,HM,TG,*	*3'U*	*y*	*y*	*y*	*n*	*n*	*y*	*n*
rs4151045	*11:3660023211:36600232*	*T/C*	*0.070 (C)*	*SNP*	*MO,FR,HM,TG,*	*3'U*	*y*	*y*	*y*	*n*	*n*	*y*	*n*
rs4151046	*11:3660031511:36600315*	*G/T*	*0.003 (T)*	*SNP*	*MO,FR,HM,TG,*	*3'U*	*y*	*y*	*y*	*n*	*n*	*y*	*n*
rs73453401	*11:3658676811:36586768*	*C/T*	*0.070 (T)*	*SNP*	*MO,FR,TG,*	*USG*	*y*	*y*	*y*	*n*	*n*	*y*	*n*
rs73455505	*11:3658726711:36587267*	*G/A*	*0.012 (A)*	*SNP*	*MO,FR,TG,*	*USG*	*y*	*y*	*y*	*n*	*n*	*y*	*n*
rs872052	*11:3658957311:36589573*	*G/A*	*0.001 (A)*	*SNP*	*MO,FR,TG,*	*USG*	*y*	*y*	*y*	*n*	*n*	*y*	*n*
rs872053	*11:3658962211:36589622*	*A/G*	*0.069 (G)*	*SNP*	*MO,FR,HM,TG,*	*5'U*	*y*	*y*	*y*	*n*	*n*	*y*	*n*
rs111610814	11:3658669211:36586692	A/G	0.065 (G)	SNP	MO,FR,TG,	USG	y	n	y	n	n	y	n
rs112766186	11:3659908611:36599086	C/T	-	SNP	FR,	3'U	y	n	y	n	n	y	n
rs113060327	11:3659926111:36599261	A/G	-	SNP	FR,	3'U	y	n	y	n	n	y	n
rs113291580	11:3658938711:36589387	G/C	-	SNP	FR,	USG	y	n	y	n	n	y	n
rs114563822	11:3658690811:36586908	C/T	0.025 (T)	SNP	MO,FR,TG,	USG	y	n	y	n	n	y	n
rs114954186	11:3658673111:36586731	A/G	0.020 (G)	SNP	FR,TG,	USG	y	n	y	n	n	y	n
rs114990708	11:3658783011:36587830	G/A	0.010 (A)	SNP	MO,FR,TG,	USG	y	n	y	n	n	y	n
rs115022045	11:3658918711:36589187	A/G	0.006 (G)	SNP	FR,TG,	USG	y	n	y	n	n	y	n
rs115387428	11:3658670611:36586706	G/A	0.018 (A)	SNP	FR,TG,	USG	y	n	y	n	n	y	n
rs115582302	11:3659842611:36598426	A/G	0.004 (G)	SNP	FR,TG,	3'U	y	n	y	n	n	y	n
rs117817879	11:3658954111:36589541	C/T	0.005 (T)	SNP	FR,TG,	USG	y	n	y	n	n	y	n
rs137987566	11:3658763211:36587632-36587633	AG/-	-	deletion	MO,	USG	y	n	y	n	n	y	n
rs139913944	11:3658703411:36587034	A/G	0.006 (G)	SNP	TG,	USG	y	n	y	n	n	y	n
rs139938937	11:3659915211:36599152	C/A	0.001 (A)	SNP	TG,	3'U	y	n	y	n	n	y	n

rs1399601	11:3658789111:36587891	G/C	-	SNP	MO,	USG	y	n	y	n	n	y	n
rs140212393	11:3658732511:36587325	A/C	0.001 (C)	SNP	TG,	USG	y	n	y	n	n	y	n
rs141384582	11:3660109711:36601097	T/-	-	deletion	MO,	3'U , FT	y	n	y	n	n	y	n
rs141913434	11:3658653411:36586534	T/C	0.001 (C)	SNP	TG,	USG	y	n	y	n	n	y	n
rs143196268	11:3658703711:36587037	C/A	0.002 (A)	SNP	TG,	USG	y	n	y	n	n	y	n
rs143299264	11:3660101611:36601016-36601017	TA/-	-	deletion	-	3'U , FT	y	n	y	n	n	y	n
rs143939152	11:3658648511:36586485-36586491	CGTTGTC/-	0.006 (-)	deletion	-	USG	y	n	y	n	n	y	n
rs144069419	11:3659825911:36598259	G/T	0.001 (C)	SNP	TG,	3'U	y	n	y	n	n	y	n
rs144104412	11:3658922311:36589223	G/T	0.001 (T)	SNP	TG,	USG	y	n	y	n	n	y	n
rs144896778	11:3658807611:36588076	G/C	0.006 (C)	SNP	TG,	USG	y	n	y	n	n	y	n
rs145552187	11:3659974711:36599747	G/A	0.003 (A)	SNP	TG,	3'U	y	n	y	n	n	y	n
rs145610899	11:3658735511:36587355	T/C	0.001 (C)	SNP	TG,	USG	y	n	y	n	n	y	n
rs145963034	11:3659908711:36599087	G/A	0.001 (A)	SNP	TG,	3'U	y	n	y	n	n	y	n
rs147500932	11:3658717411:36587174	G/T	0.001 (T)	SNP	TG,	USG	y	n	y	n	n	y	n
rs148483119	11:3660015111:36600151	T/C	0.002 (C)	SNP	TG,	3'U	y	n	y	n	n	y	n
rs148608607	11:3658829811:36588298	A/G	0.001 (G)	SNP	TG,	USG	y	n	y	n	n	y	n
rs149724031	11:3659916411:36599164	G/A	0.003 (A)	SNP	-	3'U	y	n	y	n	n	y	n
rs150640980	11:3658700711:36587007	T/A	0.002 (A)	SNP	-	USG	y	n	y	n	n	y	n
rs151163578	11:3658886411:36588864	C/A	0.005 (A)	SNP	-	USG	y	n	y	n	n	y	n
rs180966342	11:3660100611:36601006	T/G	0.001 (G)	SNP	-	3'U	y	n	y	n	n	y	n
rs181637298	11:3658685211:36586852	G/T	0.001 (T)	SNP	-	USG	y	n	y	n	n	y	n
rs183729240	11:3660114211:36601142	A/G	0.002 (G)	SNP	-	3'U	y	n	y	n	n	y	n
rs183964566	11:3658912311:36589123	A/G	0.002 (G)	SNP	-	USG	y	n	y	n	n	y	n
rs184027330	11:3660080011:36600800	C/T	0.000 (T)	SNP	-	3'U	y	n	y	n	n	y	n
rs185464049	11:3659954811:36599548	A/G	0.002 (G)	SNP	-	3'U	y	n	y	n	n	y	n
rs185816377	11:3658780711:36587807	G/A	0.001 (A)	SNP	-	USG	y	n	y	n	n	y	n
rs185970602	11:3658893411:36588934	A/G	0.001 (G)	SNP	-	USG	y	n	y	n	n	y	n
rs187884172	11:3659943611:36599436	G/A	0.001 (A)	SNP	-	3'U	y	n	y	n	n	y	n
rs188175007	11:3659999611:36599996	T/A	0.001 (A)	SNP	-	3'U	y	n	y	n	n	y	n
rs188367509	11:3660080311:36600803	C/T	0.001 (T)	SNP	-	3'U	y	n	y	n	n	y	n
rs189163766	11:3658916711:36589167	G/A	0.001 (A)	SNP	-	USG	y	n	y	n	n	y	n
rs189589191	11:3659800911:36598009	G/A	0.001 (A)	SNP	-	3'U	y	n	y	n	n	y	n
rs190278950	11:3658780811:36587808	C/A	0.001 (A)	SNP	-	USG	y	n	y	n	n	y	n
rs190801060	11:3660120211:36601202	A/G	0.001 (G)	SNP	-	3'U	y	n	y	n	n	y	n
rs192931118	11:3660052711:36600527	G/A	0.001 (A)	SNP	-	3'U	y	n	y	n	n	y	n
rs199555129	11:3660092211:36600922-36600925	TAAG/-	0.011 (-)	deletion	-	3'U , FT	y	n	y	n	n	y	n
rs200013770	11:3660027511:36600275	T/C	-	SNP	-	3'U	y	n	y	n	n	y	n

rs35353578	11:3660005111:36600051	T/-	-	deletion	-	3'U , FT	y	n	y	n	n	y	n
rs4151035	11:3659822411:36598224	G/A	-	SNP	FR,	3'U	y	n	y	n	n	y	n
rs4151037	11:3659840611:36598406	T/C	-	SNP	FR,	3'U	y	n	y	n	n	y	n
rs4151039	11:3659872511:36598725	C/T	-	SNP	FR,	3'U	y	n	y	n	n	y	n
rs4151043	11:3659991911:36599919	C/G	-	SNP	FR,	3'U	y	n	y	n	n	y	n
rs4151049	11:3660129311:36601293	A/-	-	deletion	FR,	3'U , FT	y	n	y	n	n	y	n
rs11033698	11:3660609311:36606093	T/G	0.147 (G)	SNP	MO,FR,HM,TG,	DSG	y	y	n	n	n	y	n
rs12275170	11:3660551611:36605516	C/A	0.003 (A)	SNP	MO,TG,	DSG	y	y	n	n	n	y	n
rs12421641	11:3660621911:36606219	C/A	0.104 (A)	SNP	MO,FR,HM,TG,	DSG	y	y	n	n	n	y	n
rs1515061	11:3659396711:36593967	A/T	0.001 (T)	SNP	MO,FR,TG,	intronic	y	y	n	n	n	y	n
rs16929067	11:3660573511:36605735	G/C	0.099 (C)	SNP	MO,FR,HM,TG,	DSG	y	y	n	n	n	y	n
rs2673017	11:3660131711:36601317	A/G	0.027 (G)	SNP	MO,FR,HM,TG,	DSG	y	y	n	n	n	y	n
rs4151002	11:3659021311:36590213	G/A	0.125 (A)	SNP	MO,FR,HM,TG,	intronic	y	y	n	n	n	y	n
rs4151004	11:3659086111:36590861	G/A	0.022 (A)	SNP	MO,FR,TG,	intronic	y	y	n	n	n	y	n
rs4151005	11:3659145711:36591457	A/G	0.035 (G)	SNP	MO,FR,HM,TG,	intronic	y	y	n	n	n	y	n
rs4151008	11:3659192011:36591920	A/G	0.012 (G)	SNP	MO,FR,TG,	intronic	y	y	n	n	n	y	n
rs4151011	11:3659208211:36592082	T/C	0.023 (C)	SNP	MO,FR,TG,	intronic	y	y	n	n	n	y	n
rs4151013	11:3659316411:36593164	C/T	0.019 (T)	SNP	MO,FR,TG,	intronic	y	y	n	n	n	y	n
rs4151014	11:3659336211:36593362	A/T	0.027 (T)	SNP	MO,FR,TG,	intronic	y	y	n	n	n	y	n
rs4151016	11:3659350611:36593506	T/C	0.003 (C)	SNP	MO,FR,HM,TG,	intronic	y	y	n	n	n	y	n
rs4151017	11:3659354311:36593543	G/A	0.005 (A)	SNP	MO,FR,HM,TG,	intronic	y	y	n	n	n	y	n
rs4151018	11:3659369111:36593691	A/G	0.005 (G)	SNP	MO,FR,HM,TG,	intronic	y	y	n	n	n	y	n
rs4151019	11:3659378811:36593788	C/T	0.003 (T)	SNP	MO,FR,HM,TG,	intronic	y	y	n	n	n	y	n
rs4151024	11:3659446011:36594460	T/G	0.016 (G)	SNP	MO,FR,HM,TG,	intronic	y	y	n	n	n	y	n
rs4151051	11:3660187211:36601872	G/A	0.106 (A)	SNP	MO,FR,TG,	DSG	y	y	n	n	n	y	n
rs61880060	11:3660206511:36602065	G/C	0.161 (C)	SNP	MO,FR,TG,	DSG	y	y	n	n	n	y	n
rs73455516	11:3660295811:36602958	C/T	0.070 (T)	SNP	MO,FR,TG,	DSG	y	y	n	n	n	y	n
rs73455517	11:3660306511:36603065	A/C	0.126 (C)	SNP	MO,FR,TG,	DSG	y	y	n	n	n	y	n
rs73455518	11:3660373311:36603733	A/G	0.070 (G)	SNP	MO,FR,TG,	DSG	y	y	n	n	n	y	n
rs7480202	11:3660513411:36605134	C/T	0.104 (T)	SNP	MO,FR,HM,TG,	DSG	y	y	n	n	n	y	n
rs7924442	11:3660316611:36603166	G/C	0.019 (C)	SNP	MO,TG,	DSG	y	y	n	n	n	y	n
rs111360078	11:3660314611:36603146	G/A	0.004 (A)	SNP	MO,FR,TG,	DSG	y	n	n	n	n	y	n
rs111728084	11:3660165611:36601656	A/G	0.088 (G)	SNP	MO,FR,TG,	DSG	y	n	n	n	n	y	n
rs111793475	11:3659116211:36591162	G/C	-	SNP	FR,	intronic	y	n	n	n	n	y	n
rs112079096	11:3660471211:36604712	A/G	0.009 (G)	SNP	MO,FR,TG,	DSG	y	n	n	n	n	y	n
rs112287696	11:3659240011:36592400	C/T	0.005 (T)	SNP	MO,FR,TG,	intronic	y	n	n	n	n	y	n
rs113179198	11:3660150111:36601501	C/T	0.005 (T)	SNP	MO,FR,TG,	DSG	y	n	n	n	n	y	n

rs113248703	11:3659256411:36592564	C/A	0.005 (A)	SNP	MO,FR,TG,	intronic	y	n	n	n	n	y	n
rs113270047	11:3660329711:36603297	C/T	0.126 (T)	SNP	MO,FR,TG,	DSG	y	n	n	n	n	y	n
rs11329058	11:3659170711:36591707	T/-	-	deletion	-	intronic	y	n	n	n	n	y	n
rs113502270	11:3659483511:36594835	T/C	-	SNP	FR,	intronic	y	n	n	n	n	y	n
rs113674944	11:3660137911:36601379	G/A	-	SNP	FR,	DSG	y	n	n	n	n	y	n
rs113728396	11:3660164711:36601647	C/T	-	SNP	FR,	DSG	y	n	n	n	n	y	n
rs114495988	11:3660396611:36603966	A/T	0.004 (T)	SNP	FR,TG,	DSG	y	n	n	n	n	y	n
rs114549317	11:3660375611:36603756	C/G	0.021 (G)	SNP	FR,TG,	DSG	y	n	n	n	n	y	n
rs114653635	11:3660324811:36603248	G/A	0.009 (A)	SNP	FR,TG,	DSG	y	n	n	n	n	y	n
rs115242321	11:3660351411:36603514	T/A	0.014 (A)	SNP	FR,TG,	DSG	y	n	n	n	n	y	n
rs115789056	11:3659221111:36592211	C/T	0.010 (T)	SNP	FR,TG,	intronic	y	n	n	n	n	y	n
rs116411362	11:3660219211:36602192	G/T	0.003 (T)	SNP	FR,TG,	DSG	y	n	n	n	n	y	n
rs117683643	11:3660290411:36602904	C/T	0.005 (T)	SNP	FR,TG,	DSG	y	n	n	n	n	y	n
rs118170873	11:3660390111:36603901	A/G	0.003 (G)	SNP	FR,TG,	DSG	y	n	n	n	n	y	n
rs138179285	11:3659036411:36590364	A/C	0.006 (C)	SNP	TG,	intronic	y	n	n	n	n	y	n
rs138443357	11:3659110411:36591104	T/C	0.001 (C)	SNP	TG,	intronic	y	n	n	n	n	y	n
rs138489163	11:3660620511:36606205	G/A	0.005 (A)	SNP	TG,	DSG	y	n	n	n	n	y	n
rs138870142	11:3660273811:36602738	A/T	0.002 (T)	SNP	TG,	DSG	y	n	n	n	n	y	n
rs139141414	11:3660420411:36604204	C/T	0.002 (T)	SNP	TG,	DSG	y	n	n	n	n	y	n
rs139477887	11:3659239211:36592392	A/C	0.001 (C)	SNP	TG,	intronic	y	n	n	n	n	y	n
rs139878501	11:3660394511:36603945	C/T	0.004 (T)	SNP	TG,	DSG	y	n	n	n	n	y	n
rs139907436	11:3660441011:36604410	G/T	0.009 (T)	SNP	TG,	DSG	y	n	n	n	n	y	n
rs140539498	11:3660598211:36605982	A/C	0.001 (C)	SNP	TG,	DSG	y	n	n	n	n	y	n
rs140971504	11:3660250411:36602504	C/T	0.001 (T)	SNP	TG,	DSG	y	n	n	n	n	y	n
rs141124967	11:3658991311:36589913	A/G	0.001 (G)	SNP	TG,	intronic	y	n	n	n	n	y	n
rs141180078	11:3660410011:36604100	G/A	0.016 (A)	SNP	MO,TG,	DSG	y	n	n	n	n	y	n
rs141557519	11:3659128111:36591281-36591285	AATAA/-	0.012 (-)	deletion	-	intronic	y	n	n	n	n	y	n
rs142236848	11:3659449111:36594491	C/A	0.001 (A)	SNP	TG,	intronic	y	n	n	n	n	y	n
rs142254848	11:3659068411:36590684-36590686	GCT/-	-	deletion	-	intronic	y	n	n	n	n	y	n
rs142337839	11:3660328411:36603284	T/A	0.004 (A)	SNP	TG,	DSG	y	n	n	n	n	y	n
rs142774263	11:3659379811:36593798	A/-	-	deletion	-	intronic	y	n	n	n	n	y	n
rs143081800	11:3659060911:36590609	G/C	0.001 (C)	SNP	TG,	intronic	y	n	n	n	n	y	n
rs143285432	11:3660522611:36605226	C/T	0.001 (T)	SNP	TG,	DSG	y	n	n	n	n	y	n
rs144021230	11:3659380411:36593804	G/C	0.002 (C)	SNP	TG,	intronic	y	n	n	n	n	y	n
rs144137229	11:3659175111:36591751	A/G	0.000 (G)	SNP	-	intronic	y	n	n	n	n	y	n
rs144872922	11:3659024511:36590245	G/A	0.001 (A)	SNP	TG,	intronic	y	n	n	n	n	y	n
rs145930516	11:3660344511:36603445	C/G	0.001 (G)	SNP	TG,	DSG	y	n	n	n	n	y	n

rs146498720	11:3659392111:36593921	C/T	0.001 (T)	SNP	TG,	intronic	y	n	n	n	n	y	n
rs147510686	11:3659095011:36590950	A/G	0.003 (G)	SNP	TG,	intronic	y	n	n	n	n	y	n
rs147659805	11:3660484511:36604845	C/T	0.004 (T)	SNP	TG,	DSG	y	n	n	n	n	y	n
rs147887098	11:3660140511:36601405	A/G	0.001 (G)	SNP	MO,TG,	DSG	y	n	n	n	n	y	n
rs148329600	11:3659054811:36590548	T/C	0.001 (A)	SNP	TG,	intronic	y	n	n	n	n	y	n
rs149133410	11:3659041011:36590410	G/A	0.001 (A)	SNP	TG,	intronic	y	n	n	n	n	y	n
rs149165412	11:3660520811:36605208	T/C	0.001 (C)	SNP	TG,	DSG	y	n	n	n	n	y	n
rs149442029	11:3660275811:36602758	G/A	0.008 (A)	SNP	TG,	DSG	y	n	n	n	n	y	n
rs150250678	11:3660256511:36602565	T/A	0.001 (A)	SNP	-	DSG	y	n	n	n	n	y	n
rs151218623	11:3659452811:36594528	T/A	0.001 (A)	SNP	-	intronic	y	n	n	n	n	y	n
rs181022935	11:3659298911:36592989	A/G	0.001 (G)	SNP	-	intronic	y	n	n	n	n	y	n
rs181256191	11:3660199711:36601997	A/G	0.001 (G)	SNP	-	DSG	y	n	n	n	n	y	n
rs181635836	11:3660520511:36605205	T/C	0.001 (C)	SNP	-	DSG	y	n	n	n	n	y	n
rs182094929	11:3660146311:36601463	A/G	0.003 (G)	SNP	-	DSG	y	n	n	n	n	y	n
rs182324113	11:3660237011:36602370	C/T	0.001 (T)	SNP	-	DSG	y	n	n	n	n	y	n
rs182437568	11:3660445611:36604456	C/T	0.001 (T)	SNP	-	DSG	y	n	n	n	n	y	n
rs183012850	11:3659352611:36593526	C/T	0.001 (T)	SNP	-	intronic	y	n	n	n	n	y	n
rs183093085	11:3659052311:36590523	G/A	0.002 (A)	SNP	-	intronic	y	n	n	n	n	y	n
rs183176795	11:3658978011:36589780	A/G	0.001 (G)	SNP	-	intronic	y	n	n	n	n	y	n
rs183216792	11:3659301511:36593015	G/C	0.005 (C)	SNP	-	intronic	y	n	n	n	n	y	n
rs183672476	11:3660265711:36602657	G/A	0.001 (A)	SNP	-	DSG	y	n	n	n	n	y	n
rs183964030	11:3660403111:36604031	T/G	0.001 (G)	SNP	-	DSG	y	n	n	n	n	y	n
rs184240786	11:3659128911:36591289	A/G	0.005 (G)	SNP	-	intronic	y	n	n	n	n	y	n
rs184503790	11:3659147411:36591474	G/A	0.001 (A)	SNP	-	intronic	y	n	n	n	n	y	n
rs184560088	11:3659392211:36593922	G/A	0.002 (A)	SNP	-	intronic	y	n	n	n	n	y	n
rs185100010	11:3659402211:36594022	C/T	0.001 (T)	SNP	-	intronic	y	n	n	n	n	y	n
rs185265982	11:3659300911:36593009	C/T	0.001 (T)	SNP	-	intronic	y	n	n	n	n	y	n
rs185362250	11:3660213911:36602139	G/A	0.001 (A)	SNP	-	DSG	y	n	n	n	n	y	n
rs185429654	11:3660446911:36604469	T/C	0.001 (C)	SNP	-	DSG	y	n	n	n	n	y	n
rs186126515	11:3659321811:36593218	G/A	0.003 (A)	SNP	-	intronic	y	n	n	n	n	y	n
rs186641408	11:3659352711:36593527	G/A	0.001 (A)	SNP	-	intronic	y	n	n	n	n	y	n
rs186982324	11:3660177011:36601770	G/A	0.008 (A)	SNP	-	DSG	y	n	n	n	n	y	n
rs187305921	11:3660330011:36603300	G/T	0.001 (T)	SNP	-	DSG	y	n	n	n	n	y	n
rs187498735	11:3659007211:36590072	T/C	0.001 (C)	SNP	-	intronic	y	n	n	n	n	y	n
rs187577150	11:3660259511:36602595	G/A	0.001 (A)	SNP	-	DSG	y	n	n	n	n	y	n
rs187642373	11:3659067811:36590678	T/C	0.001 (C)	SNP	-	intronic	y	n	n	n	n	y	n
rs188380538	11:3659344811:36593448	A/G	0.001 (G)	SNP	-	intronic	y	n	n	n	n	y	n

rs188719571	11:3660266711:36602667	A/T	0.001 (T)	SNP	-	DSG	y	n	n	n	n	y	n
rs188757378	11:3659150011:36591500	C/G	0.001 (G)	SNP	-	intronic	y	n	n	n	n	y	n
rs188987207	11:3660430311:36604303	C/T	0.003 (T)	SNP	-	DSG	y	n	n	n	n	y	n
rs189005815	11:3659392711:36593927	T/C	0.001 (C)	SNP	-	intronic	y	n	n	n	n	y	n
rs189467851	11:3659138611:36591386	G/A	0.001 (A)	SNP	-	intronic	y	n	n	n	n	y	n
rs189475567	11:3659178011:36591780	G/T	0.001 (T)	SNP	-	intronic	y	n	n	n	n	y	n
rs189996292	11:3659443311:36594433	G/C	0.001 (C)	SNP	-	intronic	y	n	n	n	n	y	n
rs190038779	11:3660229411:36602294	C/G	0.001 (G)	SNP	-	DSG	y	n	n	n	n	y	n
rs190332772	11:3660515311:36605153	T/C	0.001 (C)	SNP	-	DSG	y	n	n	n	n	y	n
rs190968516	11:3658968411:36589684	A/G	0.001 (G)	SNP	-	intronic	y	n	n	n	n	y	n
rs191430374	11:3660187411:36601874	C/A	0.008 (A)	SNP	-	DSG	y	n	n	n	n	y	n
rs191690982	11:3659335311:36593353	A/T	0.001 (T)	SNP	-	intronic	y	n	n	n	n	y	n
rs191751000	11:3660360811:36603608	T/C	0.001 (C)	SNP	-	DSG	y	n	n	n	n	y	n
rs191783409	11:3659048111:36590481	T/C	0.001 (C)	SNP	-	intronic	y	n	n	n	n	y	n
rs192039745	11:3660295111:36602951	G/A	0.001 (A)	SNP	-	DSG	y	n	n	n	n	y	n
rs192292930	11:3660263711:36602637	A/G	0.001 (G)	SNP	-	DSG	y	n	n	n	n	y	n
rs192467736	11:3659143411:36591434	A/G	0.001 (G)	SNP	-	intronic	y	n	n	n	n	y	n
rs192626550	11:3659078811:36590788	G/C	0.001 (C)	SNP	-	intronic	y	n	n	n	n	y	n
rs199609389	11:3660163311:36601633	C/T	-	SNP	-	DSG	y	n	n	n	n	y	n
rs200252068	11:3659048411:36590484	C/A	-	SNP	-	intronic	y	n	n	n	n	y	n
rs200979608	11:3659112311:36591123-36591124	AA/-	-	deletion	-	intronic	y	n	n	n	n	y	n
rs201395382	11:3659190311:36591903	T/C	-	SNP	-	intronic	y	n	n	n	n	y	n
rs201501543	11:3659481411:36594814	A/C	0.001 (C)	SNP	-	intronic	y	n	n	n	n	y	n
rs201747712	11:3660160011:36601600	C/T	-	SNP	-	DSG	y	n	n	n	n	y	n
rs201800589	11:3659128011:36591280-36591282	TAA/-	0.012 (-)	deletion	-	intronic	y	n	n	n	n	y	n
rs207471775	11:3660516911:36605169	A/C	-	SNP	-	DSG	y	n	n	n	n	y	n
rs2673016	11:3660278311:36602783	C/T	-	SNP	MO,	DSG	y	n	n	n	n	y	n
rs4151003	11:3659039711:36590397	A/G	-	SNP	FR,	intronic	y	n	n	n	n	y	n
rs4151006	11:3659176111:36591761	C/T	0.001 (T)	SNP	MO,FR,TG,	intronic	y	n	n	n	n	y	n
rs4151007	11:3659177611:36591776	T/C	0.002 (C)	SNP	MO,FR,HM,TG,	intronic	y	n	n	n	n	y	n
rs4151009	11:3659200711:36592007	A/G	-	SNP	FR,	intronic	y	n	n	n	n	y	n
rs4151010	11:3659208111:36592081	A/T	-	SNP	FR,	intronic	y	n	n	n	n	y	n
rs4151012	11:3659301111:36593011	T/C	0.002 (C)	SNP	MO,FR,HM,TG,	intronic	y	n	n	n	n	y	n
rs4151015	11:3659343111:36593431	G/A	0.002 (A)	SNP	MO,FR,TG,	intronic	y	n	n	n	n	y	n
rs4151020	11:3659388811:36593888	G/T	-	SNP	FR,	intronic	y	n	n	n	n	y	n
rs4151021	11:3659401111:36594011	C/A	-	SNP	FR,	intronic	y	n	n	n	n	y	n
rs4151022	11:3659414711:36594147	C/T	-	SNP	MO,FR,	intronic	y	n	n	n	n	y	n

rs4151023	11:3659441711:36594417	A/G	0.010 (G)	SNP	MO,FR,HM,TG,	intronic	y	n	n	n	n	y	n
rs4151050	11:3660171111:36601711	G/A	-	SNP	FR,	DSG	y	n	n	n	n	y	n
rs55679809	11:3659274411:36592744	C/T	-	SNP	-	intronic	y	n	n	n	n	y	n
rs72124072	11:3660628211:36606282	A/-	0.126 (-)	deletion	MO,	DSG	y	n	n	n	n	y	n
rs74534829	11:3659353011:36593530	G/T	0.006 (T)	SNP	MO,FR,TG,	intronic	y	n	n	n	n	y	n
rs75083727	11:3659373611:36593736	C/A	0.004 (A)	SNP	MO,FR,TG,	intronic	y	n	n	n	n	y	n
rs76254210	11:3659248911:36592489	T/C	-	SNP	MO,FR,	intronic	y	n	n	n	n	y	n
rs76511022	11:3660433311:36604333	T/C	0.016 (C)	SNP	MO,TG,	DSG	y	n	n	n	n	y	n
rs76511123	11:3660417911:36604179	A/T	0.001 (T)	SNP	TG,	DSG	y	n	n	n	n	y	n
rs78967763	11:3660370611:36603706	C/G	0.018 (G)	SNP	FR,TG,	DSG	y	n	n	n	n	y	n
rs79486620	11:3660608711:36606087	A/G	0.005 (G)	SNP	TG,	DSG	y	n	n	n	n	y	n
rs111789593	11:3659230011:36592300	T/A	-	SNP	FR,	intronic	n	n	n	n	n	n	n
rs111978058	11:3660329811: between 36603298 & 366	-/G	-	insertion	MO,	DSG	n	n	n	n	n	n	n
rs112440348	11:3659240011:36592400	G/C	-	SNP	-	intronic	n	n	n	n	n	n	n
rs140423805	11:3660494911: between 36604949 & 366	-/C	0.017 (C)	insertion	-	DSG	n	n	n	n	n	n	n
rs141129968	11:3659832311: between 36598323 & 365	-/CAC	-	insertion	-	3'U, FE	n	n	n	n	n	n	n
rs200068995	11:3659248911:36592489	G/A	-	SNP	-	intronic	n	n	n	n	n	n	n
rs35085546	11:3660330211: between 36603302 & 366	-/G	-	insertion	MO,FR,	DSG	n	n	n	n	n	n	n
rs35263664	11:3660471411: between 36604714 & 366	-/A	-	insertion	-	DSG	n	n	n	n	n	n	n
rs35276810	11:3659856811: between 36598568 & 365	-/C	-	insertion	-	3'U, FE	n	n	n	n	n	n	n
rs35587415	11:3660253411: between 36602534 & 366	-/C	-	insertion	-	DSG	n	n	n	n	n	n	n
rs35678890	11:3659433211: between 36594332 & 365	-/C	-	insertion	-	intronic	n	n	n	n	n	n	n
rs4151048	11:3660068411: between 36600684 & 366	-/T	0.071 (T)	insertion	MO,FR,	3'U, FE	n	n	n	n	n	n	n
rs76177616	11:3659244611:36592446	G/A	-	SNP	MO,	intronic	n	n	n	n	n	n	n
rs76603665	11:3659248211:36592482	G/A	-	SNP	-	intronic	n	n	n	n	n	n	n
rs79295607	11:3659245411:36592454	T/C	-	SNP	-	intronic	n	n	n	n	n	n	n

3'U - 3' UTR

5'U - 5' UTR

DR- Distal regulation

DSG - Downstream gene

eQ -Expressed QTL

FE - Feature Elongation

FR - Frequency

FT - Feature truncation

HM - HapMap

LDP - LD-proxy of rSNP(r2>0.8)

miRR - miRNA regulation

MO - Multiple_observations
n - no
NA - Not Available
PR - Proximal regulation
RBPMR- RNA binding protein mediated regulation
TG - 1000Genomes
USG- Upstream gene
y - yes
